# 2021 ISHNE/HRS/EHRA/APHRS Expert Collaborative Statement on mHealth in Arrhythmia Management: Digital Medical Tools for Heart Rhythm Professionals: From the International Society for Holter and Noninvasive Electrocardiology/Heart Rhythm Society/European Heart Rhythm Association/Asia-Pacific Heart Rhythm Society

**DOI:** 10.1161/CIRCEP.120.009204

**Published:** 2021-02-12

**Authors:** Niraj Varma, Iwona Cygankiewicz, Mintu P. Turakhia, Hein Heidbuchel, Yu-Feng Hu, Lin Yee Chen, Jean-Philippe Couderc, Edmond M. Cronin, Jerry D. Estep, Lars Grieten, Deirdre A. Lane, Reena Mehra, Alex Page, Rod Passman, Jonathan P. Piccini, Ewa Piotrowicz, Ryszard Piotrowicz, Pyotr G. Platonov, Antonio Luiz Ribeiro, Robert E. Rich, Andrea M. Russo, David Slotwiner, Jonathan S. Steinberg, Emma Svennberg

**Affiliations:** 1 Cleveland Clinic, OH (N.V., J.D.E., R.M., R.E.R.).; 2 Medical University of Lodz, Poland (I.C.).; 3 Stanford University, Palo Alto, CA (M.P.T.).; 4 Antwerp University and University Hospital, Belgium (H.H.).; 5 Taipei Veterans General Hospital, Taiwan (Y.-F.H.).; 6 University of Minnesota, Minneapolis (L.Y.C.).; 7 University of Rochester, NY (J.-P.C., A.P., J.S.S.).; 8 Temple University, Philadelphia, PA (E.M.C.).; 9 Hasselt University, Belgium (L.G.).; 10 University of Liverpool, United Kingdom (D.A.L.).; 11 Northwestern University Feinberg School of Medicine, Chicago, IL (R. Passman).; 12 Duke University, Durham, NC (J.P.P.).; 13 National Institute of Cardiology, Warsaw, Poland (E.P., R. Piotrowicz).; 14 Lund University, Sweden (P.G.P.).; 15 Faculdade de Medicina, Centro de Telessaúde, Hospital das Clínicas, and Departamento de Clínica Médica, Universidade Federal de Minas Gerais, Belo Horizonte, Brazil (A.L.R.).; 16 Cooper Medical School of Rowan University, Camden, NJ (A.M.R.).; 17 Cardiology Division, New York-Presbyterian Queens, NY (D.S.).; 18 Karolinska University Hospital, Stockholm, Sweden (E.S.).

**Keywords:** arrhythmias, atrial fibrillation, comorbidities, digital medicine, heart rhythm, mHealth

## Abstract

This collaborative statement from the International Society for Holter and Noninvasive Electrocardiology/Heart Rhythm Society/European Heart Rhythm Association/Asia-Pacific Heart Rhythm Society describes the current status of mobile health technologies in arrhythmia management. The range of digital medical tools and heart rhythm disorders that they may be applied to and clinical decisions that may be enabled are discussed. The facilitation of comorbidity and lifestyle management (increasingly recognized to play a role in heart rhythm disorders) and patient self-management are novel aspects of mobile health. The promises of predictive analytics but also operational challenges in embedding mobile health into routine clinical care are explored.

## Abstract 2

Supplemental Digital Content is available in the text.

## Table of Contents

1. INTRODUCTION 236
2. mHEALTH TECHNOLOGIES 238
  2.1. Ambulatory ECG Monitoring 238
  2.2. New mHealth-Based Modalities for Arrhythmia Monitoring 239
    2.2.1. ECG Based 240
      2.2.1.1. Handheld Devices 240
      2.2.1.2. Wearable Patches 240
      2.2.1.3. Biotextiles 241
      2.2.1.4. Smartphone- and Smartwatch-Based Devices 242
    2.2.2. Non–ECG Based 243
      2.2.2.1. Photoplethysmography 243
      2.2.2.2. Oscillometry 244
      2.2.2.3. Mechanocardiography 244
      2.2.2.4. Contactless Video Plethysmography 244
      2.2.2.5. Smart Speakers 244
3. mHEALTH APPLICATIONS FOR ARRHYTHMIAS 247
  3.1. Atrial Fibrillation 247
    3.1.1. Undiagnosed AF Identification 247
    3.1.2. Targeted Identification in High-Risk Individuals 248
    3.1.3. Diagnostics in People With Established Atrial Fibrillation 249
    3.1.4. AF Therapy 249
  3.2. Sudden Cardiac Death 250
4. COMORBIDITIES 256
  4.1. Ischemic Heart Disease 256
  4.2. Heart Failure 258
    4.2.1. Mobile Technologies for Managing HF 258
    4.2.2. Hybrid Telerehabilitation in Patients With HF 258
  4.3. Diabetes 259
  4.4. Hypertension 259
  4.5. Disorders Including Sleep Apnea 260
  4.6. Lifestyle 260
    4.6.1. Physical Activity 260
    4.6.2. Diet 261
5. PATIENT SELF-MANAGEMENT—INTEGRATED CHRONIC CARE 266
  5.1. Patient Engagement 266
  5.2. Behavioral Modification 266
  5.3. Patients as Part of a Community 267
  5.4. Maintaining Patient Engagement 267
  5.5. Digital Divide 268
6. CLINICAL TRIALS 270
7. OPERATIONAL CHALLENGES 272
  7.1. Healthcare System—eHealth Monitoring and Hospital Ecosystem 272
  7.2. Cybersecurity Guidance for mHealth Devices 273
    7.2.1. Hacking Strategies and Methods in mHealth Technologies 274
    7.2.2. Recommendations to the Manufacturer 274
    7.2.3. Recommendations to Clinicians and Administrator 274
    7.2.4. Recommendations to Patients 274
  7.3. Reimbursement 274
  7.4. Regulatory Landscape for mHealth Devices 275
8. Predictive Analytics 277
9. Future Directions 278

## 1. INTRODUCTION

### Document Scope and Rationale

Digital health is an umbrella term to describe the use of digital information, data, and communication technologies to collect, share, and analyze health information to improve patient health, education, and health care delivery (https://www.fcc.gov/general/5-questions-you-can-ask-your-doctor-about-digital-health; Turakhia 2016).^1,2^ This concept encompasses telehealth, electronic health records (EHRs), implantable device monitoring, wearable sensor data, analytics and artificial intelligence (AI), behavioral health, and personalized medicine. Among these, mobile health (mHealth) is a component of digital health, defined by the World Health Organization—as medical and public health practice supported by mobile devices, such as mobile phones, patient-monitoring devices, personal digital assistants, and other wireless devices (https://www.who.int/goe/publications/goe_mhealth_web.pdf; https://apps.who.int/gb/ebwha/pdf_files/WHA71/A71_20-en.pdf?ua=1).^3,4^ Utilization of these devices has proliferated among health-conscious consumers in recent years and is likely to continue rapid expansion and integration into more formalized medical settings.

mHealth flows intuitively to health professionals in the field of arrhythmia management from experience gained through remote monitoring (RM) of cardiovascular implantable electronic devices (CIEDs), such as pacemakers and implantable cardioverter-defibrillators (ICDs; Varma 2010).^5^ A wealth of data garnered from many studies over the last 10 to 15 years has confirmed the benefits of remote technology-assisted follow-up and established it as a standard of care (Varma 2013, Slotwiner 2015).^6,7^ However, results of RM of CIEDs may not be immediately generalizable to mHealth. For instance, the former is restricted to those with cardiac disease (largely arrhythmias and heart failure [HF]), that is, a group already defined as patients. The care pathways for CIED RM are also well defined, with billing and reimbursement in place in the United States and many other parts of the world. In comparison, mHealth differs: it is widely available in the form of consumer products that penetrate most sectors of society, including individuals without formal medical diagnoses; it may be applied to a wider group of medical conditions; data can be self-monitored rather than assessed by health care professionals (HCPs); and reimbursement models are not mature. Indeed, some heart rhythm tracking capabilities may be indirectly acquired in products purchased for different goals and then subsequently used for self-monitoring. Conversely, in the medical space, applications are largely not prescribed by HCPs, often lack validation for disease management use cases, and care pathways remain varied or poorly defined. Nevertheless, if properly implemented, the intersection of these two communities opens up a broad spectrum of opportunities, extending from population screening and surveillance for undiagnosed disease to longitudinal disease management and importantly, engaging patients in their own cycle of care, allowing much health care to be asynchronous and virtualized. Its value and degree of integration will depend on different health care systems in different countries (Table 1).

**Table 1.**
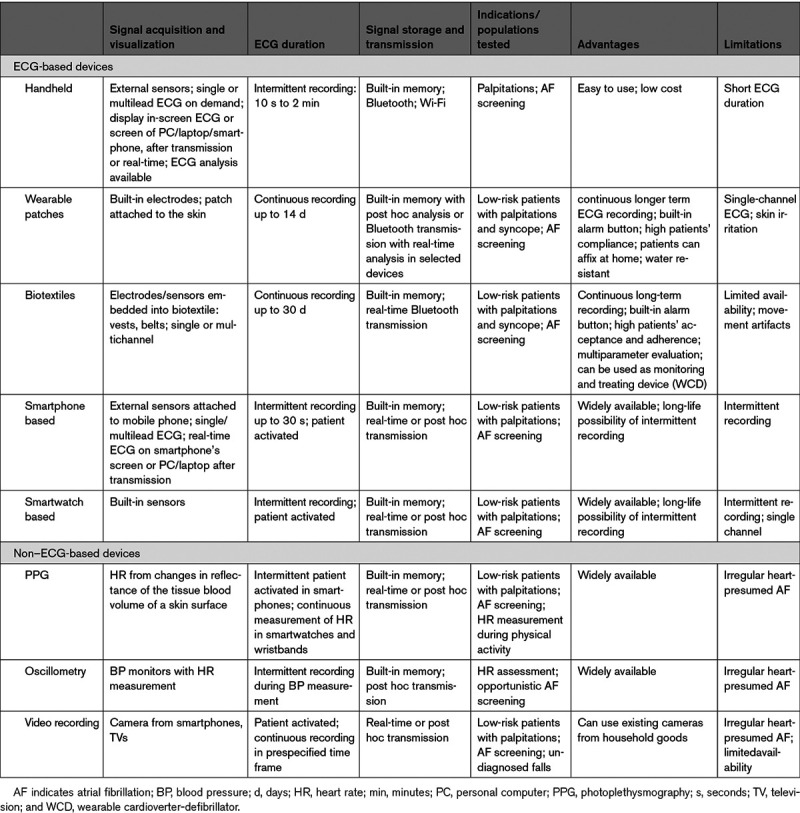
Mobile Health–Based Modalities for Arrhythmia Monitoring

mHealth has value only if the acquired information leads to decisions that improve outcome. This requires a clear path of information flow and actionability. Moreover, all stakeholders need to be aware of the logistical chain (so that everyone knows what to expect) and responsibilities clearly defined (possibly including device vendors). Similarly, actions taken based on the monitored information should be transparent to all stakeholders. For example, for a patient who records and transmits an irregular heart rhythm via a wearable device, a designated decision process should be followed to confirm, for example, whether the rhythm is atrial fibrillation (AF) or not, whether confirmation by another diagnostic test is required, how that is arranged, and finally, what therapy should be implemented and in what reasonable time frame? Clearly, there are risks of increasing cost from medical testing and provoking anxiety in consumers—who by virtue of seeking a medical verification become patients. Again, CIED experience sets a precedent. Studies that have shown improved outcome with telemonitoring succeeded when integrated into a clear logistical framework for a specific use case of disease management (eg, IN-TIME for RM in patients receiving cardiac resynchronization therapy, CardioMEMS; Abraham 2011, Hindricks 2014, Varma 2013).^6,8,9^ Replicating this with mHealth creates challenges for health care providers and goes far beyond the technological capabilities of the monitoring and transmission equipment. Implementation will require defined aims and fundamental changes to existing workflows and responsibilities. Such changes are always difficult. Apart from the organizational issues required to achieve such changes, reimbursement may drive or hinder such changes in the workplace. Awareness of these factors has been heightened by the SARS-CoV-2 (severe acute respiratory syndrome coronavirus 2) pandemic, during which telemedicine solutions have been advocated to reduce patient contact with health care providers yet continue health care delivery (Varma 2020).^10^

In view of the rapid technological development and popularity of wearable and other mobile devices, and the need for analysis and planning of the mHealth infrastructure, the International Society for Holter and Noninvasive Electrocardiology, Heart Rhythm Society (HRS), European Heart Rhythm Association, and Asia-Pacific HRS recognized the need for this collaborative statement. The aim of this document is to define state-of-the-art mHealth technologies and their application in arrhythmia management and explore future directions for clinical application. As such, the scope of the document encompasses discussion of the different mHealth technologies currently available or in development; the acquisition of health-related data; the applications of such data, including disease identification and management; clinical trials; the patient perspective; and the issues that must be addressed in the future to permit useful application of mHealth technologies. Additionally, discussion is extended to mHealth facilitation of those comorbidities increasingly recognized to influence arrhythmia management (eg, obesity and sleep apnea) that are becoming the responsibility of heart rhythm professionals (Chung 2020).^11^

**Figure.**
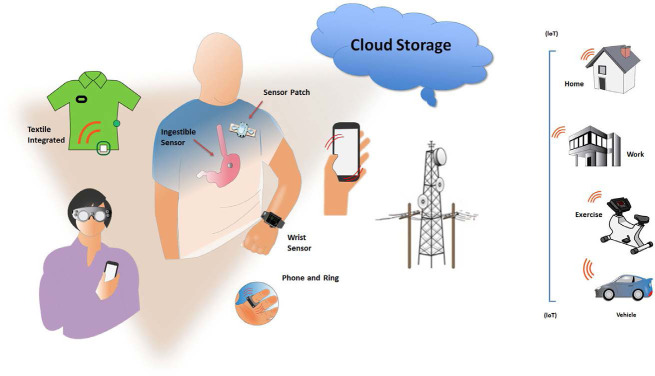
**mHealth tools for the individual.** Sensors can be embedded in a variety of wearables. IoT indicates Internet of things—connects from any location to hospital or cloud; see Table 1.

## 

References: Section 11.Federal Communications Commission (FCC). Five Questions You Can Ask Your Doctor about Digital Health.
Accessed January 26, 2021. https://www.fcc.gov/general/five-questions-you-can-ask-your-doctor-about-digital-health#ab2.TurakhiaMPDesaiSAHarringtonRA
The outlook of digital health for cardiovascular medicine: challenges but also extraordinary opportunities.
JAMA Cardiol. 2016;1:743–744. doi: 10.1001/jamacardio.2016.26612758027510.1001/jamacardio.2016.26613.World Health Organization. mHealth new horizons for health through mobile technologies.
2011
Accessed January 26, 2021. https://www.who.int/goe/publications/goe_mhealth_web.pdf4.World Health Organization. mHealth: use of appropriate digital technologies for public health.
2018
Accessed January 26, 2021. https://apps.who.int/gb/ebwha/pdf_files/WHA71/A71_20-en.pdf5.VarmaNEpsteinAEIrimpenASchweikertRLoveC; TRUST Investigators. Efficacy and safety of automatic remote monitoring for implantable cardioverter-defibrillator follow-up: the Lumos-T Safely Reduces Routine Office Device Follow-Up (TRUST) trial.
Circulation. 2010;122:325–332. doi: 10.1161/CIRCULATIONAHA.110.9374092062511010.1161/CIRCULATIONAHA.110.9374096.VarmaNRicciRP
Telemedicine and cardiac implants: what is the benefit?
Eur Heart J. 2013;34:1885–1895. doi: 10.1093/eurheartj/ehs3882321123110.1093/eurheartj/ehs388PMC40512587.SlotwinerDJAbrahamRLAl-KhatibSMAndersonHVBunchTJFerraraMGWilkoffBL
HRS white paper on interoperability of data from cardiac implantable electronic devices (CIEDs).
Heart Rhythm. 2019;16:e107–e127. doi: 10.1016/j.hrthm.2019.05.0023107780110.1016/j.hrthm.2019.05.0028.AbrahamWTAdamsonPBBourgeRCAaronMFCostanzoMRStevensonLWStricklandWNeelagaruSRavalNKruegerS; CHAMPION Trial Study Group. Wireless pulmonary artery haemodynamic monitoring in chronic heart failure: a randomised controlled trial.
Lancet. 2011;377:658–666. doi: 10.1016/S0140-6736(11)60101-32131544110.1016/S0140-6736(11)60101-39.HindricksGTaborskyMGliksonMHeinrichUSchumacherBKatzABrachmannJLewalterTGoetteABlockM; IN-TIME Study Group*. Implant-based multiparameter telemonitoring of patients with heart failure (IN-TIME): a randomised controlled trial.
Lancet. 2014;384:583–590. doi: 10.1016/S0140-6736(14)61176-42513197710.1016/S0140-6736(14)61176-410.VarmaNMarroucheNFAguinagaLAlbertCMArbeloEChoiJIVarosyPD
HRS/EHRA/APHRS/LAHRS/ACC/AHA worldwide practical guidance for telehealth and arrhythmia monitoring during and after a pandemic.
Circ Arrhythm Electrophysiol. 2020;13:e009007
doi: 10.1161/CIRCEP.120.0090073269297210.1161/CIRCEP.120.009007PMC748261811.ChungMKEckhardtLLChenLYAhmedHMGopinathannairRJoglarJANoseworthyPAPackQRSandersPTrulockKM; American Heart Association Electrocardiography and Arrhythmias Committee and Exercise, Cardiac Rehabilitation, and Secondary Prevention Committee of the Council on Clinical Cardiology; Council on Arteriosclerosis, Thrombosis and Vascular Biology; Council on Cardiovascular and Stroke Nursing; and Council on Lifestyle and Cardiometabolic Health. Lifestyle and risk factor modification for reduction of atrial fibrillation: a scientific statement from the American Heart Association.
Circulation. 2020;141:e750–e772. doi: 10.1161/CIR.00000000000007483214808610.1161/CIR.0000000000000748

## 2. mHEALTH Technologies

**Figure 1.**
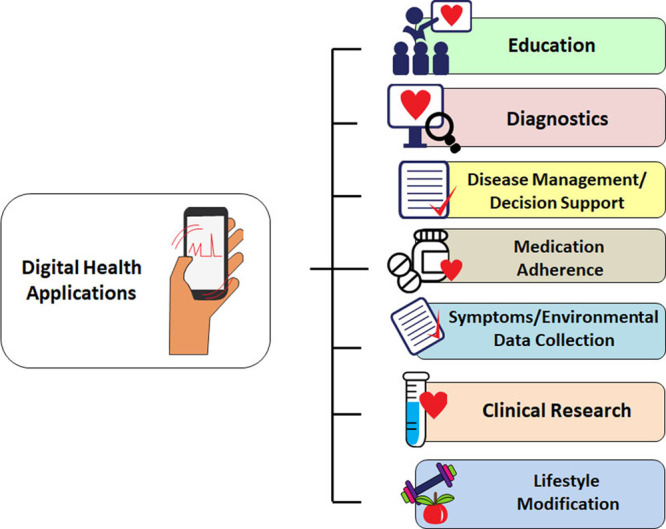
**Application of digital health technologies in arrhythmias (many of these sectors are interconnected).**

Dedicated applications and sensors within or adjunctive to mobile communication devices enable users to monitor, collect, and share physiological and health data. Their applications range from diagnostic, decision support, disease management, evaluation of medication adherence, and for educational and clinical research purposes (Figure 1). They synergize naturally with arrhythmia evaluation and extend management to associated comorbidities and lifestyle.

### Applications to Arrhythmias

- Diagnostic
  - Evaluate patients with symptoms suggestive of arrhythmias.
  - Assess patients’ response to both pharmacological and invasive treatment of arrhythmias.
- Screening
  - Increasing emphasis on AF.

### 2.1. Ambulatory ECG Monitoring

**Figure 2.**
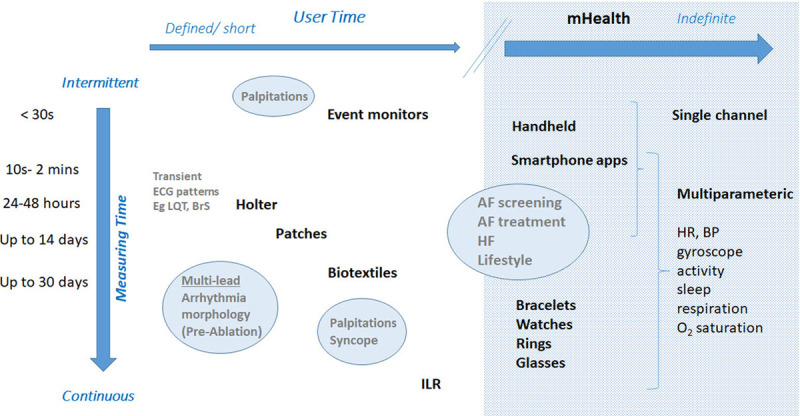
**Mobile health (mHealth) devices for arrhythmia monitoring according to indications.** Traditional wearable monitors are used for defined, short periods of time. Advantages are continuous monitoring and the ability to use multiple leads, which may be important for arrhythmia differentiation. These have been used historically for evaluation of palpitations, syncope, and defining QRS morphology. mHealth extends monitoring time indefinitely, to be defined by the user, and to the possibility of monitoring other parameters simultaneously with the ECG, and linking to machine learning. Typically, mHealth utilizes single-channel ECG or derived heart rate (HR) and discontinuous monitoring. AF indicates atrial fibrillation; BP, blood pressure; BrS, Brugada pattern; HF, heart failure; ILR, implantable loop recorder; and LQT, long QT.

This is the cornerstone diagnostic method, and the choice of technique and time frame depend on whether symptoms (eg, palpitations and syncope) are present and how often they occur (Figure 2). Since the XXI century has become the era of the AF epidemic, the emphasis has shifted to screen for asymptomatic patients at high risk of developing AF or in those with cryptogenic stroke, to enable early treatment with the hope of preventing stroke and other serious complications. Novel tools expand the time window in which information can be gathered and overcome existing limitations with traditional methods, that is, intermittent physical exam or ECG for the detection of a largely asymptomatic arrhythmia.

- Conventional ambulatory ECG devices with continuous or intermittent recording abilities (eg, Holter, mobile cardiac telemetry) increase the diagnostic yield for suspected arrhythmias, but limitations such as inadequate duration of monitoring, insufficient sensitivity or specificity for AF detection, cost, and patient discomfort and inconvenience remain important implementation barriers. Further details on these conventional systems are available in a prior expert consensus statement (Steinberg 2017).^1^
- Implantable loop recorders continuously monitor cardiac rhythm, similar to traditional external loop recorders, but only record an ECG shortly before and after activation by either the patient or by an automated algorithm. The total monitoring period is limited only by battery longevity (≈2–4 years). Newer devices have dedicated algorithms resulting in increased interest in their use for AF detection, especially after cryptogenic stroke. Several approved implantable loop recorder devices are available (Musat 2018, Sakhi 2019, Tomson 2015),^2–4^ and several studies have been performed to evaluate the diagnostic accuracy of these devices (Ciconte 2017, Hindricks 2010, Mittal 2016, Nolker 2016, Sanders 2016).^5–9^ Since implantable loop recorders are invasive and costly, some functions may shift to mHealth.

### 2.2. New mHealth-Based Modalities for Arrhythmia Monitoring

These can be divided into those

- recording ECG tracings (single or multilead, in intermittent or continuous format, of various durations)
- using non-ECG technologies such as pulse photoplethysmography.

mHealth tools permit indefinite monitoring and widen application to a range of conditions and patient populations. There has been rapid development and integration of diagnostic sensors into consumer devices such as smartwatches, fitness bands, and smartphones. However, validation of their notified data (or underlying algorithms) and mechanisms for professional review (as established for CIEDs and mobile cardiac telemetry) are scant, if at all (Section 7). This is open to risks of not detecting significant events or overtreating—for example, false-positive episodes of AF—if not confirmed by expert physicians.

#### 2.2.1. ECG Based

Among these, handheld and patch systems have undergone the most extensive validation.

##### 2.2.1.1. Handheld Devices

Several stand-alone handheld devices operate without additional hardware. These devices with 2 or 3 ECG electrodes on either side generate short, 30-second-to-1-minute, single or multilead ECG recordings. Some of them display ECG tracings on a monitor. Most of these devices are equipped with dedicated automatic algorithms for detection of arrhythmias and usually focus on AF. Recognition of AF is usually based on the analysis of RR interval irregularity. The devices can store ECG tracings, which can be uploaded to a computer for review and are usually available for physicians via web-based platforms. Studies across diverse populations have documented the diagnostic accuracy of handheld devices in detection of AF by short-term rhythm monitoring (Desteghe 2017, Doliwa 2009, Doliwa 2012, Hendrikx 2014, Kaasenbrood 2016, Poulsen 2017, Svennberg 2017, Tavernier 2018, Tieleman 2014, Vaes 2014; Table 2).^10–28^

##### 2.2.1.2. Wearable Patches

Traditional cable/wire-based devices increasingly have been displaced by solutions with electrodes embedded in adhesive patches. Commercially available patches can be worn up to 14 days (Barrett 2014, Turakhia 2013).^29,30^ Unlike adhesive electrodes for lead-based systems, the water-resistant patches are not removed during the monitoring period, leading to greater wear time, more analyzable data, and no lead reversal errors. The cutaneous patch monitors are typically single use and continuously or intermittently record single-lead electrocardiography. Most have an integrated button to mark the timing of symptoms on the recorded rhythm trace. After the monitoring period, the device is returned to the manufacturer for data extraction, analysis by a proprietary algorithm, and further secondary analysis of potential arrhythmias by medical technicians. A diagnostic report is sent to the treating physician. This process may be associated with delays of several weeks.

Although such patches only record a single-lead ECG, a high agreement (*P*<0.001) has been demonstrated compared with multilead Holter monitors for identifying AF events and estimating AF burden (Barrett 2014, Rosenberg 2013).^29,31^ As the patch has no external leads, it is perceived to be more comfortable to wear compared with conventional Holter monitors, with 94% of the patients preferring the patch over the Holter (Barrett 2014).^29^ In addition to the validation studies, the feasibility of 2-week continuous monitoring to identify AF in an at-risk patient population has been examined by Turakhia et al^32^ (2015). It has also been used successfully to determine the prevalence of subclinical AF in the general population (Rooney 2019).^33^

Newer patch-based systems add near-real-time analytics and by transmitting data continuously to the cloud. This may facilitate more rapid data collection and diagnosis. Multiparametric monitoring may be enabled with a patch worn for up to 3 months (Stehlik 2020).^34^

##### 2.2.1.3. Biotextiles

Textile-based systems for ECG monitoring were initially designed to ensure patients’ comfort during daily activities and address the needs of active patients. These vests and elastic bands adapt easily to patients’ movements that is particularly important for those performing physical activities that might be limited by the presence of wires. These biomedical devices capture the electrocardiographic signal via electrodes integrated into the garment that enables noninvasive acquisition of ECG signal up to 30 days. Single/multilead selection (up to full 12 leads) and event activation are available. ECG signals can be stored in memory cards and analyzed afterward, as well as transmitted in real time via Bluetooth to a smartphone (and from there to a cloud-based platform), along with other signals including accelerometer and global positioning signal. Other than ECG, some devices provide data on activity intensity, respiratory function, and sleep quality. Automatic analysis with manual verification is possible. Several systems for ECG monitoring based on electrodes incorporated into garments have been introduced into market. Some of them acquire signal from chest belts. Maintaining power presents a challenge. These systems have been tested in athletes, in patients with cryptogenic stroke, and in those with pacemaker-detected atrial high-rate episodes (Eliot 2019, Eysenck 2019, Fabregat 2014, Feito 2019, Pagola 2018).^35–39^

The wearable cardioverter-defibrillator transmits 2-channel ECG data to an online patient management database allowing for RM of high-risk patients. Recent incorporation of heart sound evaluation that may predict HF decompensation will be tested in a prospective trial (HEARIT-Reg trial [Heart Sounds Registry]; https://www.clinicaltrials.gov; unique identifier: NCT03203629).

##### 2.2.1.4. Smartphone- and Smartwatch-Based Devices

**Figure 3.**
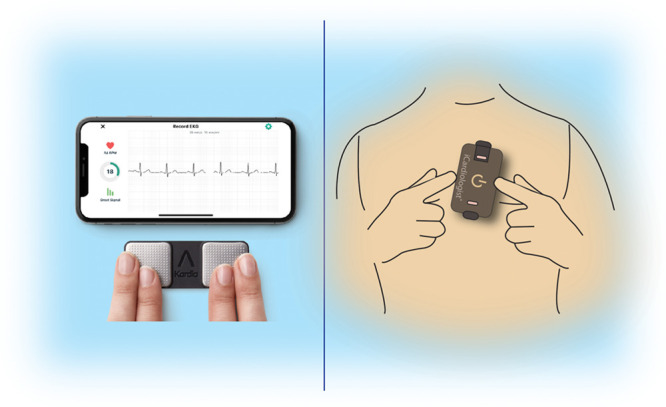
**ECG mobile applications.** **Left**, Fingertip recordings. **Right**, Card pressed to the chest.

More recently, nonwearable solutions coupled to the smartphone have emerged. These devices (eg, Table 2^19–28^; Varma 2020^40^) allow the user to perform a spot-check single-lead ECG strip, usually of up to ≥30 seconds by placing a finger of each hand on the two electrodes, usually located on the phone case or external card (Figure 3). The ECG electrical signal is transmitted wirelessly to a smartphone with an integrated interpretation app. The tracings can be reviewed on the smartphone, electronically stored or transmitted for review by the user’s provider if desired. These have been directed largely to AF.

**Table 2.**
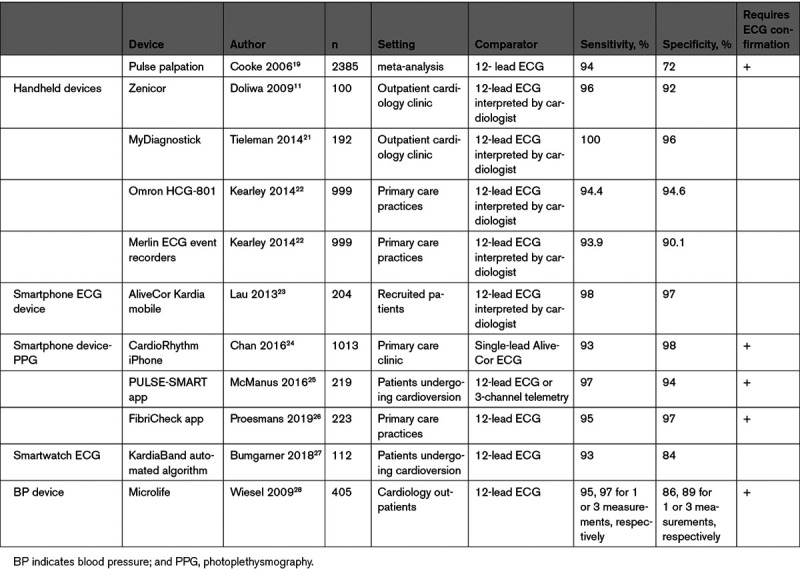
Exemplary Validation Studies for Various Mobile Health Technologies

**Figure 4.**
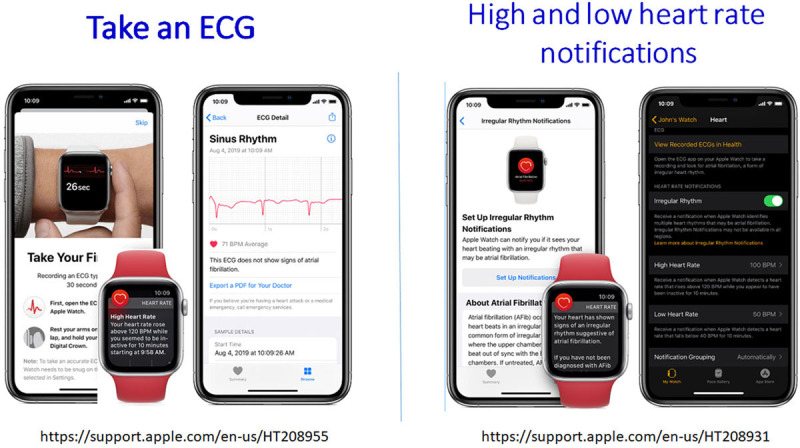
**Apple watch.**

Automated algorithms can label the recording as possible AF on the basis of criteria for the presence and absence of a P wave and the irregularity of the RR interval; normal or sinus rhythm and unreadable when the detector indicates there was too much interference for an adequate recording, whether from too much movement or poor contact between the electrodes and the patient’s skin. Several versions of the AliveCor’s automated algorithms have been evaluated (Chan 2016, Chan 2017, Desteghe 2017, Lowres 2014, Tarakji 2015),^24,41–44^ and the device has been tested as a screening tool in at-risk populations (Halcox 2017, Lowres 2014).^43,45^ In Apple watch, the algorithm is effective when the heart rate (HR) is between 50 and 150 beats per minute; there are no or very few abnormal beats; and the shape, timing, and duration of each beat is considered normal for the patient (Figure 4).

Sensitivity and specificity depend on the software (which can be calibrated to higher sensitivity or higher specificity), the population studied (eg, elderly have more tremor or difficulty in holding the device, leading to more unreadable tracings), and the prevalence of AF in the population. It indicates that use of such device always requires proper evaluation for every intended use case. There is also an accessory band for a smartwatch to allow ECG recording. The single-lead ECG with automatic AF detection is recorded by touching the band’s integrated sensors that transmit data to a watch application. Recently, a new 6-lead case has been developed, allowing for 30-second recording of all 6 limb leads by touching each of the three electrodes. Also the QT interval may be derived from this (https://cardiacrhythmnews.com/kardiamobile-6L-can-be-used-to-measure-qt-duration-in-covid-19-patients/; Chung 2015, Garabelli 2016).^46–48^ Information is limited, however, on how parameters such as QTc measured on a single- (or limited number) lead ECG can reliably substitute for 12-lead ECG information. In one study, QT was underestimated by smartphone single-lead ECG (Koltowski 2019).^49^ Preliminary data indicate the ability for ST monitoring for ischemia (Figure 3; Section 4.1).

Such devices may be used by clinicians as a point-of-care device to obtain an interpretable rhythm strip in place of a 12-lead ECG. In addition, patients may use these devices for ad hoc or routine evaluation of their rhythm in a home environment. The ECG data can be instantaneously transmitted for automated interpretation with the ability of the consumer to request a physician overread for a surcharge.

###### Limitations

- Single-lead devices, particularly when used by an active person who may not be recumbent, relaxed, or still, may lead to substantial electrical or motion artifact. Noise-free tracing my be more difficult for older patients or those with physical limitations (tremor, stroke, etc).
- Although the interpretation algorithms typically have received regulatory oversight, these algorithms can frequently misclassify rhythms, calling sinus rhythm AF and vice versa, which could lead to potential harm without confirmation by a clinician. For example, in a recent study of a consumer ECG device to detect AF, a third of ECGs were unclassifiable by the device but could be classified by experts (Bumgarner 2018).^27^ Therefore, some devices have limitations placed on them for diagnostic assessment. For example, the Apple Watch is unable to assess the ECG for AF if the HR is above 150 or below 50 beats per minute (https://www.apple.com/healthcare/docs/site/Apple_Watch_Arrhythmia_Detection.pdf)^50^ and is cleared by the Food and Drug Administration (FDA) only for use in people without a diagnosis of AF (Figure 4; https://support.apple.com/en-us/HT208931^51^; Section 6).
- For consumer watches, ECG diagnosis is considered a prediagnostic pending medical verification and not designed to be acted on without clinician review.
- ECG classification of other arrhythmias (premature ventricular complexes [PVCs], premature atrial complexes, and ventricular tachycardia) is currently unavailable

#### 2.2.2. Non–ECG Based

##### 2.2.2.1. Photoplethysmography

Consumer devices such as smartphones and smartwatches require accessories and often extra cost for conversion into rhythm-monitoring tools. In contrast, the photoplethysmography technologies allow for the detection of arrhythmias using hardware already present on most consumer devices (smartwatches and fitness bands) through a downloadable application. Photoplethysmography is an optical technique that can be used to detect AF by measuring and analyzing a peripheral pulse waveform. Using a light source and a photodetector, the pulse waveform can be measured by detecting changes in the light intensity, which reflects the tissue blood volume of a skin surface such as the fingertip, earlobe, or face (Conroy 2017, McManus 2013).^52,53^ An automated algorithm can subsequently analyze the generated pulse waveform to detect AF. Photoplethysmography avoids the instability and motion artifacts of ECG sensors and can be passively and opportunistically measured.

###### Applications

This technology has been applied for use with smartphones using the phone’s camera to measure a fingertip pulse waveform. Rapid irregularly conducted AF may produce variable pulse pressures that challenge detection (Choi 2017).^54^ The performance of algorithms interpreting these photoplethysmography signals has been proven to be in high agreement with ECG rhythm strips (McManus 2013, McManus 2016, Proesmans 2019).^53,55,56^ The smartphone-based photoplethysmography applications have been utilized in at-risk population to detect AF and as a screening tool in the general population (Verbrugge 2019^57^; Section 6).

The photoplethysmography technology has also been incorporated in smartwatches to measure HR and rhythm (Dorr 2019, Guo 2019).^58,59^ Some have developed prototypes of a band that includes a single-channel ECG, multiwavelength photoplethysmography, and triaxial accelerometry recording simultaneously at 128 Hz (Nemati 2016),^60^ and others use a deep neural network based on photoplethysmography sensors to detect AF (https://www.mobihealthnews.com/content/study-apple-watch-paired-deep-neural-network-detects-atrial-fibrillation-97-percent-accuracy; https:mrhythmstudy.org).^61,62^ If photoplethysmography or optical sensors and detection algorithms can match the performance of ECG-based rhythm assessment, delivery of AF care may be expected to change substantially and drive a radical departure from relying on an office or ambulatory ECG for ascertainment of AF.

##### 2.2.2.2. Oscillometry

Blood pressure (BP) measurements can be erratic when the pulse is irregular. This characteristic is utilized by automatic oscillometric BP monitors that derive heart rhythm regularity algorithmically (Chen 2017).^63^ Automated BP monitors have been used for opportunistic AF detection. Studies have shown that 6 devices from 2 manufacturers were reliable with sensitivities and specificities >85% (Kane 2016).^64^ These studies suggested that BP devices with embedded algorithms for detecting arrhythmias show promise as screening tools for AF, comparing favorably with manual pulse palpation. Such capability could be added to continuous BP recording devices (Kario 2016).^65^ One device identifies possible AF when at least 2 of 3 consecutive measurements show pulse irregularity. Several studies addressed the diagnostic accuracy (Chan 2017, Chen 2017, Gandolfo 2015, Kearley 2014, Marazzi 2012, Stergiou 2009, Wiesel 2009, Wiesel 2014)^63,66–72^ and the feasibility of this device as a screening tool (Chan 2017, Omboni 2016, Wiesel 2017).^66,72,73^

The following have undergone preliminary study.

##### 2.2.2.3. Mechanocardiography

Mechanocardiography uses accelerometers and gyroscopes to sense the mechanical activity of the heart. The accuracy of this technology to detect AF using a smartphone’s built-in accelerometer and gyroscope sensors was assessed in a proof-of-concept study (Jaakkola 2018).^74^ A smartwatch (Sony Experia) was placed on the chest in supine patients to detect micromovements of the chest. Possibly, carrying this device in a pocket may have utility but is likely to be confounded by movement (eg, walking) artifacts.

##### 2.2.2.4. Contactless Video Plethysmography

Noncontact video monitoring of respiration and HR has been developed <15 years ago (Takano 2007, Verkruysse 2008).^75,76^ In 2014, a pioneering article described the concept of contactless video-based detection of AF (Couderc 2015).^77^ Deep learning of a video of a person’s face can identify AF by examining irregularity of pulsatile facial perfusion (Yan 2018).^78^ It is a monitoring technique extracting the photoplethysmography-like signals from a standard digital RGB video recording of the human skin and specifically of an individual’s face. The videoplethysmographic signal describes the absorption peak of ambient light by the hemoglobin from the facial skin. Several studies have been performed to develop a method that is sensitive enough to detect each cardiac pulse and provide insights into variability on pulse on a beat-to-beat basis. The HealthKam works using HUE color space from video cameras (Dautov 2018, Tsouri 2015)^79,80^ and can easily be integrated to any portable computer device with a camera (smartphone, tablet, etc). By using mobile devices with cameras, the deployment of the technology is easy and scalable since it does not require the use and distribution of any physical devices. Such a system may change the approach to AF screening, which currently is only 1 patient at a time. High-throughput AF detection from multiple patients concurrently using a single digital camera and a pretrained deep convolutional neural network was feasible in a pilot study (Yan 2020).^81^

###### Limitations

One requirement for these technologies is steady focus: thus moving subjects present a challenge. It is important to avoid recording, sending, or communicating any video of the patient, thus protecting privacy and dignity. Video-based technologies in telemedicine have raised a new set of societal and ethical concerns that are being continuously reevaluated such as during the coronavirus disease 2019 (COVID-19) pandemic. Issues regarding privacy, confidentiality, and legal and ethical obligation to treat are crucial factors to be considered when these technologies are deployed at larger scale (Turakhia 2019).^82^

##### 2.2.2.5 Smart Speakers

There are preliminary reports on using commodity smart devices to identify agonal breathing (Chan 2019, Wang 2019).^83,84^ Identification of abnormal HR patterns may be made possible by converting smart speakers into a sonar device with emission of inaudible-frequency sound waves and receiving them to detect motion. These are not in consumer domain but potentially have wide scalability.

## 

References: Section 21.SteinbergJSVarmaNCygankiewiczIAzizPBalsamPBaranchukACantillonDJDilaverisPDubnerSJEl-SherifN
2017 ISHNE-HRS expert consensus statement on ambulatory ECG and external cardiac monitoring/telemetry.
Ann Noninvasive Electrocardiol. 2017;22:e12447.10.1111/anec.12447PMC6931745284806322.MusatDLMilsteinNMittalS
Implantable loop recorders for cryptogenic stroke (plus real-world atrial fibrillation detection rate with implantable loop recorders).
Card Electrophysiol Clin. 2018;10:111–118. doi: 10.1016/j.ccep.2017.11.0112942813210.1016/j.ccep.2017.11.0113.SakhiRTheunsDAMJSzili-TorokTYapSC
Insertable cardiac monitors: current indications and devices.
Expert Rev Med Devices. 2019;16:45–55. doi: 10.1080/17434440.2018.15570463052235010.1080/17434440.2018.15570464.TomsonTTPassmanR
The reveal LINQ insertable cardiac monitor.
Expert Rev Med Devices. 2015;12:7–18. doi: 10.1586/17434440.2014.9530592515497010.1586/17434440.2014.9530595.CiconteGSavianoMGiannelliLCalovicZBaldiMCiaccioCCukoAVitaleRGiacopelliDContiM
Atrial fibrillation detection using a novel three-vector cardiac implantable monitor: the atrial fibrillation detect study.
Europace. 2017;19:1101–1108. doi: 10.1093/europace/euw1812770286510.1093/europace/euw1816.HindricksGPokushalovEUrbanLTaborskyMKuckKHLebedevDRiegerGPürerfellnerH; XPECT Trial Investigators. Performance of a new leadless implantable cardiac monitor in detecting and quantifying atrial fibrillation: results of the XPECT trial.
Circ Arrhythm Electrophysiol. 2010;3:141–147. doi: 10.1161/CIRCEP.109.8778522016016910.1161/CIRCEP.109.8778527.MittalSRogersJSarkarSKoehlerJWarmanENTomsonTTPassmanRS
Real-world performance of an enhanced atrial fibrillation detection algorithm in an insertable cardiac monitor.
Heart Rhythm. 2016;13:1624–1630. doi: 10.1016/j.hrthm.2016.05.0102716569410.1016/j.hrthm.2016.05.0108.NölkerGMayerJBoldtLHSeidlKVAN DrielVMassaTKollumMBrachmannJDenekeTHindricksG
Performance of an implantable cardiac monitor to detect atrial fibrillation: results of the DETECT AF Study.
J Cardiovasc Electrophysiol. 2016;27:1403–1410. doi: 10.1111/jce.130892756511910.1111/jce.130899.SandersPPürerfellnerHPokushalovESarkarSDi BaccoMMausBDekkerLR; Reveal LINQ Usability Investigators. Performance of a new atrial fibrillation detection algorithm in a miniaturized insertable cardiac monitor: results from the Reveal LINQ Usability Study.
Heart Rhythm. 2016;13:1425–1430. doi: 10.1016/j.hrthm.2016.03.0052696129810.1016/j.hrthm.2016.03.00510.DestegheLRaymaekersZLutinMVijgenJDilling-BoerDKoopmanPSchurmansJVanduynhovenPDendalePHeidbuchelH
Performance of handheld electrocardiogram devices to detect atrial fibrillation in a cardiology and geriatric ward setting.
Europace. 2017;19:29–39. doi: 10.1093/europace/euw0252689349610.1093/europace/euw02511.DoliwaPSFrykmanVRosenqvistM
Short-term ECG for out of hospital detection of silent atrial fibrillation episodes.
Scand Cardiovasc J. 2009;43:163–168. doi: 10.1080/140174308025934351909697710.1080/1401743080259343512.HendrikxTRosenqvistMWesterPSandströmHHörnstenR
Intermittent short ECG recording is more effective than 24-hour Holter ECG in detection of arrhythmias.
BMC Cardiovasc Disord. 2014;14:41
doi: 10.1186/1471-2261-14-412469048810.1186/1471-2261-14-41PMC423432513.KaasenbroodFHollanderMRuttenFHGerhardsLJHoesAWTielemanRG
Yield of screening for atrial fibrillation in primary care with a hand-held, single-lead electrocardiogram device during influenza vaccination.
Europace. 2016;18:1514–1520. doi: 10.1093/europace/euv4262685181310.1093/europace/euv426PMC507213514.PoulsenMBBiniciZDominguezHSojaAMKruuseCHornnesAHRasmussenRSOvergaardK
Performance of short ECG recordings twice daily to detect paroxysmal atrial fibrillation in stroke and transient ischemic attack patients.
Int J Stroke. 2017;12:192–196. doi: 10.1177/17474930166698832769431210.1177/174749301666988315.SvennbergEStridhMEngdahlJAl-KhaliliFFribergLFrykmanVRosenqvistM
Safe automatic one-lead electrocardiogram analysis in screening for atrial fibrillation.
Europace. 2017;19:1449–1453. doi: 10.1093/europace/euw2862833957810.1093/europace/euw28616.TavernierRWolfMKatariaVPhlipsTHuysRTaghjiPLouwRHoeyweghenRVVandekerckhoveYKnechtS
Screening for atrial fibrillation in hospitalised geriatric patients.
Heart. 2018;104:588–593. doi: 10.1136/heartjnl-2017-3119812888303210.1136/heartjnl-2017-31198117.TielemanRGPlantingaYRinkesDBartelsGLPosmaJLCatorRHofmanCHoubenRP
Validation and clinical use of a novel diagnostic device for screening of atrial fibrillation.
Europace. 2014;16:1291–1295. doi: 10.1093/europace/euu0572482576610.1093/europace/euu057PMC414960818.VaesBStalpaertSTavernierKThaelsBLapeireDMullensWDegryseJ
The diagnostic accuracy of the MyDiagnostick to detect atrial fibrillation in primary care.
BMC Fam Pract. 2014;15:113
doi: 10.1186/1471-2296-15-1132491360810.1186/1471-2296-15-113PMC406934019.CookeGDoustJSandersS
Is pulse palpation helpful in detecting atrial fibrillation? A systematic review.
J Fam Pract. 2006;55:130–134.1645178020.DoliwaRSobocinskiPAnggårdhREFrykman KullVvon ArbinMWallénHRosenqvistM
Improved screening for silent atrial fibrillation after ischaemic stroke.
Europace. 2012;14:1112–1116. doi: 10.1093/europace/eur4312230808610.1093/europace/eur43121.TielemanRGPlantingaYRinkesDBartelsGLPosmaJLCatorRHofmanCHoubenRP
Validation and clinical use of a novel diagnostic device for screening of atrial fibrillation.
Europace. 2014;16:1291–1295. doi: 10.1093/europace/euu0572482576610.1093/europace/euu057PMC414960822.KearleyKSelwoodMVan den BruelAThompsonMMantDHobbsFRFitzmauriceDHeneghanC
Triage tests for identifying atrial fibrillation in primary care: a diagnostic accuracy study comparing single-lead ECG and modified BP monitors.
BMJ Open. 2014;4:e004565
doi: 10.1136/bmjopen-2013-00456510.1136/bmjopen-2013-004565PMC40254112479325023.LauJKLowresNNeubeckLBriegerDBSyRWGallowayCDAlbertDEFreedmanSB
iPhone ECG application for community screening to detect silent atrial fibrillation: a novel technology to prevent stroke.
Int J Cardiol. 2013;165:193–194. doi: 10.1016/j.ijcard.2013.01.2202346524910.1016/j.ijcard.2013.01.22024.ChanPHWongCKPohYCPunLLeungWWWongYFSiuCW
Diagnostic performance of a smartphone-based photoplethysmographic application for atrial fibrillation screening in a primary care setting.
J Am Heart Assoc. 2016;5:e003428.2744450610.1161/JAHA.116.003428PMC501537925.McManusDDChongJWSoniASaczynskiJSEsaNNapolitanoCDarlingCEBoyerERosenRKFloydKC
PULSE-SMART: pulse-based arrhythmia discrimination using a novel smartphone application.
J Cardiovasc Electrophysiol. 2016;27:51–57. doi: 10.1111/jce.128422639172810.1111/jce.12842PMC476831026.ProesmansTMortelmansCVan HaelstRVerbruggeFVandervoortPVaesB
Mobile phone-based use of the photoplethysmography technique to detect atrial fibrillation in primary care: diagnostic accuracy study of the FibriCheck app.
JMIR Mhealth Uhealth. 2019;7:e12284
doi: 10.2196/122843091665610.2196/12284PMC645682527.BumgarnerJMLambertCTHusseinAACantillonDJBaranowskiBWolskiKLindsayBDWazniOMTarakjiKG
Smartwatch algorithm for automated detection of atrial fibrillation.
J Am Coll Cardiol. 2018;71:2381–2388. doi: 10.1016/j.jacc.2018.03.0032953506510.1016/j.jacc.2018.03.00328.WieselJFitzigLHerschmanYMessineoFC
Detection of atrial fibrillation using a modified microlife blood pressure monitor.
Am J Hypertens. 2009;22:848–852. doi: 10.1038/ajh.2009.981947879310.1038/ajh.2009.9829.BarrettPMKomatireddyRHaaserSTopolSSheardJEncinasJFoughtAJTopolEJ
Comparison of 24-hour Holter monitoring with 14-day novel adhesive patch electrocardiographic monitoring.
Am J Med. 2014;127:95.e11–95.e17. doi: 10.1016/j.amjmed.2013.10.00310.1016/j.amjmed.2013.10.003PMC38821982438410830.TurakhiaMPHoangDDZimetbaumPMillerJDFroelicherVFKumarUNXuXYangFHeidenreichPA
Diagnostic utility of a novel leadless arrhythmia monitoring device.
Am J Cardiol. 2013;112:520–524. doi: 10.1016/j.amjcard.2013.04.0172367298810.1016/j.amjcard.2013.04.01731.RosenbergMASamuelMThosaniAZimetbaumPJ
Use of a noninvasive continuous monitoring device in the management of atrial fibrillation: a pilot study.
Pacing Clin Electrophysiol. 2013;36:328–333. doi: 10.1111/pace.120532324082710.1111/pace.12053PMC361837232.TurakhiaMPUllalAJHoangDDThanCTMillerJDFridayKJPerezMVFreemanJVWangPJHeidenreichPA
Feasibility of extended ambulatory electrocardiogram monitoring to identify silent atrial fibrillation in high-risk patients: the Screening Study for Undiagnosed Atrial Fibrillation (STUDY-AF).
Clin Cardiol. 2015;38:285–292. doi: 10.1002/clc.223872587347610.1002/clc.22387PMC465433033.RooneyMRSolimanEZLutseyPLNorbyFLLoehrLRMosleyTHZhangMGottesmanRFCoreshJFolsomAR
Prevalence and characteristics of subclinical atrial fibrillation in a community-dwelling elderly population: the ARIC Study.
Circ Arrhythm Electrophysiol. 2019;12:e007390
doi: 10.1161/CIRCEP.119.0073903160714810.1161/CIRCEP.119.007390PMC681438734.StehlikJSchmalfussCBozkurtBNativi-NicolauJWohlfahrtPWegerichSRoseKRayRSchofieldRDeswalA
Continuous wearable monitoring analytics predict heart failure hospitalization: the LINK-HF Multicenter Study.
Circ Heart Fail. 2020;13:e006513
doi: 10.1161/CIRCHEARTFAILURE.119.0065133209350610.1161/CIRCHEARTFAILURE.119.00651335.ElliotCAHamlinMJLizamoreCA
Validity and reliability of the hexoskin wearable biometric vest during maximal aerobic power testing in elite cyclists.
J Strength Cond Res. 2019;33:1437–1444. doi: 10.1519/JSC.00000000000020052875953810.1519/JSC.000000000000200536.EysenckWFreemantleNSulkeN
A randomized trial evaluating the accuracy of AF detection by four external ambulatory ECG monitors compared to permanent pacemaker AF detection.
J Interv Card Electrophysiol. 2020;57:361–369. doi: 10.1007/s10840-019-00515-03074136010.1007/s10840-019-00515-037.Fabregat-AndresOMunoz-MachoAAdell-BeltranGIbanez-CatalaXMaciaAFacilaL
Evaluation of a new shirt-based electrocardiogram device for cardiac screening in soccer players: comparative study with treadmill ergospirometry.
Cardiol Res. 2014;5:101–107. doi: 10.14740/cr333w2834870510.14740/cr333wPMC535817038.FeitoYMoriartyTAMangineGMonahanJ
The use of a smart-textile garment during high-intensity functional training: a pilot study.
J Sports Med Phys Fitness. 2019;59:947–954. doi: 10.23736/S0022-4707.18.08689-93002412510.23736/S0022-4707.18.08689-939.PagolaJJuegaJFrancisco-PascualJMoyaASanchisMBustamanteAPenalbaAUseroMCortijoEArenillasJF; CryptoAF Investigators. Yield of atrial fibrillation detection with Textile Wearable Holter from the acute phase of stroke: pilot study of Crypto-AF registry.
Int J Cardiol. 2018;251:45–50. doi: 10.1016/j.ijcard.2017.10.0632910736010.1016/j.ijcard.2017.10.06340.VarmaNMarroucheNFAguinagaLAlbertCMArbeloEChoiJIVarosyPD
HRS/EHRA/APHRS/LAHRS/ACC/AHA worldwide practical guidance for telehealth and arrhythmia monitoring during and after a pandemic.
Circ Arrhythm Electrophysiol. 2020;13:e009007.3269297210.1161/CIRCEP.120.009007PMC748261841.ChanPHWongCKPunLWongYFWongMMChuDWSiuCW
Head-to-head comparison of the AliveCor heart monitor and microlife WatchBP office AFIB for atrial fibrillation screening in a primary care setting.
Circulation. 2017;135:110–112. doi: 10.1161/CIRCULATIONAHA.116.0244392802806610.1161/CIRCULATIONAHA.116.02443942.DestegheLRaymaekersZLutinMVijgenJDilling-BoerDKoopmanPSchurmansJVanduynhovenPDendalePHeidbuchelH
Performance of handheld electrocardiogram devices to detect atrial fibrillation in a cardiology and geriatric ward setting.
Europace. 2017;19:29–39. doi: 10.1093/europace/euw0252689349610.1093/europace/euw02543.LowresNNeubeckLSalkeldGKrassIMcLachlanAJRedfernJBennettAABriffaTBaumanAMartinezC
Feasibility and cost-effectiveness of stroke prevention through community screening for atrial fibrillation using iPhone ECG in pharmacies. The SEARCH-AF study.
Thromb Haemost. 2014;111:1167–1176. doi: 10.1160/TH14-03-02312468708110.1160/TH14-03-023144.TarakjiKGWazniOMCallahanTKanjMHakimAHWolskiKWilkoffBLSalibaWLindsayBD
Using a novel wireless system for monitoring patients after the atrial fibrillation ablation procedure: the iTransmit study.
Heart Rhythm. 2015;12:554–559. doi: 10.1016/j.hrthm.2014.11.0152546085410.1016/j.hrthm.2014.11.01545.HalcoxJPJWarehamKCardewAGilmoreMBarryJPPhillipsCGravenorMB
Assessment of remote heart rhythm sampling using the AliveCor heart monitor to screen for atrial fibrillation: the REHEARSE-AF Study.
Circulation. 2017;136:1784–1794. doi: 10.1161/CIRCULATIONAHA.117.0305832885172910.1161/CIRCULATIONAHA.117.03058346.Cardiac Rhythm News. KardiaMobile 6L can be used to measure QT duration in COVID-19 patients.
Published March 23, 2020. Accessed January 26, 2021. https://cardiacrhythmnews.com/kardiamobile-6l-can-be-used-to-measure-qt-duration-in-covid-19-patients/47.ChungEHGuiseKD
QTC intervals can be assessed with the AliveCor heart monitor in patients on dofetilide for atrial fibrillation.
J Electrocardiol. 2015;48:8–9. doi: 10.1016/j.jelectrocard.2014.10.0052545319410.1016/j.jelectrocard.2014.10.00548.GarabelliPStavrakisSAlbertMKoomsonEParwaniPChohanJSmithLAlbertDXieRXieQ
Comparison of QT interval readings in normal sinus rhythm between a smartphone heart monitor and a 12-lead ECG for healthy volunteers and inpatients receiving sotalol or dofetilide.
J Cardiovasc Electrophysiol. 2016;27:827–832. doi: 10.1111/jce.129762702765310.1111/jce.1297649.KoltowskiLBalsamPGlłowczynskaRRokickiJKPellerMMaksymJBlicharzLMaciejewskiKNiedzielaMOpolskiG
Kardia Mobile applicability in clinical practice: a comparison of Kardia Mobile and standard 12-lead electrocardiogram records in 100 consecutive patients of a tertiary cardiovascular care center [published online January 15, 2019].
Cardiol J. doi: 10.5603/CJ.a2019.000110.5603/CJ.a2019.0001PMC82769943064407950.Using Apple Watch for Arrhythmia Detection.Accessed January 26, 2021. https://www.apple.com/healthcare/docs/site/Apple_Watch_Arrhythmia_Detection.pdf51.Heart Health Notifications on Your Apple Watch.Accessed January 2, 2020. https://support.apple.com/en-us/HT20893152.ConroyTGuzmanJHHallBTsouriGCoudercJP
Detection of atrial fibrillation using an earlobe photoplethysmographic sensor.
Physiol Meas. 2017;38:1906–1918. doi: 10.1088/1361-6579/aa88302883650710.1088/1361-6579/aa883053.McManusDDLeeJMaitasOEsaNPidikitiRCarlucciAHarringtonJMickEChonKH
A novel application for the detection of an irregular pulse using an iPhone 4S in patients with atrial fibrillation.
Heart Rhythm. 2013;10:315–319. doi: 10.1016/j.hrthm.2012.12.0012322068610.1016/j.hrthm.2012.12.001PMC369857054.ChoiAShinH
Photoplethysmography sampling frequency: pilot assessment of how low can we go to analyze pulse rate variability with reliability?
Physiol Meas. 2017;38:586–600. doi: 10.1088/1361-6579/aa5efa2816983610.1088/1361-6579/aa5efa55.McManusDDChongJWSoniASaczynskiJSEsaNNapolitanoCDarlingCEBoyerERosenRKFloydKC
PULSE-SMART: pulse-based arrhythmia discrimination using a novel smartphone application.
J Cardiovasc Electrophysiol. 2016;27:51–57. doi: 10.1111/jce.128422639172810.1111/jce.12842PMC476831056.ProesmansTMortelmansCVan HaelstRVerbruggeFVandervoortPVaesB
Mobile phone-based use of the photoplethysmography technique to detect atrial fibrillation in primary care: diagnostic accuracy study of the FibriCheck app.
JMIR Mhealth Uhealth. 2019;7:e12284
doi: 10.2196/122843091665610.2196/12284PMC645682557.VerbruggeFHProesmansTVijgenJMullensWRivero-AyerzaMVan HerendaelHVandervoortPNuyensD
Atrial fibrillation screening with photo-plethysmography through a smartphone camera.
Europace. 2019;21:1167–1175. doi: 10.1093/europace/euz1193105667810.1093/europace/euz11958.DörrMNohturfftVBrasierNBosshardEDjurdjevicAGrossSRaichleCJRhinispergerMStöckliREcksteinJ
The WATCH AF trial: SmartWATCHes for detection of atrial fibrillation.
JACC Clin Electrophysiol. 2019;5:199–208. doi: 10.1016/j.jacep.2018.10.0063078469110.1016/j.jacep.2018.10.00659.GuoYWangHZhangHLiuTLiangZXiaYYanLXingYShiHLiS; MAFA II Investigators. Mobile photoplethysmographic technology to detect atrial fibrillation.
J Am Coll Cardiol. 2019;74:2365–2375. doi: 10.1016/j.jacc.2019.08.0193148754510.1016/j.jacc.2019.08.01960.NematiSGhassemiMMAmbaiVIsakadzeNLevantsevychOShahACliffordGD
Monitoring and detecting atrial fibrillation using wearable technology.
Annu Int Conf IEEE Eng Med Biol Soc. 2016;2016:3394–3397. doi: 10.1109/EMBC.2016.75914562826903210.1109/EMBC.2016.759145661.Study: Apple Watch paired with deep neural network detects atrial fibrillation with 97 percent accuracy.Accessed January 26, 2021. https://www.mobihealthnews.com/content/study-apple-watch-paired-deep-neural-network-detects-atrial-fibrillation-97-percent-accuracy62.http://www.mrhythmstudy.org/. Accessed January 26, 202163.ChenYLeiLWangJG
Atrial fibrillation screening during automated blood pressure measurement-comment on “Diagnostic accuracy of new algorithm to detect atrial fibrillation in a home blood pressure monitor”.
J Clin Hypertens (Greenwich). 2017;19:1148–1151. doi: 10.1111/jch.130812894261410.1111/jch.13081PMC803128364.KaneSABlakeJRMcArdleFJLangleyPSimsAJ
Opportunistic detection of atrial fibrillation using blood pressure monitors: a systematic review.
Open Heart. 2016;3:e000362
doi: 10.1136/openhrt-2015-0003622709976010.1136/openhrt-2015-000362PMC483630565.KarioK
Evidence and perspectives on the 24-hour management of hypertension: hemodynamic biomarker-initiated ‘anticipation medicine’ for zero cardiovascular event.
Prog Cardiovasc Dis. 2016;59:262–281. doi: 10.1016/j.pcad.2016.04.0012708020210.1016/j.pcad.2016.04.00166.ChanPHWongCKPunLWongYFWongMMChuDWSiuCW
Head-to-head comparison of the AliveCor heart monitor and microlife WatchBP office AFIB for atrial fibrillation screening in a primary care setting.
Circulation. 2017;135:110–112. doi: 10.1161/CIRCULATIONAHA.116.0244392802806610.1161/CIRCULATIONAHA.116.02443967.GandolfoCBalestrinoMBrunoCFinocchiCRealeN
Validation of a simple method for atrial fibrillation screening in patients with stroke.
Neurol Sci. 2015;36:1675–1678. doi: 10.1007/s10072-015-2231-02592607210.1007/s10072-015-2231-068.KearleyKSelwoodMVan den BruelAThompsonMMantDHobbsFRFitzmauriceDHeneghanC
Triage tests for identifying atrial fibrillation in primary care: a diagnostic accuracy study comparing single-lead ECG and modified BP monitors.
BMJ Open. 2014;4:e004565
doi: 10.1136/bmjopen-2013-00456510.1136/bmjopen-2013-004565PMC40254112479325069.MarazziGIellamoFVolterraniMLombardoMPellicciaFRighiDGriecoFCacciottiLIaiaLCaminitiG
Comparison of microlife BP A200 plus and omron M6 blood pressure monitors to detect atrial fibrillation in hypertensive patients.
Adv Ther. 2012;29:64–70. doi: 10.1007/s12325-011-0087-02219890210.1007/s12325-011-0087-070.StergiouGSKarpettasNProtogerouANasothimiouEGKyriakidisM
Diagnostic accuracy of a home blood pressure monitor to detect atrial fibrillation.
J Hum Hypertens. 2009;23:654–8. 10.1038/jhh.2009.51927966110.1038/jhh.2009.571.WieselJFitzigLHerschmanYMessineoFC
Detection of atrial fibrillation using a modified microlife blood pressure monitor.
Am J Hypertens. 2009;22:848–852. doi: 10.1038/ajh.2009.981947879310.1038/ajh.2009.9872.WieselJArbesfeldBSchechterD
Comparison of the Microlife blood pressure monitor with the Omron blood pressure monitor for detecting atrial fibrillation.
Am J Cardiol. 2014;114:1046–1048. doi: 10.1016/j.amjcard.2014.07.0162521254610.1016/j.amjcard.2014.07.01672.OmboniSVerberkWJ
Opportunistic screening of atrial fibrillation by automatic blood pressure measurement in the community.
BMJ Open. 2016;6:e010745
doi: 10.1136/bmjopen-2015-01074510.1136/bmjopen-2015-010745PMC48387272707257173.WieselJSalomoneTJ
Screening for atrial fibrillation in patients ≥65 years using an automatic blood pressure monitor in a skilled nursing facility.
Am J Cardiol. 2017;120:1322–1324. doi: 10.1016/j.amjcard.2017.07.0162882135110.1016/j.amjcard.2017.07.01674.JaakkolaJJaakkolaSLahdenojaOHurnanenTKoivistoTPänkääläMKnuutilaTKiviniemiTOVasankariTAiraksinenKEJ
Mobile phone detection of atrial fibrillation with mechanocardiography: the MODE-AF Study (Mobile Phone Detection of Atrial Fibrillation).
Circulation. 2018;137:1524–1527. doi: 10.1161/CIRCULATIONAHA.117.0328042952683410.1161/CIRCULATIONAHA.117.03280475.TakanoCOhtaY
Heart rate measurement based on a time-lapse image.
Med Eng Phys. 2007;29:853–857. doi: 10.1016/j.medengphy.2006.09.0061707452510.1016/j.medengphy.2006.09.00676.VerkruysseWSvaasandLONelsonJS
Remote plethysmographic imaging using ambient light.
Opt Express. 2008;16:21434–21445. doi: 10.1364/oe.16.0214341910457310.1364/oe.16.021434PMC271785277.CoudercJPKyalSMesthaLKXuBPetersonDRXiaXHallB
Detection of atrial fibrillation using contactless facial video monitoring.
Heart Rhythm. 2015;12:195–201. doi: 10.1016/j.hrthm.2014.08.0352517948810.1016/j.hrthm.2014.08.03578.YanBPLaiWHSChanCKYChanSCHChanLHLamKMLauHWNgCMTaiLYYipKW
Contact-free screening of atrial fibrillation by a smartphone using facial pulsatile photoplethysmographic signals.
J Am Heart Assoc. 2018;7:e008585.2962259210.1161/JAHA.118.008585PMC601541479.DautovRSavurCTsouriG
On the effect of face detection on heart rate estimation in videoplethysmography.
In: 2018 IEEE Western New York Image and Signal Processing Workshop (WNYISPW), Rochester, NY, October 5, 2018Accessed January 26, 2021. 10.1109/wnyipw.2018.857643980.TsouriGRLiZ
On the benefits of alternative color spaces for noncontact heart rate measurements using standard red-green-blue cameras.
J Biomed Opt. 2015;20:048002
doi: 10.1117/1.JBO.20.4.0480022587562810.1117/1.JBO.20.4.04800281.YanBPLaiWHSChanCKYAuACKFreedmanBPohYCPohMZ
High-throughput, contact-free detection of atrial fibrillation from video with deep learning.
JAMA Cardiol. 2020;5:105–107. doi: 10.1001/jamacardio.2019.40043177446110.1001/jamacardio.2019.4004PMC690212382.TurakhiaMP
Diagnosing with a camera from a distance-proceed cautiously and responsibly.
JAMA Cardiol. 2020;5:107
doi: 10.1001/jamacardio.2019.457210.1001/jamacardio.2019.45723177444883.ChanJReaTGollakotaSSunshineJE
Contactless cardiac arrest detection using smart devices.
NPJ Digit Med. 2019;2:52
doi: 10.1038/s41746-019-0128-73130439810.1038/s41746-019-0128-7PMC658458284.WangASunshineJEGollakotaS
Contactless infant monitoring using white noise.
Accessed January 26, 2021. doi: 10.1145/3300061.3345453. https://homes.cs.washington.edu/~gshyam/Papers/whitenoise.pdf

## 3. mHEALTH Applications for Arrhythmias

Typically, most patients with palpitations and dizziness are evaluated using the various technologies reviewed in Section 2.1 (Steinberg 2017).^1^ Devices capable of recording at least 1 ECG lead allow the interpreting clinician to distinguish between wide- and narrow-complex rhythms, bradycardia, and tachycardia and thus distinguish between the various causative rhythms. Smart devices may be useful in pediatric patients (Gropler 2018).^2^

### 3.1. Atrial Fibrillation

**Figure 5.**
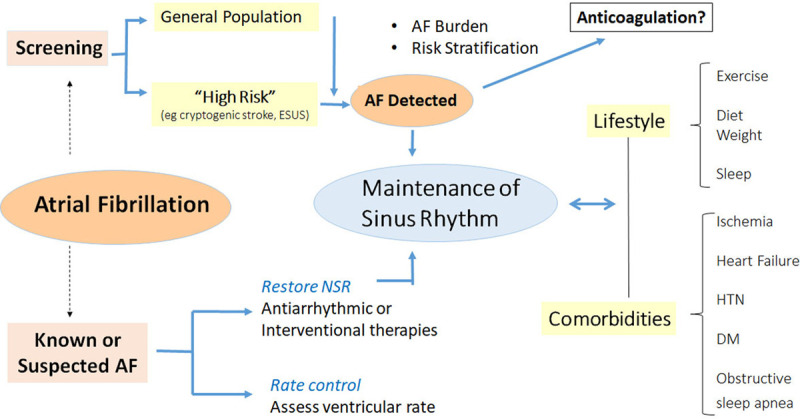
**Mobile health and atrial fibrillation (AF).** Applications include screening for AF in general or high-risk populations, managing comorbidities and lifestyles important for prevention and control (see Section 4), as well as managing treatment of known AF. DM indicates diabetes; ESUS, embolic stroke of unknown source; HTN, hypertension; and NSR, normal sinus rhythm.

The disease is often intermittent and asymptomatic, which may delay diagnosis (McCabe 2016, Strickberger 2005, Verma 2013),^3–5^ lead to incorrect estimation of AF burden (Boriani 2015, Garimella 2015),^6,7^ and pose management challenges to health care services, thereby exposing the patient to the consequences of untreated AF. New digital health and sensor technologies have the potential for early identification of AF, opening up opportunities for screening, which then can be tied to evidence-based management. These may be directed to several broad groups: for screening the general population or managing the already diagnosed, for following responses to treatment, and increasingly to managing comorbidities and lifestyle modification (Section 4; Figure 5). mHealth mechanisms may facilitate understanding the relation between AF burden, its progression, and cardiovascular risk (Wong 2018).^8^

#### 3.1.1. Undiagnosed AF Identification

Classical epidemiological data point to the notion that early identification of AF has the potential to improve morbidity and possibly mortality. (1) AF is associated with a 5-fold increased risk of stroke (Wolf 1991)^9^ and doubled mortality (Kirchhof 2016)^10^; (2) the prevalence of undiagnosed AF is at least 1.5% for patients >65 years of age (Orchard 2018)^11^; (3) in about a quarter of all AF-related strokes, stroke is the first manifestation of the arrhythmia (Friberg 2014)^12^ while other patients with AF present first with congestive HF; (4) stroke risk is independent of symptoms (Xiong 2015)^13^; (5) diagnosis often requires repeated or prolonged ECG monitoring; and (6) oral anticoagulants (OACs) are highly effective in reducing the risk of cardioembolic stroke, mortality, and possibly dementia in the setting of AF (Ding 2018, Friberg 2018).^14,15^

AF identification depends on factors having to do with the arrhythmia itself, that is, the combination of AF prevalence and density (Charitos 2012),^16^ and factors associated with detection such as the frequency and duration of monitoring and diagnostic test performance (Ramkumar 2018).^17^ Several studies including patients with variable stroke risk factors have used mHealth technologies to identify undiagnosed AF (Table 2^18–27^; Table 3^20,28–44^), but these may require gold standard ECG confirmation.

**Table 3.**
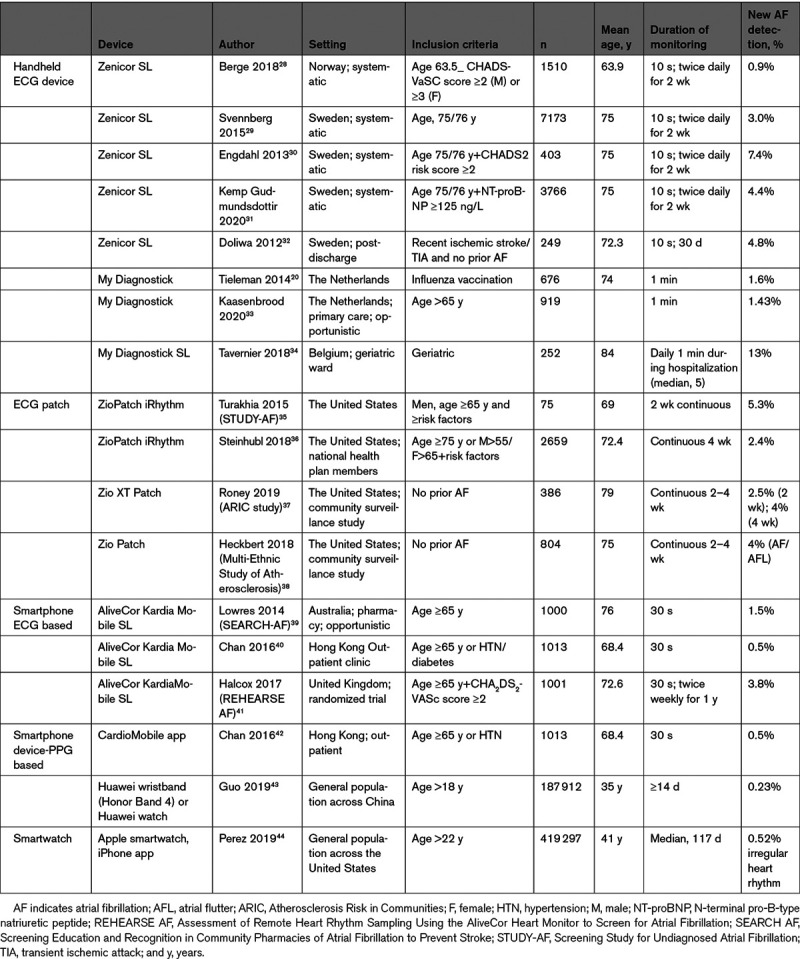
Selected Screening Studies for Atrial Fibrillation Using Newer Technologies

##### Accuracy

The positive predictive value of an AF event will differ according to pretest probability of AF in a given population (eg, those with an established diagnosis or ≥1 risk factors). This is especially relevant to healthy consumers. Many technologies to identify AF are readily available directly to those without defined disease and are not deployed as individual or public health interventions. Rather, consumers who possess these technologies, such as smartwatches or smartphone-connected ECG recorders, opt into the use of these technologies. Therefore, consumer-driven AF identification is not the same as health care–initiated AF screening. AF identification by these devices requires confirmation, since these AF screening tools have variable specificity (Table 2^18–27^), raising the potential of a high false-positive rate in a low-prevalence population, and risks of unnecessary treatment.

There have been almost 500 studies assessing accuracy of mHealth devices for AF detection, as described in recent systematic reviews (Giebel 2019, Lowres 2019, O’Sullivan 2020).^45–47^ Their capabilities varied according to technologies utilized, settings, and study populations. Two large-scale screening trials were reported recently (Section 6).

##### Outcomes

No large outcome trial of screen detected AF and hard end points of stroke and death has been conducted as yet.

Although an incidental diagnosis of AF seems to be associated with increased risk of stroke and protection by OAC therapy (Freedman 2016, Martinez 2014, Tsivgoulis 2019),^48–50^ clinical trials to determine any benefit for opportunistically detected AF have not yet been completed but are underway (Gudmundsdottir 2020, Steinhubl 2018, Svennberg 2015; Heartline Study: www.heartline.com).^29,31,36,51^ This effort addresses the concern that AF detected by screening may identify inherently lower risk patients so that the efficacy of anticoagulation (and its risk/benefit ratio) requires recalibration. This is necessary before issuance of any recommendations (currently, no consensus exists yet on how to treat these arrhythmias, even in those with high CHA_2_DS_2_-VASc scores).

The European and American guidelines do recommend opportunistic screening for early identification of undiagnosed AF in patients aged ≥65 years (Freedman 2017, January 2019, Kirchhof 2016).^10,52,53^ On the contrary, the US Preventative Services Task Force has presently given an insufficient recommendation for systematic screening for AF with electrocardiograms (Jonas 2018).^54^

#### 3.1.2. Targeted Identification in High-Risk Individuals

##### Cryptogenic Stroke/TIA

Up to one-third of ischemic strokes are attributed to AF-mediated embolism to the brain (Hannon 2010).^55^ Further, the risk of recurrent thromboembolism is high if AF is left undetected and untreated (Furie 2012, Kolominsky-Rabas 2001).^56,57^ Hence, prolonged monitoring for AF poststroke has been recommended in recent guidelines (January 2019, Kirchhof 2016, Schnabel 2019).^10,53,58^ Detection of AF poststroke depends not only on the monitoring device used and the duration of the monitoring period but also on stroke type and patient selection; thus, the results of AF detection have been heterogenous (Kishore 2014, Sanna 2014, Zungsontiporn 2018).^59–61^ A meta-analysis showed that a stepwise approach to AF detection in poststroke patients led to AF detection in 23.7% of patients (Sposato 2015)^62^ while a combined analysis of 2 randomized and 2 observational studies showed a 55% reduction in recurrent stroke following prolonged cardiac monitoring (Tsivgoulis 2019).^50^ However, the optimal AF duration threshold for initiating anticoagulation is currently unknown and may be lower in a poststroke population compared with those with fewer cardiovascular risk factors (Kaplan 2019).^63^

The risk of undiagnosed AF and other sources of thrombi has been considered high in embolic strokes of unknown source (ESUS), prompting studies that evaluated whether empirical novel oral anticoagulant therapy is more effective than antiplatelet therapy without a requirement of AF detection. Two of these studies, NAVIGATE ESUS (Rivaroxaban Versus Aspirin in Secondary Prevention of Stroke and Prevention of Systemic Embolism in Patients With Recent Embolic Stroke of Undetermined Source; Hart 2018)^64^ and RESPECT-ESUS (Dabigatran Etexilate for Secondary Stroke Prevention in Patients With Embolic Stroke of Undetermined Source; Diener 2018)^65^ have not shown a reduction in recurrent stroke in patients receiving novel oral anticoagulants. It should be emphasized that the mere detection of AF after ESUS is not necessarily proof of positive causation. A third study is ongoing, including patients with suggested atrial myopathy (enlarged atria, increased levels of NT-proBNP [N-terminal pro-B-type natriuretic peptide], or enlarged P waves; Kamel 2019).^66^

These findings underscore the need for AF detection before initiation of OAC therapy in patients with cryptogenic stroke, ESUS, or ischemic stroke of known origin, and mHealth devices can ease the process of detection (Zungsontiporn 2018).^61^ The threshold of AF burden may differ in patients who have had a suspected cardioembolic event and those who have not (Kaplan 2019).^63^

##### Other High-Risk Individuals

The key to making AF identification feasible, efficient, and clinically valuable is the selection of patients with an increased likelihood of harboring undiagnosed AF, rather than general screening in unselected populations. mHealth ECG recorders can facilitate frequent brief (eg, 30 seconds) recordings over prolonged periods of time by the ubiquity of devices (including smartphone-based apps or watches). These devices are particularly well suited to capture intermittent or nonpersistent arrhythmias; however, it is likely that frequent sampling would be necessary to capture infrequent paroxysmal AF, and even daily snapshot ECG monitoring may miss half of AF episodes (Charitos 2012, Yano 2016).^16,67^ AF burden, increasingly recognized as a powerful independent predictor of stroke (Chen 2018),^68^ though accurately measured by implanted devices (Varma 2005),^69^ cannot be readily calculated from intermittent ECG data. The use of smartwatches with passive intermittent surveillance using photoplethysmography monitoring plus ECG confirmation may be a more effective screening tool and is currently being evaluated (Heartline Study).

Formal screening with mHealth ECG recordings has yielded meaningful incidences of newly diagnosed AF, statistically greater than if diagnosis relied only on the office ECG (Table 3).^20,28–44^ The yield generally is enhanced by the presence of risk factors such as older age and higher CHA_2_DS_2_-VASc scores. Several studies (Chan 2017, Chan 2017a, Proietti 2016)^70,71^ screened untargeted populations, and all yielded new AF diagnoses at a rate under 1%. By focusing on older patients (75–76 years of age) at greater risk, Swedish studies identified new AF in 3% of study participants and up to 7.4% when additional risk factors beyond age were required (Engdahl 2013, Gudmundsdottir 2019, Svennberg 2015).^29–31^ Lowres et al in a patient-level meta-analysis found that new AF detection rate increased progressively with age from 0.34% for <60 years to 2.73% for ≥85 years. Importantly, the number of subjects needed to screen to discover AF meeting indications for anticoagulation was 1089 for subjects <60 but 83 for subjects ≥65 years of age.

#### 3.1.3. Diagnostics in People With Established AF

mHealth has important implications for the care of those already diagnosed with AF. Several key characteristics of AF can be measured with long-term continuous or near-continuous monitoring, and the information gained may provide valuable information for patient management.

Furthermore, while several studies succeeded in establishing the sensitivity and specificity of novel devices for the detection of AF, no study to date has yet evaluated the utility of an mHealth intervention in affecting clinical outcomes. The iHEART study (iPhone Helping Evaluate Atrial Fibrillation Rhythm Through Technology) is a single-center, prospective, randomized controlled trial, and the Heartline study seeks to accomplish this goal (Caceres 2020, Hickey 2016; https://www.heartline.com).^51,72,73^

#### 3.1.4. AF Therapy

##### AF Burden

Current guidelines for anticoagulation are based principally on the presence of risk factors and a diagnosis of clinical AF, regardless of AF duration, symptomatology, or burden (January 2019).^53^ This applies even if the AF has been quiescent for long periods or eliminated altogether as the result of rhythm control interventions including antiarrhythmic drugs, ablation, or risk factor modification (January 2019).^53^ However, there is increasing recognition that AF burden matters, for example, paroxysmal events have less thromboembolic risk than persistent AF (Chen 2018).^68^ This understanding has been extended during continuous monitoring from CIEDs, which depict AF with high granularity, and first advanced the metrics of AF days and burden in terms of cumulative load (hours/day) and concentration (density of AF days; Varma 2005).^69^ This measure is likely to be important for understanding mHealth-discovered AF.

##### Cardiovascular Implantable Electronic Devices

AF burden can be characterized as %/time monitored, longest duration, and density. Retrieved data provide an insight into the natural history and associated sequelae (Healey 2012, Kaplan 2019, Van Gelder 2017, Varma 2005).^63,69,74,75^ This led to oral anticoagulation intervention trials to determine the ability to reduce stroke on the basis of AF duration (Lopes 2017, Martin 2015).^76,77^ These suggest that a threshold exists below which the risk of thromboembolic stroke is low and risk-benefit ratio may not justify chronic administration of OACs. For instance, CIED data indicate that short subclinical AF events have lesser risk than more prolonged (and, therefore, more likely to be symptomatic) events (Al-Turki 2019).^78^ Device-detected, subclinical, atrial, high-rate episodes lasting 6 minutes to 24 hours are associated with increased stroke risk, but the absolute risk is considerably lower than expected based on risk factors alone (Glotzer 2003, Healey 2012, van Gelder 2017).^74,75,79^ Whether these require anticoagulation in high-risk individuals is the subject of ongoing studies (Kirchhof 2017, Lopes 2017, van Gelder 2017).^75,76,80^ Importantly, very short AF episodes (episodes in which both the onset and offset of AT/AF were present within a single electrogram recording) were not associated with adverse outcomes (Swiryn 2016),^81^ which may be important for mHealth monitoring.

##### mHealth

AF detection using digital health tools offers further insights in patients without indication for implantable devices. mHealth extends AF screening to younger patients without cardiovascular disease, and thromboembolic potential may be low. Those with high AF burden (defined by ≥11.4%; mean duration, 11.7 hours) detected on a 14-day patch monitor had an increased thromboembolic event rate compared with those with lower AF burdens (Go 2018).^82^ There remains significant treatment variation in use of OAC, especially for device-detected AF (Perino 2019).^83^ This may be due to a large clinical uncertainty regarding the optimal cut point, even though observational data indicate that OAC is associated with a decreased risk of stroke for episodes >24 hours and possibly for episodes 6 to 24 hours (Perino 2019).^83^

Currently, there are no prospectively validated cut points or risk models that incorporate AF burden into decision-making for stroke prevention therapies.

###### Key Knowledge Gap.

Identify characteristics (duration, episode number/density) and risk factors that justify anticoagulation for mHealth-detected AF.

##### Rhythm and Rate Control

###### Rhythm.

While we await data on OAC treatment for mHealth-detected AF, the finding of the arrhythmia should initiate mHealth monitoring of NSR retention, QT intervals, important for those on some antiarrhythmic drugs (Garabelli 2016),^84^ and discussion of cardiovascular risk factor modification and lifestyle changes, since AF coexists with comorbidities that may influence its occurrence and natural history (Section 4). Thus, alcohol reduction, treatment of OSA, moderate exercise, and weight loss have been shown to reduce AF burden (Congrete 2018, Kanagala 2003, Pathak 2015, Voskoboinik 2020).^85–88^

###### Rate.

While the primary goal of rate control is to minimize AF-related symptoms, prolonged tachycardia can result in effort intolerance or tachycardia-mediated cardiomyopathy while excessively low heart rate targets may increase the risk of bradyarrhythmias that result in symptoms and device implantation. The European Society of Cardiology recommends lenient resting HR targets (<100–110), whereas the American College of Cardiology/American Heart Association/HRS guidelines recommend a target rate of <80 beats per minute. Often these targets are tailored to the individual patient based on symptoms and presence or propensity for HF. mHealth technologies can be used to assess ventricular rates during AF over long time periods and evaluate the effects of rate-control therapies (January 2019, Kirchhof 2016).^10,53^

### 3.2. Sudden Cardiac Death

#### 

##### Ventricular Arrhythmias

The use of mHealth technology to diagnose ventricular arrhythmias lags behind its application to AF (Sections 3.1 and 4.1). Detection of symptomatic ventricular tachycardia has been reported using the AliveCor cardiac monitor (AliveCor, San Francisco, CA) and SmartWatch (Ringwald 2019, Waks 2015).^89,90^ Sophisticated automated analysis of a 2-minute photoplethysmography recording by the camera of a commercially available smartphone (iPhone 4S; Apple) can distinguish between AF, premature atrial complexes, and PVCs from sinus rhythm, with a sensitivity of 0.733 and specificity of 0.976 for PVCs (Chong 2015, McManus 2016).^24,91^ PVCs may challenge to photoplethysmography-based systems, as many PVCs are nonperfusing (Billet 2019).^92^ An ECG tracing is, therefore, essential to facilitate rhythm diagnosis and avoid misclassification of slow photoplethysmography pulse rates (bradysphygmia) simply as bradycardia.

##### Syncope

Syncope presents unique challenges for mHealth applications. While prolonged ambulatory monitoring using medical-grade devices (wearable and implantable) has been the mainstay of cardiac rhythm diagnosis during episodes of syncope, user-activated systems must either be activated by the patient during prodromal symptoms (if present and time permits) in anticipation of syncope or else incorporate loop recording to allow postsyncope activation (Steinberg 2017).^1^ This capability is not incorporated in currently popular consumer-grade wearable devices. However, a randomized controlled trial of AliveCor versus usual care in participants presenting with palpitations or presyncope showed a faster and increased rate of detection of symptomatic arrhythmias in the intervention group, suggesting that at least in presyncope, patient-activated rhythm detection using a commercially available mHealth device is productive (Reed 2019).^93^ Rhythms reported by devices that rely on HRs will likely require validation with a medical-grade system to provide an ECG tracing during an event to allow determination of the causative rhythm.

There is a significant overlap between transient loss of consciousness and mechanical falls due to orthostatic intolerance, neurological, or orthopedic problems. This is particularly disabling in elderly subjects and often unwitnessed (Davis 2010, Heinrich 2010).^94,95^ Mobile applications that combine analysis of HR monitoring together with fall detection, global positioning signal positioning, video recording with display of patients’ surroundings, and the capability to send alerts either triggered by patients in case of symptoms or automatically in case of detected falls may be useful.

##### Cardiac Arrest

The detection and response to sudden cardiac arrest is an area where mHealth applications may prove lifesaving. As rapid treatment for cardiac arrest has consistently been associated with improved survival, preemptive identification of at-risk people, detection of cardiac arrests, alerting of nearby lay and professional first responders, and coaching or quality assurance in the performance of cardiopulmonary resuscitation (CPR) are ideally suited to the mHealth paradigm in societies where mobile smartphones are ubiquitous.

###### 

####### Prediction

It is possible that mHealth devices that continuously monitor heart rhythm and other physiological data may be able to better predict impending sudden cardiac arrest, even using measures that have not shown sufficient specificity or sensitivity when measured intermittently, such as HR variability (Lee 2016).^96^ However, such continuous monitoring is present already in CIEDs and has not yet proven to be sufficiently predictive to be clinically useful (Au-Yeung 2018).^97^ Therefore, the prediction of sudden cardiac arrest by mHealth devices, while a tantalizing prospect, remains to be realized.

####### Notification and Reaction

Once cardiac arrest occurs, rapid identification is essential to trigger a response by emergency responders. Wearable devices that combine physiological monitoring, global positioning signal, and a method of communication with emergency services such as cellular service are well positioned to provide almost instantaneous alert, as well as location information (Kwon 2019, Praveen 2019).^98,99^ An early device using a piezoelectric sensor to detect the pulse was capable of transmitting an alert to emergency medical system or other responders when a pulse was not detected and the watch (and thus the wearer) was still (Rickard 2011).^100^ Preliminary reports indicate that smart speakers in commodity smart devices may be able to identify agonal breath patterns for sudden cardiac death detection (Chan 2019).^101^ Widespread diffusion of such technology to patients at elevated risk of sudden cardiac arrest will be necessary before any potential benefits can be tested.

The ubiquity of mobile phones in society leads to more rapid notification of emergency services and the possibility of a dispatcher gathering information from a bystander at the patient’s side and delivering instructions on care, such as CPR. This was associated with improved outcomes for a variety of emergencies (Wu 2012).^102^ Notification of lay first responders in the vicinity of a cardiac arrest is also feasible with current technology. A blinded, randomized trial conducted in Stockholm, Sweden, demonstrated that such a system improved the rate of bystander CPR (Ringh 2015).^103^ However, almost 10 000 volunteers were recruited over ≈18 months, during which 667 activations occurred, emphasizing the large resources needed and the low rate of utilization of trained volunteers, even when alerted by mobile phone.

Whether a trained or novice bystander responds, mobile devices may be further useful to provide voice (or video) instructions from a dispatcher or from the device itself. Studies of prerecorded audio, live video, and animation-based instruction have shown improvements in some aspects of CPR delivery and automated external defibrillator (AED) use, although technology continues to evolve (Bolle 2009, Choa 2008, Merchant 2010, You 2008).^104–107^ One limitation is that as such apps are unregulated, many do not convey current basic life support algorithms and may have poor usability (Kalz 2014).^108^ In addition, delay in commencing CPR and in calling emergency services due to distraction of the rescuer by using an app is a concern (Paal 2012).^109^

AED use in cardiac arrest is associated with improved survival, but AED use remains low (Weinsfeldt 2010).^110^ Mobile devices have the potential to increase this by assisting with the retrieval and use of AEDs. Multiple apps have been created to locate AEDs in the vicinity of the user, although with perhaps surprisingly mixed results in simulations (Sakai 2011, Hatakeyama 2018, Neves Briard 2019).^111–113^ Barriers include the accuracy of AED location databases, size of the user base, app interface, and the availability of multiple apps instead of a single validated regional, national, or international standard. An emerging approach to circumvent these limitations is the dispatch of an AED via a drone to the location of the cardiac arrest, which is expected to reduce time to defibrillation, especially in rural areas (Boutilier 2017).^114^ Feasibility has been demonstrated (Claesson 2017).^115^

####### Clinical Trial

The complete chain from activation of citizen responders was tested in the Heartrunner trial (Andelius 2020)^116^ in a region of almost 2 million inhabitants. Results showed that citizen responders arrived before emergency services in 42% of out-of-hospital cardiac arrests, accompanied by a 3-fold increase in bystander defibrillation with a trend to improved 30-day survival. Results were more pronounced when emergency arrival times were longer, for example, in rural areas.

## 

References: Section 31.SteinbergJSVarmaNCygankiewiczIAzizPBalsamPBaranchukACantillonDJDilaverisPDubnerSJEl-SherifN
2017 ISHNE-HRS expert consensus statement on ambulatory ECG and external cardiac monitoring/telemetry.
Ann Noninvasive Electrocardiol. 2017;22:e12447.10.1111/anec.12447PMC6931745284806322.GroplerMRFDalalASVan HareGFSilvaJNA
Can smartphone wireless ECGs be used to accurately assess ECG intervals in pediatrics? A comparison of mobile health monitoring to standard 12-lead ECG.
PLoS One. 2018;13:e0204403
doi: 10.1371/journal.pone.02044033026099610.1371/journal.pone.0204403PMC61600473.McCabePJChamberlainAMRhudyLDeVonHA
Symptom representation and treatment-seeking prior to diagnosis of atrial fibrillation.
West J Nurs Res. 2016;38:200–215. doi: 10.1177/01939459155703682569417710.1177/01939459155703684.StrickbergerSAIpJSaksenaSCurryKBahnsonTDZieglerPD
Relationship between atrial tachyarrhythmias and symptoms.
Heart Rhythm. 2005;2:125–131. doi: 10.1016/j.hrthm.2004.10.0421585128310.1016/j.hrthm.2004.10.0425.VermaAChampagneJSappJEssebagVNovakPSkanesAMorilloCAKhaykinYBirnieD
Discerning the incidence of symptomatic and asymptomatic episodes of atrial fibrillation before and after catheter ablation (DISCERN AF): a prospective, multicenter study.
JAMA Intern Med. 2013;173:149–156. doi: 10.1001/jamainternmed.2013.15612326659710.1001/jamainternmed.2013.15616.BorianiGLarocheCDiembergerIFantecchiEPopescuMIRasmussenLHSinagraGPetrescuLTavazziLMaggioniAP
Asymptomatic atrial fibrillation: clinical correlates, management, and outcomes in the EORP-AF Pilot General Registry.
Am J Med. 2015;128:509–18.e2. doi: 10.1016/j.amjmed.2014.11.0262553442310.1016/j.amjmed.2014.11.0267.GarimellaRSChungEHMounseyJPSchwartzJDPursellIGehiAK
Accuracy of patient perception of their prevailing rhythm: a comparative analysis of monitor data and questionnaire responses in patients with atrial fibrillation.
Heart Rhythm. 2015;12:658–665. doi: 10.1016/j.hrthm.2015.01.0122559592610.1016/j.hrthm.2015.01.0128.WongJAConenDVan GelderICMcIntyreWFCrijnsHJWangJGoldMRHohnloserSHLauCPCapucciA
Progression of device-detected subclinical atrial fibrillation and the risk of heart failure.
J Am Coll Cardiol. 2018;71:2603–2611. doi: 10.1016/j.jacc.2018.03.5192988011910.1016/j.jacc.2018.03.5199.WolfPAAbbottRDKannelWB
Atrial fibrillation as an independent risk factor for stroke: the Framingham Study.
Stroke. 1991;22:983–988. doi: 10.1161/01.str.22.8.983186676510.1161/01.str.22.8.98310.KirchhofPBenussiSKotechaDAhlssonAAtarDCasadeiBCastellaMDienerHCHeidbuchelHHendriksJ
2016 ESC Guidelines for the management of atrial fibrillation developed in collaboration with EACTS: the Task Force for the management of atrial fibrillation of the European Society of Cardiology (ESC) Developed with the special contribution of the European Heart Rhythm Association (EHRA) of the ESC. Endorsed by the European Stroke Organisation (ESO).
Eur Heart J. 2016;37:2893–2962.2756740811.OrchardJJNeubeckLFreedmanBWebsterRPatelAGallagherRLiJHespeCMFergusonCZwarN
Atrial Fibrillation Screen, Management and Guideline Recommended Therapy (AF SMART II) in the rural primary care setting: an implementation study protocol.
BMJ Open. 2018;8:e023130
doi: 10.1136/bmjopen-2018-02313010.1136/bmjopen-2018-023130PMC62527583038544412.FribergLRosenqvistMLindgrenATeréntANorrvingBAsplundK
High prevalence of atrial fibrillation among patients with ischemic stroke.
Stroke. 2014;45:2599–2605. doi: 10.1161/STROKEAHA.114.0060702503471310.1161/STROKEAHA.114.00607013.XiongQProiettiMSenooKLipGY
Asymptomatic versus symptomatic atrial fibrillation: a systematic review of age/gender differences and cardiovascular outcomes.
Int J Cardiol. 2015;191:172–177. doi: 10.1016/j.ijcard.2015.05.0112597419310.1016/j.ijcard.2015.05.01114.DingMQiuC
Atrial fibrillation, cognitive decline, and dementia: an epidemiologic review.
Curr Epidemiol Rep. 2018;5:252–261. doi: 10.1007/s40471-018-0159-73014804110.1007/s40471-018-0159-7PMC609685415.FribergLRosenqvistM
Less dementia with oral anticoagulation in atrial fibrillation.
Eur Heart J. 2018;39:453–460. doi: 10.1093/eurheartj/ehx5792907784910.1093/eurheartj/ehx57916.CharitosEIStierleUZieglerPDBaldewigMRobinsonDRSieversHHHankeT
A comprehensive evaluation of rhythm monitoring strategies for the detection of atrial fibrillation recurrence: insights from 647 continuously monitored patients and implications for monitoring after therapeutic interventions.
Circulation. 2012;126:806–814. doi: 10.1161/CIRCULATIONAHA.112.0980792282443410.1161/CIRCULATIONAHA.112.09807917.RamkumarSNerlekarND’SouzaDPolDJKalmanJMMarwickTH
Atrial fibrillation detection using single lead portable electrocardiographic monitoring: a systematic review and meta-analysis.
BMJ Open. 2018;8:e024178
doi: 10.1136/bmjopen-2018-02417810.1136/bmjopen-2018-024178PMC61444873022440418.CookeGDoustJSandersS
Is pulse palpation helpful in detecting atrial fibrillation? A systematic review.
J Fam Pract. 2006;55:130–134.1645178019.DoliwaPSFrykmanVRosenqvistM
Short-term ECG for out of hospital detection of silent atrial fibrillation episodes.
Scand Cardiovasc J. 2009;43:163–168. doi: 10.1080/140174308025934351909697710.1080/1401743080259343520.TielemanRGPlantingaYRinkesDBartelsGLPosmaJLCatorRHofmanCHoubenRP
Validation and clinical use of a novel diagnostic device for screening of atrial fibrillation.
Europace. 2014;16:1291–1295. doi: 10.1093/europace/euu0572482576610.1093/europace/euu057PMC414960821.KearleyKSelwoodMVan den BruelAThompsonMMantDHobbsFRFitzmauriceDHeneghanC
Triage tests for identifying atrial fibrillation in primary care: a diagnostic accuracy study comparing single-lead ECG and modified BP monitors.
BMJ Open. 2014;4:e004565
doi: 10.1136/bmjopen-2013-00456510.1136/bmjopen-2013-004565PMC40254112479325022.LauJKLowresNNeubeckLBriegerDBSyRWGallowayCDAlbertDEFreedmanSB
iPhone ECG application for community screening to detect silent atrial fibrillation: a novel technology to prevent stroke.
Int J Cardiol. 2013;165:193–194. doi: 10.1016/j.ijcard.2013.01.2202346524910.1016/j.ijcard.2013.01.22023.ChanPHWongCKPohYCPunLLeungWWWongYFSiuCW
Diagnostic performance of a smartphone-based photoplethysmographic application for atrial fibrillation screening in a primary care setting.
J Am Heart Assoc. 2016;5:e003428.2744450610.1161/JAHA.116.003428PMC501537924.McManusDDChongJWSoniASaczynskiJSEsaNNapolitanoCDarlingCEBoyerERosenRKFloydKC
PULSE-SMART: pulse-based arrhythmia discrimination using a novel smartphone application.
J Cardiovasc Electrophysiol. 2016;27:51–57. doi: 10.1111/jce.128422639172810.1111/jce.12842PMC476831025.ProesmansTMortelmansCVan HaelstRVerbruggeFVandervoortPVaesB
Mobile phone-based use of the photoplethysmography technique to detect atrial fibrillation in primary care: diagnostic accuracy study of the FibriCheck app.
JMIR Mhealth Uhealth. 2019;7:e12284
doi: 10.2196/122843091665610.2196/12284PMC645682526.BumgarnerJMLambertCTHusseinAACantillonDJBaranowskiBWolskiKLindsayBDWazniOMTarakjiKG
Smartwatch algorithm for automated detection of atrial fibrillation.
J Am Coll Cardiol. 2018;71:2381–2388. doi: 10.1016/j.jacc.2018.03.0032953506510.1016/j.jacc.2018.03.00327.WieselJFitzigLHerschmanYMessineoFC
Detection of atrial fibrillation using a modified microlife blood pressure monitor.
Am J Hypertens. 2009;22:848–852. doi: 10.1038/ajh.2009.981947879310.1038/ajh.2009.9828.BergeTLyngbakkenMNIhle-HansenHBrynildsenJPervezMOAagaardENVigenTKvisvikBChristophersenIESteineK
Prevalence of atrial fibrillation and cardiovascular risk factors in a 63-65 years old general population cohort: the Akershus Cardiac Examination (ACE) 1950 Study.
BMJ Open. 2018;8:e021704
doi: 10.1136/bmjopen-2018-02170410.1136/bmjopen-2018-021704PMC60746243006861729.SvennbergEEngdahlJAl-KhaliliFFribergLFrykmanVRosenqvistM
Mass screening for untreated atrial fibrillation: the STROKESTOP Study.
Circulation. 2015;131:2176–2184. doi: 10.1161/CIRCULATIONAHA.114.0143432591080010.1161/CIRCULATIONAHA.114.01434330.EngdahlJAnderssonLMirskayaMRosenqvistM
Stepwise screening of atrial fibrillation in a 75-year-old population: implications for stroke prevention.
Circulation. 2013;127:930–937. doi: 10.1161/CIRCULATIONAHA.112.1266562334356410.1161/CIRCULATIONAHA.112.12665631.Kemp GudmundsdottirKFredrikssonTSvennbergEAl-KhaliliFFribergLFrykmanVHijaziZRosenqvistMEngdahlJ
Stepwise mass screening for atrial fibrillation using N-terminal B-type natriuretic peptide: the STROKESTOP II study.
Europace. 2020;22:24–32. doi: 10.1093/europace/euz2553179014710.1093/europace/euz255PMC694505432.DoliwaRSobocinskiPDAnggårdh RoothEKullVFvon ArbinMWallénHRosenqvistM
Improved screening for silent atrial fibrillation after ischaemic stroke.
Europace. 2012;14:1112–1116. doi: 10.1093/europace/eur4312230808610.1093/europace/eur43133.KaasenbroodFHollanderMde BruijnSHDolmansCPTielemanRGHoesAWRuttenFH
Opportunistic screening versus usual care for diagnosing atrial fibrillation in general practice: a cluster randomised controlled trial.
Br J Gen Pract. 2020;70:e427–e433. doi: 10.3399/bjgp20X7081613198808410.3399/bjgp20X708161PMC698868034.TavernierRWolfMKatariaVPhlipsTHuysRTaghjiPLouwRHoeyweghenRVVandekerckhoveYKnechtS
Screening for atrial fibrillation in hospitalised geriatric patients.
Heart. 2018;104:588–593. doi: 10.1136/heartjnl-2017-3119812888303210.1136/heartjnl-2017-31198135.TurakhiaMPUllalAJHoangDDThanCTMillerJDFridayKJPerezMVFreemanJVWangPJHeidenreichPA
Feasibility of extended ambulatory electrocardiogram monitoring to identify silent atrial fibrillation in high-risk patients: the Screening Study for Undiagnosed Atrial Fibrillation (STUDY-AF).
Clin Cardiol. 2015;38:285–292. doi: 10.1002/clc.223872587347610.1002/clc.22387PMC465433036.SteinhublSRWaalenJEdwardsAMArinielloLMMehtaRREbnerGSCarterCBaca-MotesKFelicioneESarichT
Effect of a home-based wearable continuous ECG monitoring patch on detection of undiagnosed atrial fibrillation: the mSToPS randomized clinical trial.
JAMA. 2018;320:146–155. doi: 10.1001/jama.2018.81022999833610.1001/jama.2018.8102PMC658351837.RooneyMRSolimanEZLutseyPLNorbyFLLoehrLRMosleyTHZhangMGottesmanRFCoreshJFolsomAR
Prevalence and characteristics of subclinical atrial fibrillation in a community-dwelling elderly population: the ARIC Study.
Circ Arrhythm Electrophysiol. 2019;12:e007390
doi: 10.1161/CIRCEP.119.0073903160714810.1161/CIRCEP.119.007390PMC681438738.HeckbertSRAustinTRJensenPNFloydJSPsatyBMSolimanEZKronmalRA
Yield and consistency of arrhythmia detection with patch electrocardiographic monitoring: the Multi-Ethnic Study of Atherosclerosis.
J Electrocardiol. 2018;51:997–1002. doi: 10.1016/j.jelectrocard.2018.07.0273049776310.1016/j.jelectrocard.2018.07.027PMC627860839.LowresNNeubeckLSalkeldGKrassIMcLachlanAJRedfernJBennettAABriffaTBaumanAMartinezC
Feasibility and cost-effectiveness of stroke prevention through community screening for atrial fibrillation using iPhone ECG in pharmacies. The SEARCH-AF study.
Thromb Haemost. 2014;111:1167–1176. doi: 10.1160/TH14-03-02312468708110.1160/TH14-03-023140.ChanPHWongCKPohYCPunLLeungWWCWongYFWongMMYPohMZChuDWSSiuCW
Diagnostic performance of a smartphone-based photoplethysmographic application for atrial fibrillation screening in a primary care setting.
J Am Heart Assoc. 2016;5:e003428.2744450610.1161/JAHA.116.003428PMC501537941.HalcoxJPJWarehamKCardewAGilmoreMBarryJPPhillipsCGravenorMB
Assessment of remote heart rhythm sampling using the AliveCor heart monitor to screen for atrial fibrillation: the REHEARSE-AF Study.
Circulation. 2017;136:1784–1794. doi: 10.1161/CIRCULATIONAHA.117.0305832885172910.1161/CIRCULATIONAHA.117.03058342.ChanNYChoyCC
Screening for atrial fibrillation in 13 122 Hong Kong citizens with smartphone electrocardiogram.
Heart. 2017;103:24–31. doi: 10.1136/heartjnl-2016-3099932773353310.1136/heartjnl-2016-30999343.GuoYWangHZhangHLiuTLiangZXiaYYanLXingYShiHLiS; MAFA II Investigators. Mobile photoplethysmographic technology to detect atrial fibrillation.
J Am Coll Cardiol. 2019;74:2365–2375. doi: 10.1016/j.jacc.2019.08.0193148754510.1016/j.jacc.2019.08.01944.PerezMVMahaffeyKWHedlinHRumsfeldJSGarciaAFerrisTBalasubramanianVRussoAMRajmaneACheungL; Apple Heart Study Investigators. Large-scale assessment of a smartwatch to identify atrial fibrillation.
N Engl J Med. 2019;381:1909–1917. doi: 10.1056/NEJMoa19011833172215110.1056/NEJMoa1901183PMC811260545.GiebelGDGisselC
Accuracy of mHealth devices for atrial fibrillation screening: systematic review.
JMIR Mhealth Uhealth. 2019;7:e13641
doi: 10.2196/136413119933710.2196/13641PMC659842246.LowresNOlivierJChaoTFChenSAChenYDiederichsenAFitzmauriceDAGomez-DoblasJJHarbisonJHealeyJS
Estimated stroke risk, yield, and number needed to screen for atrial fibrillation detected through single time screening: a multicountry patient-level meta-analysis of 141,220 screened individuals.
PLoS Med. 2019;16:e1002903
doi: 10.1371/journal.pmed.10029033155373310.1371/journal.pmed.1002903PMC676076647.O’SullivanJWGriggSCrawfordWTurakhiaMPPerezMIngelssonEWheelerMTIoannidisJPAAshleyEA
Accuracy of smartphone camera applications for detecting atrial fibrillation: a systematic review and meta-analysis.
JAMA Netw Open. 2020;3:e202064
doi: 10.1001/jamanetworkopen.2020.20643224290810.1001/jamanetworkopen.2020.2064PMC712543348.FreedmanBPotparaTSLipGY
Stroke prevention in atrial fibrillation.
Lancet. 2016;388:806–817. doi: 10.1016/S0140-6736(16)31257-02756027610.1016/S0140-6736(16)31257-049.MartinezCKatholingAFreedmanSB
Adverse prognosis of incidentally detected ambulatory atrial fibrillation. A cohort study.
Thromb Haemost. 2014;112:276–286. doi: 10.1160/TH4-04-03832495305110.1160/TH4-04-0383PMC637498350.TsivgoulisGKatsanosAHKöhrmannMCasoVPerrenFPalaiodimouLDeftereosSGiannopoulosSEllulJKrogiasC
Duration of implantable cardiac monitoring and detection of atrial fibrillation in ischemic stroke patients: a systematic review and meta-analysis.
J Stroke. 2019;21:302–311. doi: 10.5853/jos.2019.010673159047410.5853/jos.2019.01067PMC678001851.https://www.heartline.com/. Accessed January 26, 202152.FreedmanBCammJCalkinsHHealeyJSRosenqvistMWangJAlbertCMAndersonCSAntoniouSBenjaminEJ; AF-Screen Collaborators. Screening for atrial fibrillation: a report of the AF-SCREEN International Collaboration.
Circulation. 2017;135:1851–1867. doi: 10.1161/CIRCULATIONAHA.116.0266932848383210.1161/CIRCULATIONAHA.116.02669353.JanuaryCTWannLSCalkinsHChenLYCigarroaJEClevelandJCJrEllinorPTEzekowitzMDFieldMEFurieKL
2019 AHA/ACC/HRS focused update of the 2014 AHA/ACC/HRS guideline for the management of patients with atrial fibrillation: a report of the American College of Cardiology/American Heart Association Task Force on Clinical Practice Guidelines and the Heart Rhythm Society in Collaboration With the Society of Thoracic Surgeons.
Circulation. 2019;140:e125–e151. doi: 10.1161/CIR.00000000000006653068604110.1161/CIR.000000000000066554.JonasDEKahwatiLCYunJDYMiddletonJCCoker-SchwimmerMAsherGN
Screening for atrial fibrillation with electrocardiography: evidence report and systematic review for the US Preventive Services Task Force.
JAMA. 2018;320:485–498. doi: 10.1001/jama.2018.41903008801510.1001/jama.2018.419055.HannonNSheehanOKellyLMarnaneMMerwickAMooreAKyneLDugganJMoroneyJMcCormackPM
Stroke associated with atrial fibrillation–incidence and early outcomes in the north Dublin population stroke study.
Cerebrovasc Dis. 2010;29:43–49. doi: 10.1159/0002559731989331110.1159/000255973PMC291440156.FurieKLGoldsteinLBAlbersGWKhatriPNeyensRTurakhiaMPTuranTNWoodKA; American Heart Association Stroke Council; Council on Quality of Care and Outcomes Research; Council on Cardiovascular Nursing; Council on Clinical Cardiology; Council on Peripheral Vascular Disease. Oral antithrombotic agents for the prevention of stroke in nonvalvular atrial fibrillation: a science advisory for healthcare professionals from the American Heart Association/American Stroke Association [published corrections appear in Stroke. 2012;43:e181. doi: 10.1161/STR.0b013e3182779f2f. Stroke. 2013;44:e20. doi: 10.1161/STR.0b013e318287336e].
Stroke. 2012;43:3442–3453. doi: 10.1161/STR.0b013e318266722a2285872857.Kolominsky-RabasPLWeberMGefellerONeundoerferBHeuschmannPU
Epidemiology of ischemic stroke subtypes according to TOAST criteria: incidence, recurrence, and long-term survival in ischemic stroke subtypes: a population-based study.
Stroke. 2001;32:2735–2740. doi: 10.1161/hs1201.1002091173996510.1161/hs1201.10020958.SchnabelRBHaeuslerKGHealeyJSFreedmanBBorianiGBrachmannJBrandesABustamanteACasadeiBCrijnsHJGM
Searching for atrial fibrillation poststroke: a white paper of the AF-SCREEN International Collaboration [published correction appears in Circulation. 2020;141:e99. doi:10.1161/CIR.0000000000000762].
Circulation. 2019;140:1834–1850. doi: 10.1161/CIRCULATIONAHA.119.0402673176526110.1161/CIRCULATIONAHA.119.04026759.KishoreAVailAMajidADawsonJLeesKRTyrrellPJSmithCJ
Detection of atrial fibrillation after ischemic stroke or transient ischemic attack: a systematic review and meta-analysis.
Stroke. 2014;45:520–526. doi: 10.1161/STROKEAHA.113.0034332438527510.1161/STROKEAHA.113.00343360.SannaTDienerHCPassmanRSDi LazzaroVBernsteinRAMorilloCARymerMMThijsVRogersTBeckersF; CRYSTAL AF Investigators. Cryptogenic stroke and underlying atrial fibrillation.
N Engl J Med. 2014;370:2478–2486. doi: 10.1056/NEJMoa13136002496356710.1056/NEJMoa131360061.ZungsontipornNLinkMS
Newer technologies for detection of atrial fibrillation.
BMJ. 2018;363:k3946
doi: 10.1136/bmj.k39463033310510.1136/bmj.k394662.SposatoLACiprianoLESaposnikGRuíz VargasERiccioPMHachinskiV
Diagnosis of atrial fibrillation after stroke and transient ischaemic attack: a systematic review and meta-analysis.
Lancet Neurol. 2015;14:377–387. doi: 10.1016/S1474-4422(15)70027-X2574810210.1016/S1474-4422(15)70027-X63.KaplanRMKoehlerJZieglerPDSarkarSZweibelSPassmanRS
Stroke risk as a function of atrial fibrillation duration and CHA2DS2-VASc score.
Circulation. 2019;140:1639–1646. doi: 10.1161/CIRCULATIONAHA.119.0413033156412610.1161/CIRCULATIONAHA.119.04130364.HartRGSharmaMMundlHKasnerSEBangdiwalaSIBerkowitzSDSwaminathanBLavadosPWangYWangY; NAVIGATE ESUS Investigators. Rivaroxaban for stroke prevention after embolic stroke of undetermined source.
N Engl J Med. 2018;378:2191–2201. doi: 10.1056/NEJMoa18026862976677210.1056/NEJMoa180268665.DienerHCSaccoRLEastonJDGrangerCBBernsteinRAUchiyamaSKreuzerJCroninLCottonDGrauerC; RE-SPECT ESUS Steering Committee and Investigators. Dabigatran for prevention of stroke after embolic stroke of undetermined source.
N Engl J Med. 2019;380:1906–1917. doi: 10.1056/NEJMoa18139593109137210.1056/NEJMoa181395966.KamelHLongstrethWTJrTirschwellDLKronmalRABroderickJPPaleschYYMeinzerCDillonCEwingISpilkerJA
The AtRial Cardiopathy and Antithrombotic Drugs In prevention After cryptogenic stroke randomized trial: rationale and methods.
Int J Stroke. 2019;14:207–214. doi: 10.1177/17474930187999813019678910.1177/1747493018799981PMC664538067.YanoYGreenlandPLloyd-JonesDMDaoudEGKoehlerJLZieglerPD
Simulation of daily snapshot rhythm monitoring to identify atrial fibrillation in continuously monitored patients with stroke risk factors.
PLoS One. 2016;11:e0148914
doi: 10.1371/journal.pone.01489142688233410.1371/journal.pone.0148914PMC475552968.ChenLYChungMKAllenLAEzekowitzMFurieKLMcCabePNoseworthyPAPerezMVTurakhiaMP; American Heart Association Council on Clinical Cardiology; Council on Cardiovascular and Stroke Nursing; Council on Quality of Care and Outcomes Research; and Stroke Council. Atrial fibrillation burden: moving beyond atrial fibrillation as a binary entity: a scientific statement from the American Heart Association.
Circulation. 2018;137:e623–e644. doi: 10.1161/CIR.00000000000005682966194410.1161/CIR.0000000000000568PMC846325869.VarmaNStamblerBChunS
Detection of atrial fibrillation by implanted devices with wireless data transmission capability.
Pacing Clin Electrophysiol. 2005;28suppl 1S133–S136. doi: 10.1111/j.1540-8159.2005.00083.x1568348010.1111/j.1540-8159.2005.00083.x70.ChanPHWongCKPunLWongYFWongMMChuDWSiuCW
Head-to-head comparison of the AliveCor heart monitor and microlife WatchBP office AFIB for atrial fibrillation screening in a primary care setting.
Circulation. 2017;135:110–112. doi: 10.1161/CIRCULATIONAHA.116.0244392802806610.1161/CIRCULATIONAHA.116.02443971.ProiettiMMairesseGHGoethalsPScaveeCVijgenJBlankoffIVandekerckhoveYLipGY; Belgian Heart Rhythm Week Investigators. A population screening programme for atrial fibrillation: a report from the Belgian Heart Rhythm Week screening programme.
Europace. 2016;18:1779–1786. doi: 10.1093/europace/euw0692717000010.1093/europace/euw06972.CaceresBAHickeyKTBakkenSBBivianoABGaranHGoldenthalILKoleckTAMasterson-CreberRTurchioeMRJiaH
Mobile electrocardiogram monitoring and health-related quality of life in patients with atrial fibrillation: findings from the iPhone Helping Evaluate Atrial Fibrillation Rhythm Through Technology (iHEART) study.
J Cardiovasc Nurs. 2020;35:327–336. doi: 10.1097/JCN.00000000000006463201525610.1097/JCN.0000000000000646PMC729973973.HickeyKTHauserNRValenteLERigaTCFrullaAPMasterson CreberRWhangWGaranHJiaHSciaccaRR
A single-center randomized, controlled trial investigating the efficacy of a mHealth ECG technology intervention to improve the detection of atrial fibrillation: the iHEART study protocol.
BMC Cardiovasc Disord. 2016;16:152
doi: 10.1186/s12872-016-0327-y2742263910.1186/s12872-016-0327-yPMC494729974.HealeyJSConnollySJGoldMRIsraelCWVan GelderICCapucciALauCPFainEYangSBailleulC; ASSERT Investigators. Subclinical atrial fibrillation and the risk of stroke.
N Engl J Med. 2012;366:120–129. doi: 10.1056/NEJMoa11055752223622210.1056/NEJMoa110557575.Van GelderICHealeyJSCrijnsHJGMWangJHohnloserSHGoldMRCapucciALauCPMorilloCAHobbeltAH
Duration of device-detected subclinical atrial fibrillation and occurrence of stroke in ASSERT.
Eur Heart J. 2017;38:1339–1344. doi: 10.1093/eurheartj/ehx0422832913910.1093/eurheartj/ehx04276.LopesRDAlingsMConnollySJBereshHGrangerCBMazuecosJBBorianiGNielsenJCConenDHohnloserSH
Rationale and design of the Apixaban for the Reduction of Thrombo-Embolism in Patients With Device-Detected Sub-Clinical Atrial Fibrillation (ARTESiA) trial.
Am Heart J. 2017;189:137–145. doi: 10.1016/j.ahj.2017.04.0082862537010.1016/j.ahj.2017.04.00877.MartinDTBersohnMMWaldoALWathenMSChoucairWKLipGYIpJHolcombRAkarJGHalperinJL; IMPACT Investigators. Randomized trial of atrial arrhythmia monitoring to guide anticoagulation in patients with implanted defibrillator and cardiac resynchronization devices.
Eur Heart J. 2015;36:1660–1668. doi: 10.1093/eurheartj/ehv1152590877410.1093/eurheartj/ehv11578.Al-TurkiAMarafiMRussoVProiettiREssebagV
Subclinical atrial fibrillation and risk of stroke: past, present and future.
Medicina (Kaunas). 2019;55:611.10.3390/medicina55100611PMC68433293154707879.GlotzerTVHellkampASZimmermanJSweeneyMOYeeRMarinchakRCookJParaschosALoveJRadoslovichG; MOST Investigators. Atrial high rate episodes detected by pacemaker diagnostics predict death and stroke: report of the Atrial Diagnostics Ancillary Study of the MOde Selection Trial (MOST).
Circulation. 2003;107:1614–1619. doi: 10.1161/01.CIR.0000057981.70380.451266849510.1161/01.CIR.0000057981.70380.4580.KirchhofPBlankBFCalvertMCammAJChlouverakisGDienerHCGoetteAHueningALipGYHSimantirakisE
Probing oral anticoagulation in patients with atrial high rate episodes: rationale and design of the Non-vitamin K antagonist Oral anticoagulants in patients with Atrial High rate episodes (NOAH-AFNET 6) trial.
Am Heart J. 2017;190:12–18. doi: 10.1016/j.ahj.2017.04.0152876020510.1016/j.ahj.2017.04.015PMC554617481.SwirynSOrlovMVBendittDGDiMarcoJPLloyd-JonesDMKarstEQuFSlawskyMTTurkelMWaldoAL; RATE Registry Investigators. Clinical implications of brief device-detected atrial tachyarrhythmias in a cardiac rhythm management device population: results from the Registry of Atrial Tachycardia and Atrial Fibrillation Episodes.
Circulation. 2016;134:1130–1140. doi: 10.1161/CIRCULATIONAHA.115.0202522775494610.1161/CIRCULATIONAHA.115.02025282.GoASReynoldsKYangJGuptaNLenaneJSungSHHarrisonTNLiuTISolomonMD
Association of burden of atrial fibrillation with risk of ischemic stroke in adults with paroxysmal atrial fibrillation: the KP-RHYTHM Study.
JAMA Cardiol. 2018;3:601–608. doi: 10.1001/jamacardio.2018.11762979994210.1001/jamacardio.2018.1176PMC614566383.PerinoACFanJAskariMHeidenreichPAKeungERaittMHPicciniJPZieglerPDTurakhiaMP
Practice variation in anticoagulation prescription and outcomes after device-detected atrial fibrillation.
Circulation. 2019;139:2502–2512. doi: 10.1161/CIRCULATIONAHA.118.0389883088043410.1161/CIRCULATIONAHA.118.038988PMC665219184.GarabelliPStavrakisSAlbertMKoomsonEParwaniPChohanJSmithLAlbertDXieRXieQ
Comparison of QT interval readings in normal sinus rhythm between a smartphone heart monitor and a 12-lead ECG for healthy volunteers and inpatients receiving sotalol or dofetilide.
J Cardiovasc Electrophysiol. 2016;27:827–832. doi: 10.1111/jce.129762702765310.1111/jce.1297685.CongreteSBintvihokMThongprayoonCBathiniTBoonphengBSharmaKChokesuwattanaskulRSrivaliNTanawuttiwatTCheungpasitpornW
Effect of obstructive sleep apnea and its treatment of atrial fibrillation recurrence after radiofrequency catheter ablation: a meta-analysis.
J Evid Based Med. 2018;11:145–151. doi: 10.1111/jebm.123133009130110.1111/jebm.1231386.KanagalaRMuraliNSFriedmanPAAmmashNMGershBJBallmanKVShamsuzzamanASSomersVK
Obstructive sleep apnea and the recurrence of atrial fibrillation.
Circulation. 2003;107:2589–2594. doi: 10.1161/01.CIR.0000068337.25994.211274300210.1161/01.CIR.0000068337.25994.2187.PathakRKMiddeldorpMEMeredithMMehtaABMahajanRWongCXTwomeyDElliottADKalmanJMAbhayaratnaWP
Long-term effect of goal-directed weight management in an atrial fibrillation cohort: a long-term follow-up study (LEGACY).
J Am Coll Cardiol. 2015;65:2159–2169. doi: 10.1016/j.jacc.2015.03.0022579236110.1016/j.jacc.2015.03.00288.VoskoboinikAKalmanJMDe SilvaANichollsTCostelloBNanayakkaraSPrabhuSStubDAzzopardiSViziD
Alcohol abstinence in drinkers with atrial fibrillation.
N Engl J Med. 2020;382:20–28. doi: 10.1056/NEJMoa18175913189351310.1056/NEJMoa181759189.RingwaldMCrichABeysardN
Smart watch recording of ventricular tachycardia: case study.
Am J Emerg Med. 2020;38:849.e3–849.e5. doi: 10.1016/j.ajem.2019.10.04010.1016/j.ajem.2019.10.0403178597390.WaksJWFeinASDasS
Wide complex tachycardia recorded with a smartphone cardiac rhythm monitor.
JAMA Intern Med. 2015;175:437–439. doi: 10.1001/jamainternmed.2014.75862562188010.1001/jamainternmed.2014.758691.ChongJWEsaNMcManusDDChonKH
Arrhythmia discrimination using a smart phone.
IEEE J Biomed Health Inform. 2015;19:815–824. doi: 10.1109/JBHI.2015.24181952583853010.1109/JBHI.2015.2418195PMC659971392.BilletSRollinAMondolyPMonteilBFournierPCariouEBlaye-FeliceMSGalinierMCarriéDLairezO
Hemodynamic consequences of premature ventricular contractions: association of mechanical bradycardia and postextrasystolic potentiation with premature ventricular contraction-induced cardiomyopathy.
Heart Rhythm. 2019;16:853–860. doi: 10.1016/j.hrthm.2018.12.0083055083510.1016/j.hrthm.2018.12.00893.ReedMJGrubbNRLangCCO’BrienRSimpsonKPadarengaMGrantATuckSKeatingLCoffeyF
Multi-centre randomised controlled trial of a smartphone-based event recorder alongside standard care versus standard care for patients presenting to the emergency department with palpitations and pre-syncope: the IPED (Investigation of Palpitations in the ED) study.
EClinicalMedicine. 2019;8:37–46. doi: 10.1016/j.eclinm.2019.02.0053119363610.1016/j.eclinm.2019.02.005PMC653755594.DavisJCRobertsonMCAsheMCLiu-AmbroseTKhanKMMarraCA
International comparison of cost of falls in older adults living in the community: a systematic review.
Osteoporos Int. 2010;21:1295–1306. doi: 10.1007/s00198-009-1162-02019584610.1007/s00198-009-1162-095.HeinrichSRappKRissmannUBeckerCKönigHH
Cost of falls in old age: a systematic review.
Osteoporos Int. 2010;21:891–902. doi: 10.1007/s00198-009-1100-11992449610.1007/s00198-009-1100-196.LeeHShinSYSeoMNamGBJooS
Prediction of ventricular tachycardia one hour before occurrence using artificial neural networks.
Sci Rep. 2016;6:32390
doi: 10.1038/srep323902756132110.1038/srep32390PMC499995297.Au-YeungWMReinhallPGBardyGHBruntonSL
Development and validation of warning system of ventricular tachyarrhythmia in patients with heart failure with heart rate variability data.
PLoS One. 2018;13:e0207215
doi: 10.1371/journal.pone.02072153042788010.1371/journal.pone.0207215PMC623535898.KwonJMLeeYLeeYLeeSParkJ
An algorithm based on deep learning for predicting in-hospital cardiac arrest.
J Am Heart Assoc. 2018;7:e008678.2994591410.1161/JAHA.118.008678PMC606491199.Praveen KumarDAmgothTAnnavarapuCSR
Machine learning algorithms for wireless sensor networks: a survey.
Information Fusion. 2019;49:1–25.100.RickardJAhmedSBaruchMKlocmanBMartinDOMenonV
Utility of a novel watch-based pulse detection system to detect pulselessness in human subjects.
Heart Rhythm. 2011;8:1895–1899. doi: 10.1016/j.hrthm.2011.07.0302180239310.1016/j.hrthm.2011.07.030101.ChanJReaTGollakotaSSunshineJE
Contactless cardiac arrest detection using smart devices.
NPJ Digit Med. 2019;2:52
doi: 10.1038/s41746-019-0128-73130439810.1038/s41746-019-0128-7PMC6584582102.WuOBriggsAKempTGrayAMacIntyreKRowleyJWillettK
Mobile phone use for contacting emergency services in life-threatening circumstances.
J Emerg Med. 2012;42:291–298.e3. doi: 10.1016/j.jemermed.2011.02.0222214266910.1016/j.jemermed.2011.02.022103.RinghMRosenqvistMHollenbergJJonssonMFredmanDNordbergPJärnbert-PetterssonHHasselqvist-AxIRivaGSvenssonL
Mobile-phone dispatch of laypersons for CPR in out-of-hospital cardiac arrest.
N Engl J Med. 2015;372:2316–2325. doi: 10.1056/NEJMoa14060382606183610.1056/NEJMoa1406038104.BolleSRSchollJGilbertM
Can video mobile phones improve CPR quality when used for dispatcher assistance during simulated cardiac arrest?
Acta Anaesthesiol Scand. 2009;53:116–120. doi: 10.1111/j.1399-6576.2008.01779.x1903256910.1111/j.1399-6576.2008.01779.xPMC2659378105.ChoaMParkIChungHSYooSKShimHKimS
The effectiveness of cardiopulmonary resuscitation instruction: animation versus dispatcher through a cellular phone.
Resuscitation. 2008;77:87–94. doi: 10.1016/j.resuscitation.2007.10.0231816411910.1016/j.resuscitation.2007.10.023106.MerchantRMAbellaBSAbotsiEJSmithTMLongJATrudeauMELearyMGroeneveldPWBeckerLBAschDA
Cell phone cardiopulmonary resuscitation: audio instructions when needed by lay rescuers: a randomized, controlled trial.
Ann Emerg Med. 2010;55:538–543.e1. doi: 10.1016/j.annemergmed.2010.01.0202020271910.1016/j.annemergmed.2010.01.020107.YouJSParkSChungSPParkJW
Performance of cellular phones with video telephony in the use of automated external defibrillators by untrained laypersons.
Emerg Med J. 2008;25:597–600. doi: 10.1136/emj.2008.0585031872371510.1136/emj.2008.058503108.KalzMLenssenNFelzenMRossaintRTabuencaBSpechtMSkorningM
Smartphone apps for cardiopulmonary resuscitation training and real incident support: a mixed-methods evaluation study.
J Med Internet Res. 2014;16:e89
doi: 10.2196/jmir.29512464736110.2196/jmir.2951PMC3978555109.PaalPPircherIBaurTGruberEStrasakAMHerffHBruggerHWenzelVMitterlechnerT
Mobile phone-assisted basic life support augmented with a metronome.
J Emerg Med. 2012;43:472–477. doi: 10.1016/j.jemermed.2011.09.0112225760010.1016/j.jemermed.2011.09.011110.WeisfeldtMLSitlaniCMOrnatoJPReaTAufderheideTPDavisDDreyerJHessEPJuiJMaloneyJ; ROC Investigators. Survival after application of automatic external defibrillators before arrival of the emergency medical system: evaluation in the resuscitation outcomes consortium population of 21 million.
J Am Coll Cardiol. 2010;55:1713–1720. doi: 10.1016/j.jacc.2009.11.0772039487610.1016/j.jacc.2009.11.077PMC3008654111.SakaiTIwamiTKitamuraTNishiyamaCKawamuraTKajinoKTanakaHMarukawaSTasakiOShiozakiT
Effectiveness of the new ‘Mobile AED Map’ to find and retrieve an AED: a randomised controlled trial.
Resuscitation. 2011;82:69–73. doi: 10.1016/j.resuscitation.2010.09.4662105113010.1016/j.resuscitation.2010.09.466112.HatakeyamaTNishiyamaCShimamotoTKiyoharaKKiguchiTChidaIIzawaJMatsuyamaTKitamuraTKawamuraT
A smartphone application to reduce the time to automated external defibrillator delivery after a witnessed out-of-hospital cardiac arrest: a randomized simulation-based study.
Simul Healthc. 2018;13:387–393. doi: 10.1097/SIH.00000000000003052965941310.1097/SIH.0000000000000305PMC6303130113.Neves BriardJGrou-BoileauFEl BashtalyASpenardCde ChamplainFHomierV
Automated external defibrillator geolocalization with a mobile application, verbal assistance or no assistance: a pilot randomized simulation (AED G-MAP).
Prehosp Emerg Care. 2019;23:420–429. doi: 10.1080/10903127.2018.15110173011122210.1080/10903127.2018.1511017114.BoutilierJJBrooksSCJanmohamedAByersABuickJEZhanCSchoelligAPCheskesSMorrisonLJChanTCY; Rescu Epistry Investigators. Optimizing a drone network to deliver automated external defibrillators.
Circulation. 2017;135:2454–2465. doi: 10.1161/CIRCULATIONAHA.116.0263182825483610.1161/CIRCULATIONAHA.116.026318PMC5516537115.ClaessonABäckmanARinghMSvenssonLNordbergPDjärvTHollenbergJ
Time to delivery of an automated external defibrillator using a drone for simulated out-of-hospital cardiac arrests vs emergency medical services.
JAMA. 2017;317:2332–2334. doi: 10.1001/jama.2017.39572860952510.1001/jama.2017.3957PMC5815004116.AndeliusLMalta HansenCLippertFKKarlssonLTorp-PedersenCKjær ErsbøllAKøberLCollatz ChristensenHBlombergSNGislasonGH
Smartphone activation of citizen responders to facilitate defibrillation in out-of-hospital cardiac arrest.
J Am Coll Cardiol. 2020;76:43–53. doi: 10.1016/j.jacc.2020.04.0733261616210.1016/j.jacc.2020.04.073

## 4. Comorbidities

**Figure 6.**
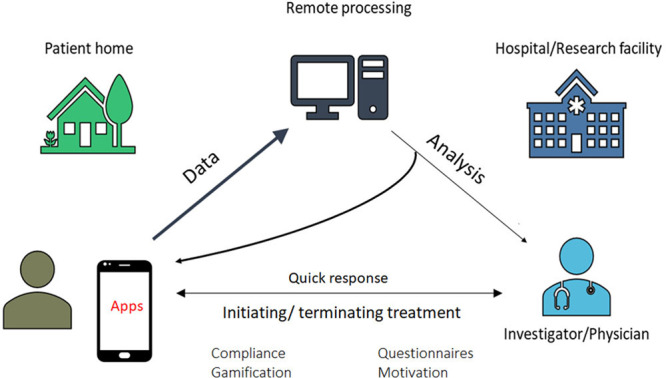
**Digital applications can integrate patient-relayed information of sensor and clinical information with automatic remote analysis but also permit patients to receive advice and treatment adjustments from physicians directly.**

A large proportion of arrhythmias are influenced by coexisting conditions. Their management may directly affect arrhythmia recurrence and outcome. Thus, lifestyle modifications and management of comorbid conditions (Figure 5) is becoming an objective of arrhythmia management (Chung 2020)^1^ and received a class 1 recommendation in most recent guidelines (January 2019).^2^ mHealth has significant potential for facilitating these interventions (Figure 6).

### 4.1. Ischemic Heart Disease

Early management (eg, primary angioplasty) of acute ischemic syndromes may reduce infarct territory and ventricular arrhythmias, thereby improving outcome. AF after myocardial infarction worsens prognosis (Pizzetti 2011).^3^

#### At Home

ST-segment monitoring technology embedded in conventionally indicated ICDs when tested in a randomized crossover study suggested a reduction in the time from the onset of ischemia to presentation to hospital (Gibson 2019, Holmes 2019).^4,5^ The AngelMed Guardian system (Angel Medical Systems, Eatontown, NJ) is approved for use in the United States for patients with prior acute coronary syndrome who remain at high risk for recurrent acute coronary syndrome. For lower risk patients, mHealth may improve symptom recognition and earlier presentation, that is, symptom-to-door time (Moser 2006).^6^

Wearable devices that continuously monitor physiological data promise detection, and possibly preemption, of the early stages of MI, by alerting patient or health care team early. A noninvasive device consisting of a 3-lead ECG linked wirelessly to a dedicated mobile device has recently been described (Van Heuverswyn 2019).^7^ Three-lead ECG tracings (as well as derived augmented limb leads) can be recorded with commercially available smartwatches (Avila 2019).^8^ Limitations of this approach are the need for the patient or a bystander to possess the device or app and be familiar with its use, before the onset of symptoms.

An emerging technology (www.heartbeam.com) uses a credit card–sized device that is pressed against the user’s chest (Figure 3). It collects ECG signals using a novel 3-dimensional vector approach. The signals are sent to the cloud, where they are analyzed and compared with the patient’s asymptomatic baseline reading. A proprietary algorithm combines the signal analysis with the patient’s history and reported symptoms. This information, along with a diagnostic recommendation and ECG waveforms, is sent to the patient’s physician, who makes a final determination and informs the patient. This system is used by patients in the telehealth setting to assess whether chest pain is the result of a myocardial infarction.

#### Emergency Teams

The next step of patient care involved transmission of ECGs by emergency responders in the field to hospitals for review and triage and was shown to result in shorter door-to-balloon time, lower peak troponin and creatine phosphokinase levels, higher postinfarction left ventricular ejection fraction, and shorter length of stay compared with control patients whose ECGs were not transmitted (Clemmensen 2010, Sanchez-Ross 2011).^9,10^ This paradigm has now been widely implemented. Technical factors, such as transmission failure and lack of network coverage, are the main impediments to adoption of such systems.

#### Post-Hospital Care

This is often confusing for patients, who often exhibit a poor understanding of their medications, follow-up procedures, and future appointments (Horwitz 2013, Ziaeian 2012).^11,12^ This contributes to frequent hospital readmissions. Mobile technologies may enable individualized contact between patients and health care providers. Phone calls led to a modest improvement in medication adherence in patients with coronary artery disease in one large randomized controlled trial (Vollmer 2014).^13^ Text messaging was shown to increase medication adherence and improved cardiovascular risk factors (Chow 2015, Unal 2018).^14,15^ Available evidence is limited by short-term follow-up and self-reported adherence (Shariful Islam 2019).^16^ Success may depend on personalized messages with tailored advice, the ability to respond to texts, timing messages to coincide with medication doses, higher frequency of messages, and the use of additional apps or websites (Park 2014).^17^ Interoperability with the electronic medical record (EMR) may facilitate this approach.

#### Cardiac Rehabilitation

This was shown to improve health outcomes among patients with heart disease but is underutilized. The Million Hearts Cardiac Rehabilitation Collaborative aims to increase participation rates to ≥70% by 2022 (Ritchey 2020).^18^ Mobile apps and linked sensors to measure HR, respiration rate, and exercise parameters may overcome traditional limitations of availability, cost, and convenience and be more acceptable to some patients (Zwisler 2016).^19^ A randomized controlled trial center-based and mobile rehabilitation found improved uptake, adherence, and completion with home-based cardiac rehabilitation in postinfarction patients (Varnfield 2014^20^; Section 4.2.2).

### 4.2. Heart Failure

HF is widely prevalent, costly to manage, and degrades patient outcomes (Benjamin 2017, Albert 2019).^21,22^ HF may trigger AF and ventricular arrhythmias. Conversely, AF may precipitate HF. RM of, for example, dietary and medication adherence (Section 4.6.2), detection of arrhythmias (Section 3), intercurrent ischemia (Section 4.1), orthopnea, changes in HR, activity, and sleep (Section 4.5), may enable remote adjustment of management to reduce emergency department visits and unplanned HF-related hospitalizations. If scalable, RM coupled with mobile communication could prove to reduce costs associated with HF.

Despite promise, most large, multicenter randomized trials failed to demonstrate improved outcomes of RM in patients with HF (Table 4; Boyne 2012, Chaudhry 2010, Dickinson 2018, Koehler 2011, Ong 2016, Stehlik 2020, Takahashi 2012).^23–29^ Combination algorithms based on multiple parameters may be valuable (Ono 2017).^30^ One trial stands out. The TIM-HF2 trial (Telemedical Interventional Management in Heart Failure II) randomized patients with HF to either remote patient management plus usual care or to usual care only and was followed up for over a year (Koehler 2018).^31^ The results showed reduction in the combined end point of percentage of days lost due to unplanned hospitalization and all-cause mortality. However, cardiovascular mortality was similar between RM and standard care groups. Implanted devices that monitor pulmonary arterial pressure may be beneficial in select patients when used in structured programs (Dickinson 2018).^29^ The positive findings of the CHAMPION trial (CardioMEMS Heart Sensor Allows Monitoring of Pressure to Improve Outcomes in NYHA Functional Class III Heart Failure Patients) and subsequent FDA approval have renewed interest in remote patient management for patients with HF (Abraham 2016, Carbo 2018, Desai 2017).^32–34^ This requires daily download of hemodynamic data and a prespecified medical treatment plan. An app is also available that illustrates patient compliance with monitoring, alerts the patient when transmissions are not received, shows medication reminders, and allows for medication reconciliation and titration.

**Table 4.**
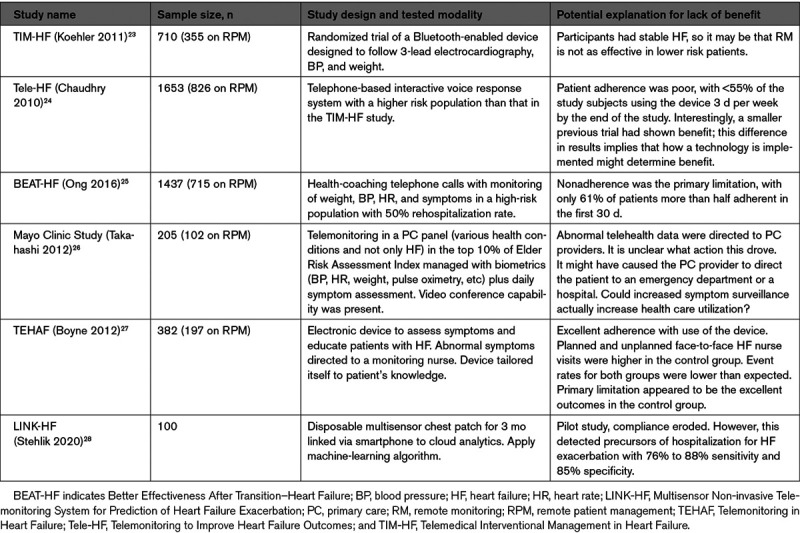
Randomized Trials With Neutral Results Based on External-Device RPM

#### 4.2.1. Mobile Technologies for Managing HF

The concept of coupling RM and mobile cellular technologies is attractive for the HF community (Carbo 2018, Cipresso 2012).^33,35^ HR (ECG), BP, and weight were the most frequently monitored parameters. Sensors that detect respiratory rate and pattern by detecting movement of the chest wall via pressure, stretch, or accelerometry may have applications in HF. Detecting breathing via microphone (sounds), change in impedance, or pulse oximetry are other possible means to monitor respiratory function. Some of these modalities could be integrated into smart clothing (Molinaro 2018).^36^

Some trials included also alert reminders of medication use, voice messages on educational tips, video education, and tracking of physical activity (Section 4.6.1). Patients were mostly monitored daily and followed for an average of 6 months. A reduction was seen in HF-related hospital days (Carbo 2018).^33^ High rates of patient engagement, acceptance, usage, and adherence have been reported in some trials but not others (Chaudhry 2010, Hamilton 2018).^37,38^

Preliminary results using a disposable multisensor chest patch in the LINK-HF study (Multisensor Non-invasive Telemonitoring System for Prediction of Heart Failure Exacerbation) were encouraging (Stehlik 2020),^28^ detecting precursors of hospitalization for HF exacerbation with 76% to 88% sensitivity and 85% specificity, 1 week before clinical manifestations.

#### 4.2.2. Hybrid Telerehabilitation in Patients With HF

Exercise training is recommended for all stable HF patients (Piepoli 2011, Ponikowski 2016).^39,40^ Hybrid cardiac telerehabilitation is a novel approach. Telerehabilitation is the supervision and performance of comprehensive cardiac rehabilitation at a distance, encompassing telemonitoring (minimally intrusive, often involving sensors), teleassessment (active remote assessment), telesupport (supportive televisits by nurses and psychological support), teletherapy (actual interactive therapy), telecoaching (support and instruction for therapy), teleconsulting, and telesupervision of exercise training (Piotrowicz 2016).^41^ Various devices have been described, from HR monitoring (Smart 2005)^42^ and transtelephonic electrocardiographic monitoring (Koudi 2006)^43^ to tele-ECG monitoring via a remote device (Piotrowicz 2015)^44^ and real-time ECG and voice transtelephonic monitoring (Ades 2000).^45^

Home-based telerehabilitation was demonstrated to be safe, effective with high adherence among HF patients. It improves physical capacity (Piotrowicz 2015)^46^ and psychological status (Piotrowicz 2016),^47^ with similar quality of life improvement to standard rehabilitation (Piotrowicz 2015).^48^ The first randomized, prospective, multicenter study (TELEREH-HF [Telerehabilitation in Heart Failure Patients]) showed that hybrid telerehabilitation and telecare in patients with HF was more effective than usual care in improving peak Vo_2_, 6-minute walk distance, and quality of life, although not associated with reduction of 24-month mortality and hospitalization except in the most experienced centers (Piotrowicz 2020, Piotrowicz 2019).^49,50^

The recent Scientific Statement from the American Association of Cardiovascular and Pulmonary Rehabilitation, the American Heart Association, and the American College of Cardiology indicates that home-based rehabilitation using telemedicine is a promising new direction (Thomas 2019).^51^

### 4.3. Diabetes

Diabetes is a strong risk factor for the development of morbidity and mortality associated with a range of cardiovascular diseases. Metabolic syndrome (elevated blood glucose and insulin resistance) acts via multiple mechanisms resultant in micro- and macrovascular complications, development of autonomic neuropathy, diastolic dysfunction, renal failure, and AF. Important management goals are lifestyle changes (eg, diet and activity: see Sections 4.6.1 and 4.6.2) to prevent disease development and tight glycemic control, especially for type 1 diabetes, which demands lifelong rigorous self-monitoring (Balakumar 2016, Donnellan 2019, Goudis 2015, Wang 2019, Wilkinson 2019, Wingerter 2019).^52–57^ mHealth modalities for self-management were recommended recently by the European Society of Cardiology guidelines on diabetes and cardiovascular diseases to (Cosentino 2019).^58^

Glycemic control may reduce AF development and recurrence (Chao 2012, Chang 2014, Gu 2011, Otake 2009).59–62

Mobile apps can facilitate self-management by reminding regular assessment of required parameters and medications to take and provide educational tools and motivational support. Regular transmission of blood glucose levels from patients to their physicians can be based on SMS, email, or diverse web-based services. Bluetooth-enabled glucose meters are frequently used (Andres 2019, Garabedian 2015).^63,64^ BlueStar (Welldoc, Columbia, MD), first to receive the US FDA clearance for diabetes management, comes with an app that requires a physician prescription and enables patients to titrate insulin dosing by using the proprietary insulin calculator. The Freestyle LibreLink app (Abbott Laboratories, Abbott Park, IL) reads an associated continuous glucose monitoring device and displays trends (Fokkert 2017).^65^

Stand-alone diabetes management apps have recently been reviewed (Fleming 2020).^66^ Short-term measures, such as HbA1c (glycated hemoglobin), may be improved by such apps in conjunction with clinical support, but many have suboptimal usability (Veazie 2018).^67^ Phone-based interventions were associated with improved glycemic control as compared with standard care (Liang 2011, Pillay 2015, Saffari 2014, Fokkert 2019).^68–71^ Efficacy for improving glycemic control in randomized controlled trials has shown mixed results (Agarwal 2019, Quinn 2011).^72,73^ Meta-analyses indicate that mobile phone interventions for self-management reduced HbA1c modestly by 0.2% to 0.5% over a median of 6-month follow-up duration, with a greater reduction in patients with type 2 compared with type 1 diabetes (Pal 2014).^74^ A significant impact on clinical outcomes may affect health care expenditures by reducing the need for in-person contact with health care providers, preventing hospital admissions and improving prognosis. In a retrospective study, the use of mHealth technologies was associated with a 21.9% reduction in medical spending than a control group during the first year (Whaley 2019).^75^ Key determinants to successful uptake of decision support apps will be their user-friendliness and complexity and the delivery of electronic communications and feedback to the patient.

### 4.4. Hypertension

#### Hypertension, because of its high prevalence, provides the highest attributable risk for the development of AF (Huxley 2011).76

mHealth strategies for hypertension comprise a continuum of solutions, used by consumers or health care providers, and include wireless diagnostic and clinical decision support (CDS) tools, aiming to monitor health status and improve health outcomes. BP telemonitoring is one of the most commonly used strategies and includes remote data transmission of BP and clinical information from patients in their home or from a community setting to a central service, where they are reviewed by a managing physician for treatment adjustments. Several clinical trials have shown that BP telemonitoring might be more effective than usual care in achieving target BP (Bosworth 2011, Kim 2015, McManus 2010).^77–79^ A meta-analysis showed that, compared with usual care, BP telemonitoring improved office systolic BP and diastolic BP by 3.99 mm Hg ([95% CI, 5.06–2.93] *P*<0.001) and 1.99 mm Hg ([95% CI, −2.60 to −1.39] *P*<0.001), respectively (Duan 2017).^80^ BP telemonitoring nested in a more complex intervention, including additional support, as face-to-face counseling, telecounseling, education, behavioral management, medication management, and adherence contracts, led to additional and more sustainable benefit (Duan 2017, Tucker 2017).^80,81^ mHealth has the potential to promote patient self-management, as a complement to the doctor’s intervention, and encourage greater participation in medical decision-making. Indeed, the TASMINH4 (Telemonitoring and/or Self-Monitoring of Blood Pressure in Hypertension) unblinded, randomized controlled trial showed that patients who used self-monitoring of BP to titrate antihypertensives, with or without telemonitoring, achieved better BP control than those assigned to usual care (McManus 2018).^82^ The self-monitoring group that used telemonitoring achieved lower BP quicker than the self-monitoring group not receiving telemonitoring support, but readings were not significantly different at 1 year of follow-up. Cost-effectiveness analysis suggests that self-monitoring in this context is cost-effective by the NICE (National Institute for Health and Care Excellence) criteria, that is, costing well under £20 000 per quality-adjusted life year (Monahan 2019).^83^

Although mHealth options may aid hypertension management, technological barriers, high costs, heterogeneity of solutions and technologies, and lack of standards challenge clinical implementation. The 2019 European Society of Cardiology guidelines on hypertension stress the importance of self-monitoring and underline the potential use of smartphone-based solutions. Nevertheless, they do not recommend the use of mobile apps as independent mean of BP measurements (Williams 2018).^84^

### 4.5. Disorders Including Sleep Apnea

Sleep disorders are widely prevalent and contribute to cardiovascular risk and arrhythmias, especially AF (Daghlas 2019, Hirshkowitz 2015, Mehra 2006, May 2016, May 2017; Institute of Medicine Report: Sleep Disorders and Sleep Deprivation: an Unmet Public Health Problem, Institute of Medicine (US) Committee on Sleep Medicine and Research: www.ncbi.nlm.nih.gov/books/NBK19961; Section 4.2.1).^85–90^ This may be because sleep disturbance is intimately tied to circadian rhythms and sympathovagal balances (Burgess 1997).^91^ Standard sleep disorder diagnostics have been validated but require technical support for data acquisition and scoring. For example, polysomnography has long been considered the gold standard for acquisition of rich multimodal cardio-neurorespiratory objective physiological data to ascertain sleep architecture, total sleep time, and cardiorespiratory abnormalities and is primarily used for the diagnosis of obstructive sleep apnea. Actigraphy has the advantage of collecting objective data over days and nights to characterize sleep-wake patterning and provide measures of total sleep time, sleep efficiency, and sleep onset latency in addition to surrogate circadian measures. However, such tests are obtrusive and expensive.

- Treating sleep apnea may reduce AF burden (Qureshi 2015, Youssef 2018).^92,93^

Consumer technology directed to sleep medicine may revolutionize the detection and treatment of sleep disorders. Since such apps are preinstalled on many smartphones, sleep tracking may be among the most widely applied facets of mHealth (Khosla 2018).^94^ Applications include mobile device applications, wearable devices, embedded devices (in the individual’s sleep environment), rings (https://bodimetrics.com/product/circul-sleep-and-fitness-ring), integration of accessory diagnostic monitoring (eg, oximetry and ECG monitoring), and sleep therapy adherence monitoring. Several commercially available wearable devices measure total sleep time accurately but not more detailed parameters such as sleep efficiency and different sleep stages (Mantua 2016).^95^ Preliminary data suggest that wearable devices may be capable of detecting sleep apnea with good accuracy compared with gold standard polysomnography (Selvaraj 2014)^96^ and transform the approach to sleep disorder screening, diagnosis, and treatment. Sleep irregularity diagnosed by 7-day wrist actigraphy was linked to risk of cardiovascular events (Huang 2020).^97^ Preliminary studies indicated that use of wearables may permit behavior modifications that improve sleep quality (Berryhill 2020).^98^ In this regard, mHealth applications to sleep diagnosis and treatment promise facilitation of rhythm control.

### 4.6. Lifestyle

#### 4.6.1. Physical Activity

Physical activity is any bodily movement from skeletal muscle contraction to increase energy expenditure above basal level (Figure 5). Athletic activity varies from recreational sports to competitive events. There is a compelling evidence that regular aerobic exercise at the levels recommended by the Physical Activity Guidelines Advisory Committee reduces the risk of a variety of cardiovascular conditions, including AF (Everett 2011, Mozaffarian 2008, Piercy 2018).^99–101^ However the majority of the population is not engaged in physical activity at the recommended levels (Piercy 2018).^101^ Among patients with cardiovascular disease, patient activity measured automatically by ICDs correlated with survival following ICD implantation (Kramer 2015).^102^ Fitness represents an enormous market for mobile technologies and significant opportunity to improve the health of a wide range of mHealth consumers. In 2017, over 318 000 fitness and health apps were available, almost double the number 2 years prior (IQUVIA Institute, 2017).^103^ Many of these recreational apps monitor daily physical activity and support a healthy lifestyle by counting the number of steps daily, online training, and motivation coaching (McConnell 2018).^104^

- Cardiorespiratory fitness has an inverse relationship to AF burden (Faselis 2015).^105^
- Improvement in exercise capacity of 2 METs in overweight individuals may double freedom from AF (Pathak 2015).^106^

Consumer grade fitness technology includes individual fitness trackers that can stand alone, a fitness tracker that is coupled with a companion app, or an app that can be downloaded onto a smartphone, which then utilizes various features of the smartphone to measure activity and sleep. The accuracy of these measurements varies between different products and between measures within the same product (Rosenberger 2016).^107^ Furthermore, while step counting is long established, measuring the intensity of exercise is more complex. Although fitness technology has the exciting potential to increase physical activity by promoting goal setting and providing feedback, its effectiveness in motivating positive behavioral change remains unclear (Sullivan 2017).^108^

One cautionary tale is the study by Jakicic et al^109^ that examined the effectiveness of a lifestyle intervention with or without a fitness tracker (Jakicic 2016). Two groups received instruction to promote physical activity and dietary restriction. Six months into the intervention, half of the participants were provided with an upper arm fitness tracker and web-based support accompanying the device. The other half logged and tracked their activity and diet on a study website. Of note, the group that wore the tracker lost less weight than the group who did not. Moreover, changes in physical activity between the two groups were not significantly different. These results cast doubt on the effectiveness of fitness trackers in promoting greater physical activity, and thus, further data are required to assess the impact of this approach (Section 5).

##### Competitive Athletes

These are a unique category. Endurance athletes may may have increased AF risk (Abdulla 2009, Anderson 2013).^110,111^ Remote evaluation of ECG recordings may be useful in countries that perform preparticipation ECG screening (Brunetti 2014, Orchard 2019).^112,113^ Mobile devices and apps provide complex data that can be used as a self-monitoring tool for managing training (Aroganam 2019, Li 2016, Peake 2018, Peart 2019, Seshadri 2019).^114–118^ Exercise load and performance level can be accessed on a regular basis by coaches and athletes. Training guided by daily monitoring of HRV parameters has also been proposed, but data are limited (Coppetti 2017, Dobbs 2019, Singh 2018).^119–121^ Mobile devices provide the possibility of online real-time monitoring during indoor and outdoor training and competitions. Monitoring of HR provides both information on performance and level of training but can also provide valuable information regarding heart rhythm irregularity suggestive of arrhythmias. Any kind of paroxysmal arrhythmia related to sport participation and detected by mobile devices designed merely for HR assessment should trigger further cardiological evaluation. Having in mind data indicating that sports participation may be associated with higher risk of development of AF mobile devices may serve as valuable screening tool for AF detection.

Importantly, mHealth solutions enable easy access to athletes’ medical data. The latter approach can be of special interest in management of athletes’ health during competitions abroad.

#### 4.6.2. Diet

In 2010, the AHA promulgated Life’s Simple 7 as a public health strategy to improve cardiovascular health with the motto “7 Small Steps to Big Changes. It’s easy and simple. Anyone can do it. Start with one or two!” Unfortunately, research has shown that this strategy is anything but simple: virtually no adults (<1%) are compliant with all recommendations, and 42% are compliant with only 0 to 2 recommendations (Folsom 2011).^122^ Although there is ample evidence that weight loss and maintaining an ideal weight are beneficial in reducing AF burden and symptoms, compliance with this recommendation is poor; the reasons include among others the inability to track food intake (Abed 2013, Donnellan 2019, Pathak 2015).^123–125^

- Weight loss combined with risk factor modification is a class 1 (B-R) recommendation in treatment of AF (January 2019).^2^
- More than 10% weight reduction/target BMI <27 kg/m^2^ reduces AF burden (Pathak 2015).^125^

There are currently many consumer-oriented mobile phone–based applications designed for tracking food intake, but their utility for use in carbohydrate counting is limited due to their design (El-Gayar 2013).^126^ Commonly, these consumer-oriented apps require multiple steps. As an example, the user types in the food consumed and then scrolls through the search results to match with the program’s food and nutrient database. Next, after finding a matching food type, the user must estimate and enter an amount. These apps require significant user input and time burden along with high possibility of error. In addition, they are also plagued by uncertain accuracy. Recently, research has shown that nutrient calculations from leading nutrition tracking apps tended to be lower than results from using 24-hour recall with analysis by the Nutrition Data System for Research—a research-level dietary analysis software (Griffiths 2018).^127^

By contrast, a visual image–based app such as the Technology-Assisted Dietary Assessment (TADA) system directly addresses the aforementioned shortcomings (Boushey 2017, Six 2010, Zhu 2010).^128–130^ This is in research phase. The TADA system consists of 2 main components: (1) a smartphone app that runs on either iPhones (iOS) or Android devices: the Mobile Food Record and (2) cloud-based server that communicates with the Mobile Food Record, processes, and stores the food images. Using the TADA system, a person takes a photo of the meal they are planning to eat using their smartphone’s camera. The use of geometric models has permitted the TADA system to use a single image of a meal to estimate portion size to within 15% of the actual amount (Fang 2015).^131^ Hence, smartphone-based technology such as the TADA system can facilitate tracking of food intake, which in turn can potentially help with weight management.

Despite the profusion of diet- and weight-related apps, and the interest in weight loss in the community, there remains a dearth of high-quality evidence that these apps are actually effective (Dounavi 2019).^132^ There remains a need for further evidence development before specific apps or other mHealth technology can be recommended or prescribed.

## 

References: Section 41.ChungMKEckhardtLLChenLYAhmedHMGopinathannairRJoglarJANoseworthyPAPackQRSandersPTrulockKM; American Heart Association Electrocardiography and Arrhythmias Committee and Exercise, Cardiac Rehabilitation, and Secondary Prevention Committee of the Council on Clinical Cardiology; Council on Arteriosclerosis, Thrombosis and Vascular Biology; Council on Cardiovascular and Stroke Nursing; and Council on Lifestyle and Cardiometabolic Health. Lifestyle and risk factor modification for reduction of atrial fibrillation: a scientific statement from the American Heart Association.
Circulation. 2020;141:e750–e772. doi: 10.1161/CIR.00000000000007483214808610.1161/CIR.00000000000007482.JanuaryCTWannLSCalkinsHChenLYCigarroaJEClevelandJCJrEllinorPTEzekowitzMDFieldMEFurieKL
2019 AHA/ACC/HRS focused update of the 2014 AHA/ACC/HRS guideline for the management of patients with atrial fibrillation: a report of the American College of Cardiology/American Heart Association Task Force on Clinical Practice Guidelines and the Heart Rhythm Society in Collaboration with the society of thoracic surgeons.
Circulation. 2019;140:e125–e151. doi: 10.1161/CIR.00000000000006653068604110.1161/CIR.00000000000006653.PizzettiFTurazzaFMFranzosiMGBarleraSLeddaAMaggioniAPSantoroLTognoniG; GISSI-3 Investigators. Incidence and prognostic significance of atrial fibrillation in acute myocardial infarction: the GISSI-3 data.
Heart. 2001;86:527–532. doi: 10.1136/heart.86.5.5271160254510.1136/heart.86.5.527PMC17299694.GibsonCMHolmesDMikdadiGPresserDWohnsDYeeMKKaplanACiuffoAEberlyALIIIIteldB
Implantable cardiac alert system for early recognition of ST-segment elevation myocardial infarction.
J Am Coll Cardiol. 2019;73:1919–1927. doi: 10.1016/j.jacc.2019.01.0143084202810.1016/j.jacc.2019.01.0145.HolmesDRJrKrucoffMWMullinCMikdadiGPresserDWohnsDKaplanACiuffoAEberlyALIIIIteldB
Implanted monitor alerting to reduce treatment delay in patients with acute coronary syndrome events.
J Am Coll Cardiol. 2019;74:2047–2055. doi: 10.1016/j.jacc.2019.07.0843162376210.1016/j.jacc.2019.07.0846.MoserDKKimbleLPAlbertsMJAlonzoACroftJBDracupKEvensonKRGoASHandMMKothariRU
Reducing delay in seeking treatment by patients with acute coronary syndrome and stroke: a scientific statement from the American Heart Association Council on cardiovascular nursing and stroke council.
Circulation. 2006;114:168–182. doi: 10.1161/CIRCULATIONAHA.106.1760401680145810.1161/CIRCULATIONAHA.106.1760407.Van HeuverswynFDe BuyzereMCoemanMDe PooterJDriegheBDuytschaeverMGevaertSKayaertPVandekerckhoveYVoetJ
Feasibility and performance of a device for automatic self-detection of symptomatic acute coronary artery occlusion in outpatients with coronary artery disease: a multicentre observational study.
Lancet Digit Health. 2019;1:e90–e99. doi: 10.1016/S2589-7500(19)30026-33332323310.1016/S2589-7500(19)30026-38.AvilaCO
Novel use of apple watch 4 to obtain 3-lead electrocardiogram and detect cardiac ischemia.
Perm J. 2019;23:19-025
doi: 10.7812/TPP/19-02510.7812/TPP/19-025PMC6636475313147349.ClemmensenPLoumann-NielsenSSejerstenM
Telemedicine fighting acute coronary syndromes.
J Electrocardiol. 2010;43:615–618. doi: 10.1016/j.jelectrocard.2010.06.0122083281510.1016/j.jelectrocard.2010.06.01210.Sanchez-RossMOghlakianGMaherJPatelBMazzaVHomDDhruvaVLangleyDPalmaroJAhmedS
The STAT-MI (ST-segment analysis using wireless technology in acute myocardial infarction) trial improves outcomes.
JACC Cardiovasc Interv. 2011;4:222–227. doi: 10.1016/j.jcin.2010.11.0072134946210.1016/j.jcin.2010.11.00711.HorwitzLIMoriartyJPChenCFogertyRLBrewsterUCKanadeSZiaeianBJenqGYKrumholzHM
Quality of discharge practices and patient understanding at an academic medical center.
JAMA Intern Med. 2013;173:1715–1722. doi: 10.1001/jamainternmed.2013.93182395885110.1001/jamainternmed.2013.9318PMC383687112.ZiaeianBAraujoKLVan NessPHHorwitzLI
Medication reconciliation accuracy and patient understanding of intended medication changes on hospital discharge.
J Gen Intern Med. 2012;27:1513–1520. doi: 10.1007/s11606-012-2168-42279820010.1007/s11606-012-2168-4PMC347581613.VollmerWMOwen-SmithAATomJOLawsRDitmerDGSmithDHWaterburyACSchneiderJLYoneharaCHWilliamsA
Improving adherence to cardiovascular disease medications with information technology.
Am J Manag Care. 2014;2011 Spec No. 17SP502–SP510.25811824PMC635817614.ChowCKRedfernJHillisGSThakkarJSantoKHackettMLJanSGravesNde KeizerLBarryT
Effect of lifestyle-focused text messaging on risk factor modification in patients with coronary heart disease: a randomized clinical trial.
JAMA. 2015;314:1255–1263. doi: 10.1001/jama.2015.109452639384810.1001/jama.2015.1094515.UnalEGiakoumidakisKKhanEPatelarouE
Mobile phone text messaging for improving secondary prevention in cardiovascular diseases: a systematic review.
Heart Lung. 2018;47:351–359. doi: 10.1016/j.hrtlng.2018.05.0092980329710.1016/j.hrtlng.2018.05.00916.Shariful IslamSMFarmerAJBobrowKMaddisonRWhittakerRPfaeffli DaleLALechnerALearSEapenZNiessenLW
Mobile phone text-messaging interventions aimed to prevent cardiovascular diseases (Text2PreventCVD): systematic review and individual patient data meta-analysis.
Open Heart. 2019;6:e001017
doi: 10.1136/openhrt-2019-0010173167338110.1136/openhrt-2019-001017PMC680299917.ParkLGBeattyAStaffordZWhooleyMA
Mobile phone interventions for the secondary prevention of cardiovascular disease.
Prog Cardiovasc Dis. 2016;58:639–650. doi: 10.1016/j.pcad.2016.03.0022700124510.1016/j.pcad.2016.03.002PMC490482718.RitcheyMDMareshSMcNeelyJShafferTJacksonSLKeteyianSJBrawnerCAWhooleyMAChangTStolpH
Tracking cardiac rehabilitation participation and completion among medicare beneficiaries to inform the efforts of a national initiative.
Circ Cardiovasc Qual Outcomes. 2020;13:e005902
doi: 10.1161/CIRCOUTCOMES.119.0059023193161510.1161/CIRCOUTCOMES.119.005902PMC809157319.ZwislerADNortonRJDeanSGDalalHTangLHWinghamJTaylorRS
Home-based cardiac rehabilitation for people with heart failure: a systematic review and meta-analysis.
Int J Cardiol. 2016;221:963–969. doi: 10.1016/j.ijcard.2016.06.2072744147610.1016/j.ijcard.2016.06.20720.VarnfieldMKarunanithiMLeeCKHoneymanEArnoldDDingHSmithCWaltersDL
Smartphone-based home care model improved use of cardiac rehabilitation in postmyocardial infarction patients: results from a randomised controlled trial.
Heart. 2014;100:1770–1779. doi: 10.1136/heartjnl-2014-3057832497308310.1136/heartjnl-2014-30578321.BenjaminEJBlahaMJChiuveSECushmanMDasSRDeoRde FerrantiSDFloydJFornageMGillespieC; American Heart Association Statistics Committee and Stroke Statistics Subcommittee. Heart disease and stroke statistics-2017 update: a report from the American Heart Association.
Circulation. 2017;135:e146–e603. doi: 10.1161/CIR.00000000000004852812288510.1161/CIR.0000000000000485PMC540816022.AlbertCEstepJD
Economic impact of chronic heart failure management in today’s cost-conscious environment.
Card Electrophysiol Clin. 2019;11:1–9. doi: 10.1016/j.ccep.2018.11.0023071784110.1016/j.ccep.2018.11.00223.KoehlerFWinklerSSchieberMSechtemUStanglKBöhmMBollHBaumannGHonoldMKoehlerK; Telemedical Interventional Monitoring in Heart Failure Investigators. Impact of remote telemedical management on mortality and hospitalizations in ambulatory patients with chronic heart failure: the telemedical interventional monitoring in heart failure study.
Circulation. 2011;123:1873–1880. doi: 10.1161/CIRCULATIONAHA.111.0184732144488310.1161/CIRCULATIONAHA.111.01847324.ChaudhrySIMatteraJACurtisJPSpertusJAHerrinJLinZPhillipsCOHodshonBVCooperLSKrumholzHM
Telemonitoring in patients with heart failure.
N Engl J Med. 2010;363:2301–2309. doi: 10.1056/NEJMoa10100292108083510.1056/NEJMoa1010029PMC323739425.OngMKRomanoPSEdgingtonSAronowHUAuerbachADBlackJTDe MarcoTEscarceJJEvangelistaLSHannaB; Better Effectiveness After Transition–Heart Failure (BEAT-HF) Research Group. Effectiveness of remote patient monitoring after discharge of hospitalized patients with heart failure: the better effectiveness after transition – heart failure (BEAT-HF) randomized clinical trial.
JAMA Intern Med. 2016;176:310–318. doi: 10.1001/jamainternmed.2015.77122685738310.1001/jamainternmed.2015.7712PMC482770126.TakahashiPYPecinaJLUpatisingBChaudhryRShahNDVan HoutenHChaSCroghanINaessensJMHansonGJ
A randomized controlled trial of telemonitoring in older adults with multiple health issues to prevent hospitalizations and emergency department visits.
Arch Intern Med. 2012;172:773–779. doi: 10.1001/archinternmed.2012.2562250769610.1001/archinternmed.2012.256PMC391420027.BoyneJJVrijhoefHJCrijnsHJDe WeerdGKragtenJGorgelsAP; TEHAF Investigators. Tailored telemonitoring in patients with heart failure: results of a multicentre randomized controlled trial.
Eur J Heart Fail. 2012;14:791–801. doi: 10.1093/eurjhf/hfs0582258831910.1093/eurjhf/hfs05828.StehlikJSchmalfussCBozkurtBNativi-NicolauJWohlfahrtPWegerichSRoseKRayRSchofieldRDeswalA
Continuous wearable monitoring analytics predict heart failure hospitalization: the LINK-HF multicenter study.
Circ Heart Fail. 2020;13:e006513
doi: 10.1161/CIRCHEARTFAILURE.119.0065133209350610.1161/CIRCHEARTFAILURE.119.00651329.DickinsonMGAllenLAAlbertNADiSalvoTEwaldGAVestARWhellanDJZileMRGivertzMM
Remote monitoring of patients with heart failure: a white paper from the Heart Failure Society of America Scientific Statements Committee.
J Card Fail. 2018;24:682–694. doi: 10.1016/j.cardfail.2018.08.0113030824210.1016/j.cardfail.2018.08.01130.OnoMVarmaN
Remote monitoring to improve long-term prognosis in heart failure patients with implantable cardioverter-defibrillators.
Expert Rev Med Devices. 2017;14:335–342. doi: 10.1080/17434440.2017.13064382829995610.1080/17434440.2017.130643831.KoehlerFKoehlerKDeckwartOPrescherSWegscheiderKKirwanBAWinklerSVettorazziEBruchLOeffM
Efficacy of telemedical interventional management in patients with heart failure (TIM-HF2): a randomised, controlled, parallel-group, unmasked trial.
Lancet. 2018;392:1047–1057. doi: 10.1016/S0140-6736(18)31880-43015398510.1016/S0140-6736(18)31880-432.AbrahamWTStevensonLWBourgeRCLindenfeldJABaumanJGAdamsonPB; CHAMPION Trial Study Group. Sustained efficacy of pulmonary artery pressure to guide adjustment of chronic heart failure therapy: complete follow-up results from the CHAMPION randomised trial.
Lancet. 2016;387:453–461. doi: 10.1016/S0140-6736(15)00723-02656024910.1016/S0140-6736(15)00723-033.CarboAGuptaMTamarizLPalacioALevisSNemethZDangS
Mobile technologies for managing heart failure: a systematic review and meta-analysis [published online April 2, 2018].
Telemed JE Health. doi: 10.1089/tmj.2017.0269. https://www.liebertpub.com/doi/10.1089/tmj.2017.026910.1089/tmj.2017.02692960843034.DesaiASBhimarajABharmiRJermynRBhattKShavelleDRedfieldMMHullRPelzelJDavisK
Ambulatory hemodynamic monitoring reduces heart failure hospitalizations in “real-world” clinical practice.
J Am Coll Cardiol. 2017;69:2357–2365. doi: 10.1016/j.jacc.2017.03.0092833075110.1016/j.jacc.2017.03.00935.CipressoPSerinoSVillan,iDRepettoCSellittiLAlbaniAMauroAGaggioliARivaG
Is your phone so smart to affect your state? An exploratory study based on psychophysiological measures.
Neurocomputing. 2012;84:23–30.36.MolinaroNMassaroniCLo PrestiDSaccomandiPDi TomasoGZolloLPeregoPAndreoniGSchenaE
Wearable textile based on silver plated knitted sensor for respiratory rate monitoring.
Annu Int Conf IEEE Eng Med Biol Soc. 2018;2018:2865–2868. doi: 10.1109/EMBC.2018.85129583044099910.1109/EMBC.2018.851295837.ChaudhrySIMatteraJACurtisJPSpertusJAHerrinJLinZPhillipsCOHodshonBVCooperLSKrumholzHM
Telemonitoring in patients with heart failure.
N Engl J Med. 2010;363:2301–2309. doi: 10.1056/NEJMoa10100292108083510.1056/NEJMoa1010029PMC323739438.HamiltonSJMillsBBirchEMThompsonSC
Smartphones in the secondary prevention of cardiovascular disease: a systematic review.
BMC Cardiovasc Disord. 2018;18:25
doi: 10.1186/s12872-018-0764-x2941568010.1186/s12872-018-0764-xPMC580399839.PiepoliMFConraadsVCorràUDicksteinKFrancisDPJaarsmaTMcMurrayJPieskeBPiotrowiczESchmidJP
Exercise training in heart failure: from theory to practice. A consensus document of the Heart Failure Association and the European Association for Cardiovascular Prevention and Rehabilitation.
Eur J Heart Fail. 2011;13:347–357. doi: 10.1093/eurjhf/hfr0172143636010.1093/eurjhf/hfr01740.PonikowskiPVoorsAAAnkerSDBuenoHClelandJGFCoatsAJSFalkVGonzález-JuanateyJRHarjolaVPJankowskaEA; ESC Scientific Document Group. 2016 ESC guidelines for the diagnosis and treatment of acute and chronic heart failure: the task force for the diagnosis and treatment of acute and chronic heart failure of the European Society of Cardiology (ESC)Developed with the special contribution of the Heart Failure Association (HFA) of the ESC.
Eur Heart J. 2016;37:2129–2200. doi: 10.1093/eurheartj/ehw1282720681910.1093/eurheartj/ehw12841.PiotrowiczEPiepoliMFJaarsmaTLambrinouECoatsAJSchmidJPCorràUAgostoniPDicksteinKSeferovićPM
Telerehabilitation in heart failure patients: the evidence and the pitfalls.
Int J Cardiol. 2016;220:408–413. doi: 10.1016/j.ijcard.2016.06.2772739096310.1016/j.ijcard.2016.06.27742.SmartNHaluskaBJeffriessLMarwickTH
Predictors of a sustained response to exercise training in patients with chronic heart failure: a telemonitoring study.
Am Heart J. 2005;150:1240–1247. doi: 10.1016/j.ahj.2005.01.0351633826610.1016/j.ahj.2005.01.03543.KouidiEFarmakiotisAKouidisNDeligiannisA
Transtelephonic electrocardiographic monitoring of an outpatient cardiac rehabilitation programme.
Clin Rehabil. 2006;20:1100–1104. doi: 10.1177/02692155060712561714852210.1177/026921550607125644.PiotrowiczEZielińskiTBodalskiRRywikTDobraszkiewicz-WasilewskaBSobieszczańska-MałekMStepnowskaMPrzybylskiABrowarekASzumowskiŁ
Home-based telemonitored Nordic walking training is well accepted, safe, effective and has high adherence among heart failure patients, including those with cardiovascular implantable electronic devices: a randomised controlled study.
Eur J Prev Cardiol. 2015;22:1368–1377. doi: 10.1177/20474873145515372526126810.1177/204748731455153745.AdesPAPashkowFJFletcherGPinaILZohmanLRNestorJR
A controlled trial of cardiac rehabilitation in the home setting using electrocardiographic and voice transtelephonic monitoring.
Am Heart J. 2000;139:543–548. doi: 10.1016/s0002-8703(00)90100-51068927110.1016/s0002-8703(00)90100-546.PiotrowiczEBuchnerTPiotrowskiWPiotrowiczR
Influence of home-based telemonitored Nordic walking training on autonomic nervous system balance in heart failure patients.
Arch Med Sci. 2015;11:1205–1212. doi: 10.5114/aoms.2015.563462678808110.5114/aoms.2015.56346PMC469705447.PiotrowiczEPiotrowskiWPiotrowiczR
Positive effects of the reversion of depression on the sympathovagal balance after telerehabilitation in heart failure patients.
Ann Noninvasive Electrocardiol. 2016;21:358–368. doi: 10.1111/anec.123202652469910.1111/anec.12320PMC693159648.PiotrowiczEStepnowskaMLeszczyńska-IwanickaKPiotrowskaDKowalskaMTylkaJPiotrowskiWPiotrowiczR
Quality of life in heart failure patients undergoing home-based telerehabilitation versus outpatient rehabilitation–a randomized controlled study.
Eur J Cardiovasc Nurs. 2015;14:256–263. doi: 10.1177/14745151145370232484930410.1177/147451511453702349.PiotrowiczEPencinaMJOpolskiGZarebaWBanachMKowalikIOrzechowskiPSzalewskaDPlutaSGlówczynskaR
Effects of a 9-week hybrid comprehensive telerehabilitation program on long-term outcomes in patients with heart failure: the telerehabilitation in heart failure patients (TELEREH-HF) randomized clinical trial.
JAMA Cardiol. 2020;5:300–308. doi: 10.1001/jamacardio.2019.50063173470110.1001/jamacardio.2019.5006PMC686532550.PiotrowiczEPiotrowiczROpolskiGPencinaMBanachMZarębaW
Hybrid comprehensive telerehabilitation in heart failure patients (TELEREH-HF): a randomized, multicenter, prospective, open-label, parallel group controlled trial-Study design and description of the intervention.
Am Heart J. 2019;217:148–158. doi: 10.1016/j.ahj.2019.08.0153165494410.1016/j.ahj.2019.08.01551.ThomasRJBeattyALBeckieTMBrewerLCBrownTMFormanDEFranklinBAKeteyianSJKitzmanDWRegensteinerJG
Home-based cardiac rehabilitation: a scientific statement from the American Association of Cardiovascular and Pulmonary Rehabilitation, the American Heart Association, and the American College of Cardiology.
Circulation. 2019;140:e69–e89. doi: 10.1161/CIR.00000000000006633108226610.1161/CIR.000000000000066352.BalakumarPMaung-UKJagadeeshG
Prevalence and prevention of cardiovascular disease and diabetes mellitus.
Pharmacol Res. 2016;113pt A600–609. doi: 10.1016/j.phrs.2016.09.0402769764710.1016/j.phrs.2016.09.04053.DonnellanEAagaardPKanjMJaberWElshazlyMHoosienMBaranowskiBHusseinASalibaWWazniO
Association between pre-ablation glycemic control and outcomes among patients with diabetes undergoing atrial fibrillation ablation.
JACC Clin Electrophysiol. 2019;5:897–903. doi: 10.1016/j.jacep.2019.05.0183143928910.1016/j.jacep.2019.05.01854.GoudisCAKorantzopoulosPNtalasIVKallergisEMLiuTKetikoglouDG
Diabetes mellitus and atrial fibrillation: pathophysiological mechanisms and potential upstream therapies.
Int J Cardiol. 2015;184:617–622. doi: 10.1016/j.ijcard.2015.03.0522577084110.1016/j.ijcard.2015.03.05255.WangAGreenJBHalperinJLPicciniJPSr.
Atrial fibrillation and diabetes mellitus: JACC review topic of the week.
J Am Coll Cardiol. 2019;74:1107–1115. doi: 10.1016/j.jacc.2019.07.0203143922010.1016/j.jacc.2019.07.02056.WilkinsonMJZadourianATaubPR
Heart failure and diabetes mellitus: defining the problem and exploring the interrelationship.
Am J Cardiol. 2019;124suppl 1S3–S11. doi: 10.1016/j.amjcard.2019.10.0243174143810.1016/j.amjcard.2019.10.02457.WingerterRSteigerNBurrowsAEstesNAMIII.
Impact of lifestyle modification on atrial fibrillation.
Am J Cardiol. 2020;125:289–297. doi: 10.1016/j.amjcard.2019.10.0183176114710.1016/j.amjcard.2019.10.01858.CosentinoFGrantPJAboyansVBaileyCJCerielloADelgadoVFedericiMFilippatosGGrobbeeDEHansenTB; ESC Scientific Document Group. 2019 ESC guidelines on diabetes, pre-diabetes, and cardiovascular diseases developed in collaboration with the EASD.
Eur Heart J. 2020;41:255–323. doi: 10.1093/eurheartj/ehz4863149785410.1093/eurheartj/ehz48659.ChaoTFLeuHBHuangCCChenJWChanWLLinSJChenSA
Thiazolidinediones can prevent new onset atrial fibrillation in patients with non-insulin dependent diabetes.
Int J Cardiol. 2012;156:199–202. doi: 10.1016/j.ijcard.2011.08.0812193031510.1016/j.ijcard.2011.08.08160.ChangSHWuLSChiouMJLiuJRYuKHKuoCFWenMSChenWJYehYHSeeLC
Association of metformin with lower atrial fibrillation risk among patients with type 2 diabetes mellitus: a population-based dynamic cohort and in vitro studies.
Cardiovasc Diabetol. 2014;13:123
doi: 10.1186/s12933-014-0123-x2510607910.1186/s12933-014-0123-xPMC414927361.GuJLiuXWangXShiHTanHZhouLGuJJiangWWangY
Beneficial effect of pioglitazone on the outcome of catheter ablation in patients with paroxysmal atrial fibrillation and type 2 diabetes mellitus.
Europace. 2011;13:1256–1261. doi: 10.1093/europace/eur1312155147910.1093/europace/eur13162.OtakeHSuzukiHHondaTMaruyamaY
Influences of autonomic nervous system on atrial arrhythmogenic substrates and the incidence of atrial fibrillation in diabetic heart.
Int Heart J. 2009;50:627–641. doi: 10.1536/ihj.50.6271980921110.1536/ihj.50.62763.AndrèsEMeyerLZulfiqarAAHajjamMTalhaSBahougneTErvéSHajjamJDoucetJJeandidierN
Telemonitoring in diabetes: evolution of concepts and technologies, with a focus on results of the more recent studies.
J Med Life. 2019;12:203–214. doi: 10.25122/jml-2019-00063166681810.25122/jml-2019-0006PMC681489064.GarabedianLFRoss-DegnanDWharamJF
Mobile phone and smartphone technologies for diabetes care and self-management.
Curr Diab Rep. 2015;15:109
doi: 10.1007/s11892-015-0680-82645838010.1007/s11892-015-0680-8PMC652533165.FokkertMJvan DijkPREdensMAAbbesSde JongDSlingerlandRJBiloHJ
Performance of the FreeStyle Libre Flash glucose monitoring system in patients with type 1 and 2 diabetes mellitus.
BMJ Open Diabetes Res Care. 2017;5:e000320
doi: 10.1136/bmjdrc-2016-00032010.1136/bmjdrc-2016-000320PMC53169122824344966.FlemingGAPetrieJRBergenstalRMHollRWPetersALHeinemannL
Diabetes digital app technology: benefits, challenges, and recommendations. A consensus report by the European Association for the Study of Diabetes (EASD) and the American Diabetes Association (ADA) Diabetes Technology Working Group.
Diabetes Care. 2020;43:250–260. doi: 10.2337/dci19-00623180664910.2337/dci19-006267.VeazieSWinchellKGilbertJPaynterRIvlevIEdenKBNussbaumKWeiskopfNGuiseJMHelfandM
Rapid evidence review of mobile applications for self-management of diabetes.
J Gen Intern Med. 2018;33:1167–1176. doi: 10.1007/s11606-018-4410-12974078610.1007/s11606-018-4410-1PMC602568068.LiangXWangQYangXCaoJChenJMoXHuangJWangLGuD
Effect of mobile phone intervention for diabetes on glycaemic control: a meta-analysis.
Diabet Med. 2011;28:455–463. doi: 10.1111/j.1464-5491.2010.03180.x2139206610.1111/j.1464-5491.2010.03180.x69.PillayJArmstrongMJButaliaSDonovanLESigalRJChordiyaPDhakalSVandermeerBHartlingLNusplM
Behavioral programs for type 1 diabetes mellitus: a systematic review and meta-analysis.
Ann Intern Med. 2015;163:836–847. doi: 10.7326/M15-13992641402010.7326/M15-139970.SaffariMGhanizadehGKoenigHG
Health education via mobile text messaging for glycemic control in adults with type 2 diabetes: a systematic review and meta-analysis.
Prim Care Diabetes. 2014;8:275–285. doi: 10.1016/j.pcd.2014.03.0042479358910.1016/j.pcd.2014.03.00471.FokkertMvan DijkPEdensMBarentsEMollemaJSlingerlandRGansRBiloH
Improved well-being and decreased disease burden after 1-year use of flash glucose monitoring (FLARE-NL4).
BMJ Open Diabetes Res Care. 2019;7:e000809
doi: 10.1136/bmjdrc-2019-00080910.1136/bmjdrc-2019-000809PMC69041653187513372.AgarwalPMukerjiGDesveauxLIversNMBhattacharyyaOHenselJMShawJBouckZJamiesonTOnabajoN
Mobile app for improved self-management of type 2 diabetes: multicenter pragmatic randomized controlled trial.
JMIR Mhealth Uhealth. 2019;7:e10321
doi: 10.2196/103213063297210.2196/10321PMC632989673.QuinnCCShardellMDTerrinMLBarrEABallewSHGruber-BaldiniAL
Cluster-randomized trial of a mobile phone personalized behavioral intervention for blood glucose control.
Diabetes Care. 2011;34:1934–1942. doi: 10.2337/dc11-03662178863210.2337/dc11-0366PMC316130574.PalKEastwoodSVMichieSFarmerABarnardMLPeacockRWoodBEdwardsPMurrayE
Computer-based interventions to improve self-management in adults with type 2 diabetes: a systematic review and meta-analysis.
Diabetes Care. 2014;37:1759–1766. doi: 10.2337/dc13-13862485515810.2337/dc13-138675.WhaleyCMBollykyJBLuWPainterSSchneiderJZhaoZHeXJohnsonJMeadowsES
Reduced medical spending associated with increased use of a remote diabetes management program and lower mean blood glucose values.
J Med Econ. 2019;22:869–877. doi: 10.1080/13696998.2019.16094833101239210.1080/13696998.2019.160948376.HuxleyRRLopezFLFolsomARAgarwalSKLoehrLRSolimanEZMaclehoseRKonetySAlonsoA
Absolute and attributable risks of atrial fibrillation in relation to optimal and borderline risk factors: the Atherosclerosis Risk in Communities (ARIC) study.
Circulation. 2011;123:1501–1508. doi: 10.1161/CIRCULATIONAHA.110.0090352144487910.1161/CIRCULATIONAHA.110.009035PMC318149877.BosworthHBPowersBJOlsenMKMcCantFGrubberJSmithVGentryPWRoseCVan HoutvenCWangV
Home blood pressure management and improved blood pressure control: results from a randomized controlled trial.
Arch Intern Med. 2011;171:1173–1180. doi: 10.1001/archinternmed.2011.2762174701310.1001/archinternmed.2011.27678.KimYNShinDGParkSLeeCH
Randomized clinical trial to assess the effectiveness of remote patient monitoring and physician care in reducing office blood pressure.
Hypertens Res. 2015;38:491–497. doi: 10.1038/hr.2015.322578704110.1038/hr.2015.3279.McManusRJMantJBrayEPHolderRJonesMIGreenfieldSKaambwaBBantingMBryanSLittleP
Telemonitoring and self-management in the control of hypertension (TASMINH2): a randomised controlled trial.
Lancet. 2010;376:163–172. doi: 10.1016/S0140-6736(10)60964-62061944810.1016/S0140-6736(10)60964-680.DuanYXieZDongFWuZLinZSunNXuJ
Effectiveness of home blood pressure telemonitoring: a systematic review and meta-analysis of randomised controlled studies.
J Hum Hypertens. 2017;31:427–437. doi: 10.1038/jhh.2016.992833250610.1038/jhh.2016.9981.TuckerKLSheppardJPStevensRBosworthHBBoveABrayEPEarleKGeorgeJGodwinMGreenBB
Self-monitoring of blood pressure in hypertension: a systematic review and individual patient data meta-analysis.
PLoS Med. 2017;14:e1002389
doi: 10.1371/journal.pmed.10023892892657310.1371/journal.pmed.1002389PMC560496582.McManusRJMantJFranssenMNicklessASchwartzCHodgkinsonJBradburnPFarmerAGrantSGreenfieldSM; TASMINH4 Investigators. Efficacy of self-monitored blood pressure, with or without telemonitoring, for titration of antihypertensive medication (TASMINH4): an unmasked randomised controlled trial.
Lancet. 2018;391:949–959. doi: 10.1016/S0140-6736(18)30309-X2949987310.1016/S0140-6736(18)30309-XPMC585446383.MonahanMJowettSNicklessAFranssenMGrantSGreenfieldSHobbsFDRHodgkinsonJMantJMcManusRJ
Cost-effectiveness of telemonitoring and self-monitoring of blood pressure for antihypertensive titration in primary care (TASMINH4).
Hypertension. 2019;73:1231–1239. doi: 10.1161/HYPERTENSIONAHA.118.124153106719010.1161/HYPERTENSIONAHA.118.12415PMC651040584.WilliamsBManciaGSpieringWAgabiti RoseiEAziziMBurnierMClementDLCocaAde SimoneGDominiczakA; ESC Scientific Document Group. 2018 ESC/ESH guidelines for the management of arterial hypertension.
Eur Heart J. 2018;39:3021–3104. doi: 10.1093/eurheartj/ehy3393016551610.1093/eurheartj/ehy33985.DaghlasIDashtiHSLaneJAragamKGRutterMKSaxenaRVetterC
Sleep duration and myocardial infarction.
J Am Coll Cardiol. 2019;74:1304–1314. doi: 10.1016/j.jacc.2019.07.0223148826710.1016/j.jacc.2019.07.022PMC678501186.HirshkowitzMWhitonKAlbertSMAlessiCBruniODonCarlosLHazenNHermanJAdams HillardPJKatzES
National Sleep Foundation’s updated sleep duration recommendations: final report.
Sleep Health. 2015;1:233–243. doi: 10.1016/j.sleh.2015.10.0042907339810.1016/j.sleh.2015.10.00487.MehraRBenjaminEJShaharEGottliebDJNawabitRKirchnerHLSahadevanJRedlineS; Sleep Heart Health Study. Association of nocturnal arrhythmias with sleep-disordered breathing: the Sleep Heart Health Study.
Am J Respir Crit Care Med. 2006;173:910–916. doi: 10.1164/rccm.200509-1442OC1642444310.1164/rccm.200509-1442OCPMC266290988.MayAMBlackwellTStonePHStoneKLCawthonPMSauerWHVarosyPDRedlineSMehraR; MrOS Sleep (Outcomes of Sleep Disorders in Older Men) Study Group. Central sleep-disordered breathing predicts incident atrial fibrillation in older men.
Am J Respir Crit Care Med. 2016;193:783–791. doi: 10.1164/rccm.201508-1523OC2659538010.1164/rccm.201508-1523OCPMC482493289.MayAMVan WagonerDRMehraR
OSA and cardiac arrhythmogenesis: mechanistic insights.
Chest. 2017;151:225–241. doi: 10.1016/j.chest.2016.09.0142769359410.1016/j.chest.2016.09.014PMC598964390.ColtenHRAltevogtBM; Institute of Medicine (US) Committee on Sleep Medicine and Research. Sleep Disorders and Sleep Deprivation: an Unmet Public Health Problem. 2006
National Academies Press (US)
3, Extent and Health Consequences of Chronic Sleep Loss and Sleep Disorders. http://www.ncbi.nlm.nih.gov/books/NBK19961/2066943891.BurgessHJTrinderJKimYLukeD
Sleep and circadian influences on cardiac autonomic nervous system activity.
Am J Physiol. 1997;273:H1761–H1768. doi: 10.1152/ajpheart.1997.273.4.H1761936224110.1152/ajpheart.1997.273.4.H176192.QureshiWTNasirUBAlqalyoobiSO’NealWTMawriSSabbaghSSolimanEZAl-MallahMH
Meta-analysis of continuous positive airway pressure as a therapy of atrial fibrillation in obstructive sleep apnea.
Am J Cardiol. 2015;116:1767–1773. doi: 10.1016/j.amjcard.2015.08.0462648218210.1016/j.amjcard.2015.08.04693.YoussefIKamranHYacoubMPatelNGoulbourneCKumarSKaneJHoffnerHSalifuMMcFarlaneSI
Obstructive sleep apnea as a risk factor for atrial fibrillation: a meta-analysis.
J Sleep Disord Ther. 2018;7:282
doi: 10.4172/2167-0277.10002822965790310.4172/2167-0277.1000282PMC589840194.KhoslaSDeakMCGaultDGoldsteinCAHwangDKwonYO’HearnDSchutte-RodinSYurcheshenMRosenIM; American Academy of Sleep Medicine Board of Directors. Consumer sleep technology: an American Academy of Sleep Medicine position statement.
J Clin Sleep Med. 2018;14:877–880. doi: 10.5664/jcsm.71282973499710.5664/jcsm.7128PMC594044095.MantuaJGravelNSpencerRM
Reliability of sleep measures from four personal health monitoring devices compared to research-based actigraphy and polysomnography.
Sensors (Basel). 2016;16:646
doi: 10.3390/s1605064610.3390/s16050646PMC48833372716411096.SelvarajNNarasimhanR
Automated prediction of the apnea-hypopnea index using a wireless patch sensor.
Annu Int Conf IEEE Eng Med Biol Soc. 2014;2014:1897–1900. doi: 10.1109/EMBC.2014.69439812557034910.1109/EMBC.2014.694398197.HuangTMarianiSRedlineS
Sleep irregularity and risk of cardiovascular events: the multi-ethnic study of atherosclerosis.
J Am Coll Cardiol. 2020;75:991–999. doi: 10.1016/j.jacc.2019.12.0543213897410.1016/j.jacc.2019.12.054PMC723795598.BerryhillSMortonCJDeanABerryhillAProvencio-DeanNPatelSIEstepLCombsDMashaqiSGeraldLB
Effect of wearables on sleep in healthy individuals: a randomized crossover trial and validation study.
J Clin Sleep Med. 2020;16:775–783. doi: 10.5664/jcsm.83563204396110.5664/jcsm.8356PMC784981699.EverettBMConenDBuringJEMoorthyMVLeeIMAlbertCM
Physical activity and the risk of incident atrial fibrillation in women.
Circ Cardiovasc Qual Outcomes. 2011;4:321–327. doi: 10.1161/CIRCOUTCOMES.110.9514422148709210.1161/CIRCOUTCOMES.110.951442PMC3097307100.MozaffarianDFurbergCDPsatyBMSiscovickD
Physical activity and incidence of atrial fibrillation in older adults: the cardiovascular health study.
Circulation. 2008;118:800–807. doi: 10.1161/CIRCULATIONAHA.108.7856261867876810.1161/CIRCULATIONAHA.108.785626PMC3133958101.PiercyKLTroianoRPBallardRMCarlsonSAFultonJEGaluskaDAGeorgeSMOlsonRD
The physical activity guidelines for Americans.
JAMA. 2018;320:2020–2028. doi: 10.1001/jama.2018.148543041847110.1001/jama.2018.14854PMC9582631102.KramerDBMitchellSLJonesPWNormandS-LHayesDLReynoldsMR
Patient activity and survival following implantable cardioverter-defibrillator implantation: the ALTITUDE activity study.
J Am Heart Assoc. 2015;4:e001775
doi: 10.1161/JAHA.115.0017752597990210.1161/JAHA.115.001775PMC4599410103.IQUVIA Institute. The growing value of digital health: evidence and impact on human health and the healthcare system [Internet].
11
07, 2017
Accessed January 31, 2020. https://www.iqvia.com/insights/the-iqvia-institute/reports/the-growing-value-of-digital-health104.McConnellMVTurakhiaMPHarringtonRAKingACAshleyEA
Mobile health advances in physical activity, fitness, and atrial fibrillation: moving hearts.
J Am Coll Cardiol. 2018;71:2691–2701. doi: 10.1016/j.jacc.2018.04.0302988013010.1016/j.jacc.2018.04.030105.FaselisCKokkinosPTsimploulisAPittarasAMyersJLavieCJKyritsiFLovicDKarasikPMooreH
Exercise capacity and atrial fibrillation risk in veterans: a cohort study.
Mayo Clin Proc. 2016;91:558–566. doi: 10.1016/j.mayocp.2016.03.0022706867010.1016/j.mayocp.2016.03.002106.PathakRKElliottAMiddeldorpMEMeredithMMehtaABMahajanRHendriksJMTwomeyDKalmanJMAbhayaratnaWP
Impact of CARDIOrespiratory FITness on arrhythmia recurrence in obese individuals with atrial fibrillation: the CARDIO-FIT study.
J Am Coll Cardiol. 2015;66:985–996. doi: 10.1016/j.jacc.2015.06.4882611340610.1016/j.jacc.2015.06.488107.RosenbergerMEBumanMPHaskellWLMcConnellMVCarstensenLL
Twenty-four hours of sleep, sedentary behavior, and physical activity with nine wearable devices.
Med Sci Sports Exerc. 2016;48:457–465. doi: 10.1249/MSS.00000000000007782648495310.1249/MSS.0000000000000778PMC4760880108.SullivanANLachmanME
Behavior change with fitness technology in sedentary adults: a review of the evidence for increasing physical activity.
Front Public Health. 2016;4:289
doi: 10.3389/fpubh.2016.002892812399710.3389/fpubh.2016.00289PMC5225122109.JakicicJMDavisKKRogersRJKingWCMarcusMDHelselDRickmanADWahedASBelleSH
Effect of wearable technology combined with a lifestyle intervention on long-term weight loss: the IDEA randomized clinical trial.
JAMA. 2016;316:1161–1171. doi: 10.1001/jama.2016.128582765460210.1001/jama.2016.12858PMC5480209110.AbdullaJNielsenJR
Is the risk of atrial fibrillation higher in athletes than in the general population? A systematic review and meta-analysis.
Europace. 2009;11:1156–1159. doi: 10.1093/europace/eup1971963330510.1093/europace/eup197111.AndersenKFarahmandBAhlbomAHeldCLjunghallSMichaëlssonKSundströmJ
Risk of arrhythmias in 52 755 long-distance cross-country skiers: a cohort study.
Eur Heart J. 2013;34:3624–3631. doi: 10.1093/eurheartj/eht1882375633210.1093/eurheartj/eht188112.BrunettiNDDellegrottaglieGDi GiuseppeGLoprioreCLoiaconoTGardiniGPatrunoSDe gennaroLDi BiaseM
YOUng Football Italian amateur players Remote electrocardiogram Screening with Telemedicine (YOU FIRST) study: preliminary results.
Int J Cardiol. 2014;176:1257–1258. doi: 10.1016/j.ijcard.2014.07.1952512499710.1016/j.ijcard.2014.07.195113.OrchardJJNeubeckLOrchardJWPuranikRRajuHFreedmanBLa GercheASemsarianC
ECG-based cardiac screening programs: legal, ethical, and logistical considerations.
Heart Rhythm. 2019;16:1584–1591. doi: 10.1016/j.hrthm.2019.03.0253093033110.1016/j.hrthm.2019.03.025114.AroganamGManivannanNHarrisonD
Review on wearable technology sensors used in consumer sport applications.
Sensors (Basel). 2019;19:1983
doi: 10.3390/s1909198310.3390/s19091983PMC654027031035333115.LiRTKlingSRSalataMJCuppSASheehanJVoosJE
Wearable performance devices in sports medicine.
Sports Health. 2016;8:74–78. doi: 10.1177/19417381156169172673359410.1177/1941738115616917PMC4702159116.PeakeJMKerrGSullivanJP
A Critical review of consumer wearables, mobile applications, and equipment for providing biofeedback, monitoring stress, and sleep in physically active populations.
Front Physiol. 2018;9:743
doi: 10.3389/fphys.2018.007433000262910.3389/fphys.2018.00743PMC6031746117.PeartDJBalsalobre-FernándezCShawMP
Use of mobile applications to collect data in sport, health, and exercise science: a narrative review.
J Strength Cond Res. 2019;33:1167–1177. doi: 10.1519/JSC.00000000000023442917638410.1519/JSC.0000000000002344118.SeshadriDRLiRTVoosJERowbottomJRAlfesCMZormanCADrummondCK
Wearable sensors for monitoring the internal and external workload of the athlete.
NPJ Digit Med. 2019;2:71
doi: 10.1038/s41746-019-0149-23137250610.1038/s41746-019-0149-2PMC6662809119.CoppettiTBrauchlinAMügglerSAttinger-TollerATemplinCSchönrathFHellermannJLüscherTFBiaggiPWyssCA
Accuracy of smartphone apps for heart rate measurement.
Eur J Prev Cardiol. 2017;24:1287–1293. doi: 10.1177/20474873177020442846470010.1177/2047487317702044120.DobbsWCFedewaMVMacDonaldHVHolmesCJCiconeZSPlewsDJEscoMR
The accuracy of acquiring heart rate variability from portable devices: a systematic review and meta-analysis.
Sports Med. 2019;49:417–435. doi: 10.1007/s40279-019-01061-53070623410.1007/s40279-019-01061-5121.SinghNMoneghettiKJChristleJWHadleyDFroelicherVPlewsD
Heart rate variability: an old metric with new meaning in the era of Using mHealth technologies for Health and Exercise Training Guidance. Part two: prognosis and training.
Arrhythm Electrophysiol Rev. 2018;7:247–255. doi: 10.15420/aer.2018.30.23058831210.15420/aer.2018.30.2PMC6304793122.FolsomARYatsuyaHNettletonJALutseyPLCushmanMRosamondWD; ARIC Study Investigators. Community prevalence of ideal cardiovascular health, by the American Heart Association definition, and relationship with cardiovascular disease incidence.
J Am Coll Cardiol. 2011;57:1690–1696. doi: 10.1016/j.jacc.2010.11.0412149276710.1016/j.jacc.2010.11.041PMC3093047123.AbedHSWittertGALeongDPShiraziMGBahramiBMiddeldorpMELorimerMFLauDHAnticNABrooksAG
Effect of weight reduction and cardiometabolic risk factor management on symptom burden and severity in patients with atrial fibrillation: a randomized clinical trial.
JAMA. 2013;310:2050–2060. doi: 10.1001/jama.2013.2805212424093210.1001/jama.2013.280521124.DonnellanEWazniOMKanjMBaranowskiBCremerPHarbSMcCarthyCPMcEvoyJWElshazlyMBAagaardP
Association between pre-ablation bariatric surgery and atrial fibrillation recurrence in morbidly obese patients undergoing atrial fibrillation ablation.
Europace. 2019;21:1476–1483. doi: 10.1093/europace/euz1833130453210.1093/europace/euz183125.PathakRKMiddeldorpMEMeredithMMehtaABMahajanRWongCXTwomeyDElliottADKalmanJMAbhayaratnaWP
Long-term effect of goal-directed weight management in an atrial fibrillation cohort: a long-term follow-up study (LEGACY).
J Am Coll Cardiol. 2015;65:2159–2169. doi: 10.1016/j.jacc.2015.03.0022579236110.1016/j.jacc.2015.03.002126.El-GayarOTimsinaPNawarNEidW
Mobile applications for diabetes self-management: status and potential.
J Diabetes Sci Technol. 2013;7:247–262. doi: 10.1177/1932296813007001302343918310.1177/193229681300700130PMC3692239127.GriffithsCHarnackLPereiraMA
Assessment of the accuracy of nutrient calculations of five popular nutrition tracking applications.
Public Health Nutr. 2018;21:1495–1502. doi: 10.1017/S13689800180003932953477110.1017/S1368980018000393PMC10261454128.BousheyCJSpodenMZhuFMDelpEJKerrDA
New mobile methods for dietary assessment: review of image-assisted and image-based dietary assessment methods.
Proc Nutr Soc. 2017;76:283–294. doi: 10.1017/S00296651160029132793842510.1017/S0029665116002913129.SixBLSchapTEZhuFMMariappanABoschMDelpEJEbertDSKerrDABousheyCJ
Evidence-based development of a mobile telephone food record.
J Am Diet Assoc. 2010;110:74–79. doi: 10.1016/j.jada.2009.10.0102010283010.1016/j.jada.2009.10.010PMC3042797130.ZhuFQBoschMWooIKimSBousheyCJEbertDSDelpEJ
The use of mobile devices in aiding dietary assessment and evaluation.
IEEE J Sel Top Signal Process. 2010;4:756–766. doi: 10.1109/JSTSP.2010.20514712086226610.1109/JSTSP.2010.2051471PMC2941896131.FangSLiuCZhuFDelpEJBousheyCJ
Single-view food portion estimation based on geometric models.
ISM. 2015;2015:385–390. doi: 10.1109/ISM.2015.672767268210.1109/ISM.2015.67PMC5035274132.DounaviKTsoumaniO
Mobile health applications in weight management: a systematic literature review.
Am J Prev Med. 2019;56:894–903. doi: 10.1016/j.amepre.2018.12.0053100380110.1016/j.amepre.2018.12.005

## 5. Patient Self-Management–Integrated Chronic Care

Generally, structured management programs inclusive of intensive patient education may improve outcomes (Hendriks 2012, USPTF 2014, Angaran 2015).^1–3^ These may be facilitated by mHealth.

### 5.1. Patient Engagement

mHealth offers the opportunity to reach more patients more effectively. It may promote patient engagement through ease of access and wider dissemination to regions and communities who may not access health care through traditional modes due to cost, time, distance, embarrassment/stigma, marginalized groups, health inequities, etc (ventricular arrhythmia document).^4^ In this way, mHealth may facilitate information sharing and interaction between patients and HCPs without the need for an elaborate infrastructure (Chow 2016, Walsh 2014^5,6^; Figure 6). Apps may aid HCPs to explain the condition and treatment options, utilizing videos, avatars, and individualized risk scores, enabling greater patient understanding, and encouraging a 2-way exchange of information to achieve a concordant decision about treatment.

#### Patients’ Access to Their Own Health Data

A recent HRS statement advocates for transparent and secure access by patients to their digital data (Slotwiner 2019).^7^ This enables active participation and appropriate self-management. For instance, many patients with AF are interested in seeing their AF burden and physiological data, similarly to patients with hypertension tracking their BP or patients with diabetes tracking their glucose. Recent systematic reviews of technology-based patient-directed interventions for cardiovascular disease suggest that engaging elements include self-monitoring of symptoms and measurements, daily tracking of health behaviors, disease education, reminders, and interaction with HCPs (Coorey 2018, Gandhi 2017, Park 2016, Phaeffli 2016).^8–11^ In some cardiovascular conditions, self-management (without any HCP input) improved key outcomes (Hagglund 2015, Varnfield 2014).^12,13^

The model requires that patients assume responsibility and accountability for tracking conditions effectively and taking corrective measures. Possibly, this may be facilitated by data organization to present salient elements in a format comprehensible to the lay public. Active role of patients in decision-making regarding the choice of treatment has been underlined by AF clinical guidance documents. Patients with AF are encouraged to be involved in decision-taking through better understanding of their disease, which helps to improve communication between patients, their families, and doctors and improves patients’ adherence to prescribed therapy. Two applications in AF—one for patients and the other for health care providers—have been developed by CATCH ME Consortium (Characterizing Atrial Fibrillation by Translating Its Causes Into Health Modifiers in the Elderly) in collaboration with the European Society of Cardiology (Kotecha 2018),^14^ but these have yet to be formally tested. In China, Guo et al^15^ (2017) demonstrated that the mobile AF (mAFA) app, incorporating decision support, education, and patient engagement, significantly improved AF patients’ knowledge, medication adherence, quality of life, and satisfaction to anticoagulation compared with usual care.

#### Limitations Should Be Recognized

- Demands of self-management may be excessive for even well-intentioned patients required to be facile with setting up their own medical monitoring device, assessing frequency of download, interpreting and acting on data when required, and troubleshooting. These are not trivial challenges.

### 5.2. Behavioral Modification

#### Individual Health Status Has Been Found to Be a Strong Independent Predictor of Mortality and Cardiovascular Events

mHealth may catalyze positive behavioral change and facilitate health care (Rumsfeld 2013).^16^ An induced healthy user effect was likely the basis of survival benefit among CIED patients adhering more closely to remote management (Varma 2015).^17^ mHealth may support patients with text messaging (Chow 2015)^18^ or mobile applications to remind patients of medication doses and times, as well as medical appointments (but synchronization with health care providers and EMR is generally lacking). The Just-in-Time adaptive intervention premise is to provide the appropriate type and amount of support to an individual at the correct time, with the ability to adjust depending on the person’s current internal and situational factors (Nahum-Shani 2018).^19^ mHealth technology is an ideal platform to facilitate Just-in-Time adaptive interventions by providing real-time personalized information, which can be utilized to inform the intervention delivered. Just-in-Time adaptive interventions have been widely used for health promotion and to support behavior change, but evidence of their efficacy is limited (Gustafson 2014, Patrick 2009, Riley 2008).^20–22^ Timing is integral to the perception of benefit, as is receptivity to accept and use the support (Nahum-Shani 2015).^23^ Bespoke, multifaceted mHealth tools with motivational messages and incorporating gamification are most engaging (Coorey 2018, Gandhi 2017, Park 2016, Pfaeffli 2016).^8–11^

Incorporation of gamification strategies (eg, rewards, prizes, avatars, performance feedback, leaderboards, competitions, and social connection) into mHealth promotes patient engagement and sustains healthy behaviors (Blondon 2018, Cugelman 2013, Edwards 2016, Johnson 2016, Sardi 2017).^24–28^ However, a recent systematic review demonstrated that only 4% (64 of 1680) of English language top-rated health apps incorporated ≥1 gaming feature (Edwards 2016).^26^ There is limited hypothesis-generated data for these mHealth interventions, and their efficacy in this context is as yet unmeasured. Self-regulatory behavior change techniques, such as feedback and monitoring (including self-monitoring), comparison of behavior, rewards, incentives, and threats, and social support, are the most common behavior change techniques used in gamification apps and are frequently utilized in successful nongaming apps targeting health promotion and secondary prevention (Conroy 2014, Direito 2014, Edwards 2016).^26,29,30^ Engaging with apps involving gamification can also improve emotional well-being through feelings of accomplishment and social connectivity (Johnson 2016).^27^

### 5.3. Patients as Part of a Community

Incorporation of a patient as part of a wider community may offer benefits. Social networking is widely used for health (Fox 2011).^31^ Online communities enable individuals to meet, share their experiences, discuss treatment, and receive and provide support from peers, patient organizations, or HCPs (Fox 2011, Swan 2009, Swan 2012).^31–33^ While crowdsourcing via the internet and social networks allows collective sharing and exchange of information from a large number of people, the integrity and accuracy of such information remains largely unvetted and as such may be unreliable (Besaleva 2014).^34^

### 5.4. Maintaining Patient Engagement

Sustaining healthy behaviors and minimizing intervention fatigue is paramount to long-term maintenance. Although mHealth may help to maintain motivation, available data demonstrate significant attrition with mHealth interventions targeting risk factors and chronic conditions, even when people report liking the intervention and have purchased it (Chaudhry 2010, Flores Mateo 2015, Fukuoka 2015, Morgan 2017, Owen 2015, Simblett 2018, Whitehead 2016, Endeavour Partners 2017, Perez 2019).^35–43^

A representative patient’s experience is described below:

“A few years ago (2017) a friend told me about a new app that he had installed on his iPhone that would allow him to measure his heart rate through a fingertip pulse. Having an irregular heartbeat, under control through medication, I was very interested to try the new app. I thought it would provide me the opportunity to know more about myself, specifically how my heart operated under stress and at different times of day, before, during and after physical exertion of a variety of my favorite sports and pastimes like tennis, golf, biking and fly fishing.

At first, I was quite satisfied with the rudimentary calculations. Then I noticed during my international business travels that the device was often down during US nighttime hours during which time I thought the ‘hosts’ were making repairs or improvements. I also noticed that there were several radically incorrect readings especially during early morning hours. It simply wasn’t performing up to the standards of more traditional monitoring devices. I found as well that the host’s increasing attempt to up-sell to premium packages and other online health management tools became quite burdensome.

Before long, I felt almost addicted to the device and ultimately quit on it altogether. In retrospect, I believe that if I had had a proper introduction to the device by a trained medical specialist, I might have had a different expectation of this online tool, how to use it and how to interpret its data output.”

Understanding the basis for health-protective behavior is vital (Dunton 2018).^44^ Many apps, including those from the national heart foundations (websites),^45–47^ are available to support healthy lifestyle choices, but their efficacy remains largely untested or is limited by design features (ie, small sample sizes and selection bias). Cost, service connectivity, and credibility of information sources are important factors. However, patient engagement may be jeopardized by worries about privacy and personal data security (Burke 2015, Chow 2016, Kumar 2013, Steinhubl 2015).^5,48–50^

#### Continued Clinic Support

The level and duration of clinic support needed will likely depend on the condition monitored and goals for treatment. Reduction in compulsory routine in-clinic evaluations and reliance on continuous RM improved retention to long-term follow-up of patients with CIEDs (Varma 2014).^51^ In 1 HF trial, gain was related to the period of remote instruction. Whether this indicates that efficacy of the active program had peaked and stabilized or that it needed to be sustained is unclear (Varma 2020).^52^ Ideally, a training program should be finite in time but its effects durable.

### 5.5. Digital Divide

Although mHealth is highly promising in transforming health care, it can potentially exacerbate disparities in health care along sociodemographic lines.

Older people are perceived to engage less with mHealth. A 2017 Pew Research Center survey found that 92% of 18 to 29 year olds and 74% of 50 to 64 year olds own a smartphone (Pew 2017).^53^ However, the lack of familiarity with the technology and access to mobile devices, rather than lack of engagement per se, remain the principal barriers (Coorey 2018, Gallagher 2017, Tarakji 2018).^8,54,55^ Older users of mHealth prefer personalized information, which is clearly presented and is easy to navigate (Neubeck 2015).^56^

There is also disparity across the educational spectrum, with smartphone usage in 57% of the population with less than high school education and 91% of the population who graduated from college.

Smartphone use differs by income, with smartphone usage in 67% of the population with annual income ≤$30 000 and 93% of the population with income ≥$75 000 (Pew Research Center 2018).^57^ Limited evidence from the United States suggests that, although there is some variation in the mHealth use related to ethnicity, Black and Hispanic Americans are not disadvantaged (Martin 2012).^58^ mHealth permits information and apps to be tailored appropriately for language, literacy levels (including text-to-speech technology), and cultural differences to promote engagement (Coorey 2018, Neubeck 2017, Redfern 2016).^8,59,60^

There is heterogeneity of mHealth availability among different countries (Varma 2020).^52^ Even some of the best studied and FDA- and CE-approved technologies described here may be currently unavailable due to regulatory or marketing rules or simply unaffordable to either individuals or health care systems in many other countries.

As health care systems leverage and incorporate smartphone-based technology in their workflow and processes, a strategy is needed in parallel to ensure that those who do not have access to smartphone-based technology will continue to receive appropriate high-quality care. This critical initiative will require consensus and action among all stakeholders including HCPs, hospital systems, insurance providers, and state and federal government agencies. Thus enabled, mHealth promises improved patient outcomes in resource-limited areas (Bhavnani 2017).^61^

## 

References: Section 51.HendriksJMde WitRCrijnsHJVrijhoefHJPrinsMHPistersRPisonLABlaauwYTielemanRG
Nurse-led care vs. usual care for patients with atrial fibrillation: results of a randomized trial of integrated chronic care vs. routine clinical care in ambulatory patients with atrial fibrillation.
Eur Heart J. 2012;33:2692–2699. doi: 10.1093/eurheartj/ehs0712245365410.1093/eurheartj/ehs0712.LinJSO’ConnorEAEvansCVSengerCARowlandMGGroomHC
Behavioral counseling to promote a healthy lifestyle for cardiovascular disease prevention in persons with cardiovascular risk factors: an updated systematic evidence review for the U.S. Preventive Services Task Force [Internet].
2014
Agency for Healthcare Research and Quality (US)
Report No.: 13-05179-EF-1. Accessed January 26, 2021. https://www.ncbi.nlm.nih.gov/books/NBK241537/252326333.AngaranPMarianoZDraganVZouLAtzemaCLMangatIDorianP
The atrial fibrillation therapies after ER visit: outpatient care for patients with acute AF - the AFTER3 study.
J Atr Fibrillation. 2015;7:1187
doi: 10.4022/jafib.11872795715010.4022/jafib.1187PMC51352184.U.S. Department of Veterans Affairs. VA to provide capability for veterans to access their VA health data on Apple iPhones.
Published on February 11, 2019. Accessed January 26, 2021. https://www.va.gov/opa/pressrel/pressrelease.cfm?id=51995.ChowCKAriyarathnaNIslamSMThiagalingamARedfernJ
mHealth in cardiovascular health care.
Heart Lung Circ. 2016;25:802–807. doi: 10.1016/j.hlc.2016.04.0092726238910.1016/j.hlc.2016.04.0096.WalshJAIIITopolEJSteinhublSR
Novel wireless devices for cardiac monitoring.
Circulation. 2014;130:573–581.2511418610.1161/CIRCULATIONAHA.114.009024PMC41353737.SlotwinerDJTarakjiKGAl-KhatibSMPassmanRSSaxonLAPetersNSMcCallDTurakhiaMPSchaefferJMendenhallGS
Transparent sharing of digital health data: a call to action.
Heart Rhythm. 2019;16:e95–e106. doi: 10.1016/j.hrthm.2019.04.0423107780210.1016/j.hrthm.2019.04.0428.CooreyGMNeubeckLMulleyJRedfernJ
Effectiveness, acceptability and usefulness of mobile applications for cardiovascular disease self-management: systematic review with meta-synthesis of quantitative and qualitative data.
Eur J Prev Cardiol. 2018;25:505–521. doi: 10.1177/20474873177509132931336310.1177/20474873177509139.GandhiSChenSHongLSunKGongELiCYanLLSchwalmJD
Effect of mobile health interventions on the secondary prevention of cardiovascular disease: systematic review and meta-analysis.
Can J Cardiol. 2017;33:219–231. doi: 10.1016/j.cjca.2016.08.0172795604310.1016/j.cjca.2016.08.01710.ParkLGBeattyAStaffordZWhooleyMA
Mobile phone interventions for the secondary prevention of cardiovascular disease.
Prog Cardiovasc Dis. 2016;58:639–650. doi: 10.1016/j.pcad.2016.03.0022700124510.1016/j.pcad.2016.03.002PMC490482711.Pfaeffli DaleLDobsonRWhittakerRMaddisonR
The effectiveness of mobile-health behaviour change interventions for cardiovascular disease self-management: a systematic review.
Eur J Prev Cardiol. 2016;23:801–817. doi: 10.1177/20474873156134622649009310.1177/204748731561346212.HägglundELyngåPFrieFUllmanBPerssonHMelinMHagermanI
Patient-centred home-based management of heart failure. Findings from a randomised clinical trial evaluating a tablet computer for self-care, quality of life and effects on knowledge.
Scand Cardiovasc J. 2015;49:193–199. doi: 10.3109/14017431.2015.10353192596896810.3109/14017431.2015.103531913.VarnfieldMKarunanithiMLeeCKHoneymanEArnoldDDingHSmithCWaltersDL
Smartphone-based home care model improved use of cardiac rehabilitation in postmyocardial infarction patients: results from a randomised controlled trial.
Heart. 2014;100:1770–1779. doi: 10.1136/heartjnl-2014-3057832497308310.1136/heartjnl-2014-30578314.KotechaDChuaWWLFabritzLHendriksJCasadeiBSchottenUVardasPHeidbuchelHDeanVKirchhofP; European Society of Cardiology (ESC) Atrial Fibrillation Guidelines Taskforce, the CATCH ME Consortium and the European Heart Rhythm Association (EHRA). European Society of Cardiology smartphone and tablet applications for patients with atrial fibrillation and their health care providers.
Europace. 2018;20:225–233. doi: 10.1093/europace/eux2992904054810.1093/europace/eux299PMC583409715.GuoYChenYLaneDALiuLWangYLipGYH
Mobile health technology for atrial fibrillation management integrating decision support, education, and patient involvement: mAF app trial.
Am J Med. 2017;130:1388–1396.e6. doi: 10.1016/j.amjmed.2017.07.0032884754610.1016/j.amjmed.2017.07.00316.RumsfeldJSAlexanderKPGoffDCJrGrahamMMHoPMMasoudiFAMoserDKRogerVLSlaughterMSSmolderenKG; American Heart Association Council on Quality of Care and Outcomes Research, Council on Cardiovascular and Stroke Nursing, Council on Epidemiology and Prevention, Council on Peripheral Vascular Disease, and Stroke Council. Cardiovascular health: the importance of measuring patient-reported health status: a scientific statement from the American Heart Association.
Circulation. 2013;127:2233–2249. doi: 10.1161/CIR.0b013e3182949a2e2364877810.1161/CIR.0b013e3182949a2e17.VarmaNPicciniJPSnellJFischerADalalNMittalS
The relationship between level of adherence to automatic wireless remote monitoring and survival in pacemaker and defibrillator patients.
J Am Coll Cardiol. 2015;65:2601–2610. doi: 10.1016/j.jacc.2015.04.0332598300810.1016/j.jacc.2015.04.03318.ChowCKRedfernJHillisGSThakkarJSantoKHackettMLJanSGravesNde KeizerLBarryT
Effect of lifestyle-focused text messaging on risk factor modification in patients with coronary heart disease: a randomized clinical trial.
JAMA. 2015;314:1255–1263. doi: 10.1001/jama.2015.109452639384810.1001/jama.2015.1094519.Nahum-ShaniISmithSNSpringBJCollinsLMWitkiewitzKTewariAMurphySA
Just-in-time adaptive interventions (JITAIs) in mobile health: key components and design principles for ongoing health behavior support.
Ann Behav Med. 2018;52:446–462. doi: 10.1007/s12160-016-9830-82766357810.1007/s12160-016-9830-8PMC536407620.GustafsonDHMcTavishFMChihMYAtwoodAKJohnsonRABoyleMGLevyMSDriscollHChisholmSMDillenburgL
A smartphone application to support recovery from alcoholism: a randomized clinical trial.
JAMA Psychiatry. 2014;71:566–572. doi: 10.1001/jamapsychiatry.2013.46422467116510.1001/jamapsychiatry.2013.4642PMC401616721.PatrickKRaabFAdamsMADillonLZabinskiMRockCLGriswoldWGNormanGJ
A text message-based intervention for weight loss: randomized controlled trial.
J Med Internet Res. 2009;11:e1
doi: 10.2196/jmir.11001914143310.2196/jmir.1100PMC272907322.RileyWObermayerJJean-MaryJ
Internet and mobile phone text messaging intervention for college smokers.
J Am Coll Health. 2008;57:245–248. doi: 10.3200/JACH.57.2.245-2481880954210.3200/JACH.57.2.245-24823.Nahum-ShaniIHeklerEBSpruijt-MetzD
Building health behavior models to guide the development of just-in-time adaptive interventions: a pragmatic framework.
Health Psychol. 2015;34S:1209–1219. doi: 10.1037/hea00003062665146210.1037/hea0000306PMC473226824.BlondonKMeyerPLovisCEhrlerF
Gamification and mHealth: a model to bolster cardiovascular disease self-management.
Swiss Medical Informatics
Accessed January 26, 2021. https://medical-informatics.ch/article/doi/smi.33.00398. doi: 10.4414/smi.33.0039825.CugelmanB
Gamification: what it is and why it matters to digital health behavior change developers.
JMIR Serious Games. 2013;1:e3
doi: 10.2196/games.31392565875410.2196/games.3139PMC430781726.EdwardsEALumsdenJRivasCSteedLEdwardsLAThiyagarajanASohanpalRCatonHGriffithsCJMunafòMR
Gamification for health promotion: systematic review of behaviour change techniques in smartphone apps.
BMJ Open. 2016;6:e012447
doi: 10.1136/bmjopen-2016-01244710.1136/bmjopen-2016-012447PMC50736292770782927.JohnsonDDeterdingSKuhnKAStanevaAStoyanovSHidesL
Gamification for health and wellbeing: a systematic review of the literature.
Internet Interv. 2016;6:89–106. doi: 10.1016/j.invent.2016.10.0023013581810.1016/j.invent.2016.10.002PMC609629728.SardiLIdriAFernández-AlemánJL
A systematic review of gamification in e-Health.
J Biomed Inform. 2017;71:31–48. doi: 10.1016/j.jbi.2017.05.0112853606210.1016/j.jbi.2017.05.01129.ConroyDEYangCHMaherJP
Behavior change techniques in top-ranked mobile apps for physical activity.
Am J Prev Med. 2014;46:649–652. doi: 10.1016/j.amepre.2014.01.0102484274210.1016/j.amepre.2014.01.01030.DireitoADaleLPShieldsEDobsonRWhittakerRMaddisonR
Do physical activity and dietary smartphone applications incorporate evidence-based behaviour change techniques?
BMC Public Health. 2014;14:646
doi: 10.1186/1471-2458-14-6462496580510.1186/1471-2458-14-646PMC408069331.FoxS
The Social Life of Health Information.
Accessed January 26, 2021. https://www.pewresearch.org/fact-tank/2014/01/15/the-social-life-of-health-information/32.SwanM
Emerging patient-driven health care models: an examination of health social networks, consumer personalized medicine and quantified self-tracking.
Int J Environ Res Public Health. 2009;6:492–525. doi: 10.3390/ijerph60204921944039610.3390/ijerph6020492PMC267235833.SwanM
Health 2050: the realization of personalized medicine through crowdsourcing, the quantified self, and the participatory biocitizen.
J Pers Med. 2012;2:93–118.2556220310.3390/jpm2030093PMC425136734.BesalevaLIWeaverAC
CrowdHelp: m-Health Application for Emergency Response Improvement through Crowdsourced and Sensor-Detected Information.
2014
Accessed January 26, 2021. https://ieeexplore.ieee.org/document/669333535.ChaudhrySIMatteraJACurtisJPSpertusJAHerrinJLinZPhillipsCOHodshonBVCooperLSKrumholzHM
Telemonitoring in patients with heart failure.
N Engl J Med. 2010;363:2301–2309. doi: 10.1056/NEJMoa10100292108083510.1056/NEJMoa1010029PMC323739436.Flores MateoGGranado-FontEFerré-GrauCMontaña-CarrerasX
Mobile phone apps to promote weight loss and increase physical activity: a systematic review and meta-analysis.
J Med Internet Res. 2015;17:e253
doi: 10.2196/jmir.48362655431410.2196/jmir.4836PMC470496537.FukuokaYGayCHaskellWAraiSVittinghoffE
Identifying factors associated with dropout during prerandomization run-in period from an mHealth physical activity education study: the mPED trial.
JMIR Mhealth Uhealth. 2015;3:e34
doi: 10.2196/mhealth.39282587275410.2196/mhealth.3928PMC441136338.MorganJMKittSGillJMcCombJMNgGARafteryJRoderickPSeedAWilliamsSGWitteKK
Remote management of heart failure using implantable electronic devices.
Eur Heart J. 2017;38:2352–2360. doi: 10.1093/eurheartj/ehx2272857523510.1093/eurheartj/ehx227PMC583754839.OwenJEJaworskiBKKuhnEMakin-ByrdKNRamseyKMHoffmanJE
mHealth in the wild: using novel data to examine the reach, use, and impact of PTSD coach.
JMIR Ment Health. 2015;2:e7
doi: 10.2196/mental.39352654391310.2196/mental.3935PMC460737440.SimblettSGreerBMatchamFCurtisHPolhemusAFerrãoJGamblePWykesT
Barriers to and facilitators of engagement with remote measurement technology for managing health: systematic review and content analysis of findings.
J Med Internet Res. 2018;20:e10480
doi: 10.2196/104803000199710.2196/10480PMC606269241.WhiteheadLSeatonP
The effectiveness of self-management mobile phone and tablet apps in long-term condition management: a systematic review.
J Med Internet Res. 2016;18:e97
doi: 10.2196/jmir.48832718529510.2196/jmir.4883PMC488609942.Endeavour Partners. Inside wearables: how the science of human behavior change offers the secret to long-term.
2017
Accessed January 26, 2021. https://medium.com/@endeavourprtnrs/inside-wearable-how-the-science-of-human-behavior-change-offers-the-secret-to-long-term-engagement-a15b3c7d4cf343.PerezMVMahaffeyKWHedlinHRumsfeldJSGarciaAFerrisTBalasubramanianVRussoAMRajmaneACheungL; Apple Heart Study Investigators. Large-scale assessment of a smartwatch to identify atrial fibrillation.
N Engl J Med. 2019;381:1909–1917. doi: 10.1056/NEJMoa19011833172215110.1056/NEJMoa1901183PMC811260544.DuntonGF
Sustaining health-protective behaviors such as physical activity and healthy eating.
JAMA. 2018;320:639–640. doi: 10.1001/jama.2018.66212985204610.1001/jama.2018.6621PMC752454345.National Heart Foundation of Australia. My Heart, My Life.
2014
Accessed October 8, 2014. https://myheartmylife.org.au/46.American Heart Association. Sustaining healthy behaviours (AHA Simple 7).
Last updated May 2018. Accessed February 14, 2019. https://www.heart.org/en/healthy-living/healthy-lifestyle/my-life-check--lifes-simple-747.British Heart Foundation. Our healthy recipe finder app.
Accessed October 8, 2014. Accessed January 26, 2021. http://www.bhf.org.uk/heart-health/prevention/healthy-eating/our-healthy-recipe-finder-app.aspx48.BurkeLEMaJAzarKMBennettGGPetersonEDZhengYRileyWStephensJShahSHSuffolettoB; American Heart Association Publications Committee of the Council on Epidemiology and Prevention, Behavior Change Committee of the Council on Cardiometabolic Health, Council on Cardiovascular and Stroke Nursing, Council on Functional Genomics and Translational Biology, Council on Quality of Care and Outcomes Research, and Stroke Council. Current science on consumer use of mobile health for cardiovascular disease prevention: a scientific statement From the American Heart Association.
Circulation. 2015;132:1157–1213. doi: 10.1161/CIR.00000000000002322627189210.1161/CIR.0000000000000232PMC731338049.KumarSNilsenWJAbernethyAAtienzaAPatrickKPavelMRileyWTSharASpringBSpruijt-MetzD
Mobile health technology evaluation: the mHealth evidence workshop.
Am J Prev Med. 2013;45:228–236. doi: 10.1016/j.amepre.2013.03.0172386703110.1016/j.amepre.2013.03.017PMC380314650.SteinhublSRMuseEDTopolEJ
The emerging field of mobile health.
Sci Transl Med. 2015;7:283rv3
doi: 10.1126/scitranslmed.aaa348710.1126/scitranslmed.aaa3487PMC47488382587789451.VarmaNMichalskiJStamblerBPavriBB; TRUST Investigators. Superiority of automatic remote monitoring compared with in-person evaluation for scheduled ICD follow-up in the TRUST trial - testing execution of the recommendations.
Eur Heart J. 2014;35:1345–1352. doi: 10.1093/eurheartj/ehu0662459586410.1093/eurheartj/ehu066PMC402861052.VarmaN
Remote management of patients with heart failure-how long should it go on?
Lancet Digit Health. 2020;2:e2–e3. doi: 10.1016/S2589-7500(19)30221-33332803610.1016/S2589-7500(19)30221-353.SmithA
Record shares of Americans now own smartphones, have home broadband.
2017
Pew Research Center
Accessed January 26, 2021. https://www.pewresearch.org/fact-tank/2017/01/12/evolution-of-technology/54.GallagherRRoachKSadlerLGlinatsisHBelshawJKirknessAZhangLGallagherPPaullGGaoY
Mobile technology use across age groups in patients eligible for cardiac rehabilitation: survey study.
JMIR Mhealth Uhealth. 2017;5:e161
doi: 10.2196/mhealth.83522906642510.2196/mhealth.8352PMC567602755.TarakjiKGVivesCAPatelASFaganDHSimsJJVarmaN
Success of pacemaker remote monitoring using app-based technology: does patient age matter?
Pacing Clin Electrophysiol. 2018;41:1329–1335. doi: 10.1111/pace.134613005501310.1111/pace.1346156.NeubeckLLowresNBenjaminEJFreedmanSBCooreyGRedfernJ
The mobile revolution–using smartphone apps to prevent cardiovascular disease.
Nat Rev Cardiol. 2015;12:350–360. doi: 10.1038/nrcardio.2015.342580171410.1038/nrcardio.2015.3457.Pew Research Center. Mobile Fact Sheet.
2018
Accessed January 26, 2021. https://www.pewresearch.org/internet/fact-sheet/mobile/58.MartinT
Assessing mHealth: opportunities and barriers to patient engagement.
J Health Care Poor Underserved. 2012;23:935–941. doi: 10.1353/hpu.2012.00872421214410.1353/hpu.2012.008759.NeubeckLCartledgeSDawkesSGallagherR
Is there an app for that? Mobile phones and secondary prevention of cardiovascular disease.
Curr Opin Cardiol. 2017;32:567–571. doi: 10.1097/HCO.00000000000004282861410410.1097/HCO.000000000000042860.RedfernJSantoKCooreyGThakkarJHackettMThiagalingamAChowCK
Factors influencing engagement, perceived usefulness and behavioral mechanisms associated with a text message support program.
PLoS One. 2016;11:e0163929
doi: 10.1371/journal.pone.01639292774124410.1371/journal.pone.0163929PMC506514761.BhavnaniSPSolaSAdamsDVenkateshvaranADashPKSenguptaPP; ASEF-VALUES Investigators. A randomized trial of pocket-echocardiography integrated mobile health device assessments in modern structural heart disease clinics.
JACC Cardiovasc Imaging. 2018;11:546–557. doi: 10.1016/j.jcmg.2017.06.0192891768810.1016/j.jcmg.2017.06.019

## 6. Clinical Trials

mHealth may have particular impact on trials of heart rhythm disorders. Traditionally, clinical trials testing drugs and devices for arrhythmias utilized time-to-event outcomes and analyses, such as first recurrence of AF after a blanking period (Piccini 2017).^1^ Patients randomized to the control and intervention would be monitored intermittently, either with ambulatory devices or in-clinic visit. Such monitoring had limited sensitivity for recurrent arrhythmias, including symptomatic and asymptomatic episodes. Furthermore, time-to-first event may not accurately capture reductions in arrhythmia burden, which have also been shown to be beneficial in recent randomized trials (Andrade 2019).^2^ While CIEDs such as pacemakers and defibrillators can be leveraged for continuous monitoring (Varma 2005),^3^ these studies do not generalize to broader CIED-free populations. Implantable loop recorders may have a potential role but are costly and, unless used for clinical indications, difficult to justify simply for study event ascertainment.

There are a variety of free-standing handheld ECG monitors, some of which have automated AF detection (Table 1). However, many do not have cellular or networking capability and, therefore, generally cannot transmit data or findings in real time. This is where smart- or mobile-connected arrhythmia and pulse detection technologies have significant promise. These may enhance detection and measurement of clinical outcomes while also allowing for remote or virtual data collection without the need for site-based study visits. Examples include remote rhythm assessment with single or multilead ECGs from smartphone- or smartwatch-based technologies and automatic ascertainment of hospitalizations using smartphone-based geofencing (Nguyen 2017).^4^ These operational enhancements, in turn, can improve participant satisfaction, reduce cost, improve study efficiency, and facilitate or expand enrollment. An example is the ongoing Health eHeart study—a site-free cardiovascular research study that leverages self-reported data, data from wearable sensors, EHRs, and other importable big data to enable rapid-cycle, low-cost interventional and observational cardiovascular research (https://www.health-eheartstudy.org/).^5^

### Screening

Two recent large-scale studies highlight the potential advantages of mHealth for AF screening and treatment.

#### The Apple Heart Study

This was a highly pragmatic, single-arm, investigational device exemption study designed to test the performance and safety of a photoplethysmography-based irregular rhythm–detection algorithm on the Apple Watch for identification of AF (Perez 2019, Turakhia 2019).^6,7^ The study was a siteless bring-your-own-device study, such that participants needed their own compatible smartphone and watch to enroll online. All study procedures, including eligibility verification, onboarding, enrollment, and data collection, were performed via the study app, which could be downloaded from the app store. If a participant received an irregular pulse notification, then subsequent study visits were done via video conferencing to study physicians directly with the app. The study enrolled over 419 000 participants without preexisting AF in just an 8-month period, in large part due to the pragmatic, virtual design and easy accessibility (Figure 4). The algorithm was found to have a positive predictive value of simultaneous ECG-confirmed AF of 0.84 (Perez 2019).^6^ Only 0.5% of the enrolled population received any irregular pulse notification, but 3.2% of those aged ≥65 years received notifications. However, only 153 of 450 (34%) patients had AF detected by subsequent single ECG patches after the irregular rhythm notification was received. This may reflect the paroxysmal nature of early-stage AF rather than explicit false positives. Because the study only administered ECG patch morning to those with irregular rhythm notification rather than the entire cohort or to negative controls, the negative predictive value was not estimated. It should be noticed that the Apple Heart Study was in a population without diagnosed AF; test performance and diagnostic yield could be considerably different in a population with known AF, and this software is not approved for use for AF surveillance in established AF.

#### The Huawei Heart Study

A similar study was performed using smart device-based (Huawei fitness band or smartwatch) photoplethysmography technology (Guo 2019).^7^ The algorithm had been validated with over 29 485 photoplethysmography signals before commencement of the trial. More than 246 000 people downloaded the photoplethysmography screening app, of whom about 187 000 individuals monitored their pulse rhythm for 7 months. AF was found in 0.23% (slightly lower than Apple Heart, possibly due to a younger and healthier enrolled cohort). Validation was achieved in 87% (positive predictive value >90%) compared with 34% in Apple Heart. The results indicated that this was a feasible frequent continuous monitoring approach for the screening and early detection of AF in a large population.

A significant observation was that CDS tools provided enabled management decisions, for example, almost 80% high-risk patients were anticoagulated. Subsequent enrollment into the mAFA II trial showed significantly reduced risk of rehospitalization and clinical adverse events (Guo 2020).^8^ These trial results encourage incorporation of such technology effectively into the AF management pathways at multiple levels, that is, screening and detection of AF, as well as early interventions to reduce stroke and other AF-related complications.

#### The Fitbit Study

Another large-scale virtual study to identify episodes of irregular heart rhythm suggestive of AF was announced by Fitbit in May 2020 (HRS 2020, held on May 7, 2020).^9^

### Point of Care

The next step beyond parameterizing safety could be to actionably guide therapy at the point of care (Figure 6).^10^ For example, patients could obtain ECGs before and after taking pill-in-the-pocket antiarrhythmic drug therapy such as flecainide to confirm AF, ensure no QRS widening, and confirm restoration of sinus rhythm. A similar approach has been proposed for rhythm-guided use of direct OACs in lower risk AF patients with infrequent episodes either spontaneously or as the result of a rhythm control intervention including drugs and ablation; a randomized trial is in development (Passman 2016).^11^ The use of smartwatch-guided rate control as a treatment strategy could also be tested, as this may provide a more personalized approach rather than prior randomized trials of lenient versus strict rate control that used population level rather than personalized HR treatment thresholds (Van Gelder 2010).^12^

### Questions

#### Generalizability

This is key to application of results from trials. mHealth is widely available and often simple to apply and wear.

- Older individuals and those with low health literacy may find technologies difficult to use (Section 5.5), and this may be compounded by disease state, for example, previous stroke.
- Cost and service plans associated with smartphones and smartwatches may preclude their use in lower socioeconomic populations who are already underrepresented in clinical trials and in many geographies.

Thus patients who volunteer in mHealth studies in the United States are more likely to be White/non-Hispanic, more educated, and less likely to have disease.

#### Adherence

mHealth-based evaluation of clinical end points may be confounded if adherence is low, particularly if there are no secondary means of end point assessments (Guo 2017).^13^ Virtual designs may be more susceptible to the loss of participant engagement. For example, if monitoring is completely reliant upon mHealth technology and there are no traditional measures or in-person visits to assess arrhythmia, then significant missing data due to low adherence may become a major limitation that could imperil the validity and generalizability of the findings. For example, among 2161 of the 419 297 patients who received an irregular pulse notification in the Apple Heart Study, only 945 completed a subsequent protocoled first study visit. Of these 658 ambulatory ECG patches shipped, there were only 450 with returned and analyzable data (Perez 2019).^6^

Development of effective strategies to increase retention and maintain high engagement remains an unmet need and is an area ripe for more research.

#### Outcomes

These are key to adoption and reimbursement. More specifically, the clinical and prognostic impact of new outcome measures based on mHealth technologies may not be clear.

This is important for AF. For example, how do changes in AF burden compare to reductions in time to symptomatic sustained AF? Should AF identified on near-continuous smartwatch monitoring be considered equivalent to AF diagnosed at hospitalization or in clinic? There is a growing body of literature that the dose of AF burden matters for a variety of important clinical end points, including stroke, HF, and death (Section 3.1.3; Chen 2018, Glotzer 2009, Kaplan 2019, Piccini 2019, Wong 2018).^14–18^ Does pill-in-the-pocket direct oral anticoagulant treatment of paroxysmal atrial fibrillation adequately cover the risk of stroke? Some measures remain less well studied, like the occurrence of irregularity with a wearable pulse-based monitor system, particularly without ECG confirmation.

Since these mHealth prediagnostic or diagnostic tools may then be directly tied to initiation or termination of treatment, rigorous evaluation of clinical safety and efficacy will be required and in some cases, warrant a combined drug-device regulatory approval.

Despite these challenges, there is enormous potential for patients to use these technologies to self-monitor their arrhythmia treatment and extend this to manage comorbidities (Section 4). The process of data transparency and accessibility to the patient may improve the patient’s engagement with their overall care, even if the data are not directly actionable by the patient. The restriction to clinic access during the SARS-CoV-2 pandemic has accelerated the adoption of mHealth solutions (Varma 2020).^10^ ECGs for clinical trials were recorded by smart devices and assessed at virtual visits instead of routine in-person evaluations. In some cases, the entire management of clinical trials went online.

## 

References: Section 61.PicciniJPClarkRLKoweyPRMittalSDunnmonPStockbridgeNReiffelJATurakhiaMPZieglerPDKleimanRB
Long-term electrocardiographic safety monitoring in clinical drug development: a report from the Cardiac Safety Research Consortium.
Am Heart J. 2017;187:156–169. doi: 10.1016/j.ahj.2017.01.0122845479910.1016/j.ahj.2017.01.0122.AndradeJGChampagneJDubucMDeyellMWVermaAMacleLLeong-SitPNovakPBadra-VerduMSappJ; CIRCA-DOSE Study Investigators. Cryoballoon or radiofrequency ablation for atrial fibrillation assessed by continuous monitoring: a randomized clinical trial.
Circulation. 2019;140:1779–1788. doi: 10.1161/CIRCULATIONAHA.119.0426223163053810.1161/CIRCULATIONAHA.119.0426223.VarmaNStamblerBChunS
Detection of atrial fibrillation by implanted devices with wireless data transmission capability.
Pacing Clin Electrophysiol. 2005;28suppl 1S133–S136. doi: 10.1111/j.1540-8159.2005.00083.x1568348010.1111/j.1540-8159.2005.00083.x4.NguyenKTOlginJEPletcherMJNgMKayeLMoturuSGladstoneRAMalladiCFannAFMaguireC
Smartphone-based geofencing to ascertain hospitalizations.
Circ Cardiovasc Qual Outcomes. 2017;10:e003326.2832575110.1161/CIRCOUTCOMES.116.003326PMC53632805.https://www.health-eheartstudy.org/. Accessed January 26, 20216.PerezMVMahaffeyKWHedlinHRumsfeldJSGarciaAFerrisTBalasubramanianVRussoAMRajmaneACheungL; Apple Heart Study Investigators. Large-scale assessment of a smartwatch to identify atrial fibrillation.
N Engl J Med. 2019;381:1909–1917. doi: 10.1056/NEJMoa19011833172215110.1056/NEJMoa1901183PMC81126057.TurakhiaMPDesaiMHedlinHRajmaneATalatiNFerrisTDesaiSNagDPatelMKoweyP
Rationale and design of a large-scale, app-based study to identify cardiac arrhythmias using a smartwatch: the Apple Heart Study.
Am Heart J. 2019;207:66–75. doi: 10.1016/j.ahj.2018.09.0023039258410.1016/j.ahj.2018.09.002PMC80990487.GuoYWangHZhangHLiuTLiangZXiaYYanLXingYShiHLiS; MAFA II Investigators. Mobile photoplethysmographic technology to detect atrial fibrillation.
J Am Coll Cardiol. 2019;74:2365–2375. doi: 10.1016/j.jacc.2019.08.0193148754510.1016/j.jacc.2019.08.0198.GuoYLaneDAWangLZhangHWangHZhangWWenJXingYWuFXiaY; mAF-App II Trial Investigators. Mobile health technology to improve care for patients with atrial fibrillation.
J Am Coll Cardiol. 2020;75:1523–1534. doi: 10.1016/j.jacc.2020.01.0523224136710.1016/j.jacc.2020.01.0529.MuoioD
Fitbit launches large-scale consumer health study to detect a-fib via heart rate sensors, algorithm.
HRS May 7, 2020. Accessed January 26, 2021. https://www.mobihealthnews.com/news/fitbit-launches-large-scale-consumer-health-study-detect-fib-heart-rate-sensors-algorithm10.VarmaNMarroucheNFAguinagaLAlbertCMArbeloEChoiJIChungMKConteGDagherLEpsteinLM
HRS/EHRA/APHRS/LAHRS/ACC/AHA worldwide practice update for telehealth and arrhythmia monitoring during and after a pandemic.
J Am Coll Cardiol. 2020;76:1363–1374. doi: 10.1016/j.jacc.2020.06.0193253493610.1016/j.jacc.2020.06.019PMC728908811.PassmanRLeong-SitPAndreiACHuskinATomsonTTBernsteinREllisEWaksJWZimetbaumP
Targeted anticoagulation for atrial fibrillation guided by continuous rhythm assessment with an insertable cardiac monitor: the rhythm evaluation for anticoagulation with continuous monitoring (REACT.COM) pilot study.
J Cardiovasc Electrophysiol. 2016;27:264–270. doi: 10.1111/jce.128642651122110.1111/jce.12864PMC478913512.Van GelderICGroenveldHFCrijnsHJTuiningaYSTijssenJGAlingsAMHillegeHLBergsma-KadijkJACornelJHKampO; RACE II Investigators. Lenient versus strict rate control in patients with atrial fibrillation.
N Engl J Med. 2010;362:1363–1373. doi: 10.1056/NEJMoa10013372023123210.1056/NEJMoa100133713.GuoXVittinghoffEOlginJEMarcusGMPletcherMJ
Volunteer participation in the health eHeart Study: a comparison with the US population.
Sci Rep. 2017;7:1956
doi: 10.1038/s41598-017-02232-y2851230310.1038/s41598-017-02232-yPMC543403914.ChenLYChungMKAllenLAEzekowitzMFurieKLMcCabePNoseworthyPAPerezMVTurakhiaMP; American Heart Association Council on Clinical Cardiology; Council on Cardiovascular and Stroke Nursing; Council on Quality of Care and Outcomes Research; and Stroke Council. Atrial fibrillation burden: moving beyond atrial fibrillation as a binary entity: a scientific statement from the American Heart Association.
Circulation. 2018;137:e623–e644. doi: 10.1161/CIR.00000000000005682966194410.1161/CIR.0000000000000568PMC846325815.GlotzerTVDaoudEGWyseDGSingerDEEzekowitzMDHilkerCMillerCQiDZieglerPD
The relationship between daily atrial tachyarrhythmia burden from implantable device diagnostics and stroke risk: the TRENDS study.
Circ Arrhythm Electrophysiol. 2009;2:474–480. doi: 10.1161/CIRCEP.109.8496381984391410.1161/CIRCEP.109.84963816.KaplanRMKoehlerJZieglerPDSarkarSZweibelSPassmanRS
Stroke risk as a function of atrial fibrillation duration and CHA2DS2-VASc score.
Circulation. 2019;140:1639–1646. doi: 10.1161/CIRCULATIONAHA.119.0413033156412610.1161/CIRCULATIONAHA.119.04130317.PicciniJPPassmanRTurakhiaMConnollyATNabutovskyYVarmaN
Atrial fibrillation burden, progression, and the risk of death: a case-crossover analysis in patients with cardiac implantable electronic devices.
Europace. 2019;21:404–413. doi: 10.1093/europace/euy2223046220810.1093/europace/euy22218.WongJAConenDVan GelderICMcIntyreWFCrijnsHJWangJGoldMRHohnloserSHLauCPCapucciA
Progression of device-detected subclinical atrial fibrillation and the risk of heart failure.
J Am Coll Cardiol. 2018;71:2603–2611. doi: 10.1016/j.jacc.2018.03.5192988011910.1016/j.jacc.2018.03.519

## 7. Operational Challenges

### 7.1. Health Care System—eHealth Monitoring and Hospital Ecosystem

#### Transmission

A fundamental but as yet unresolved challenge of incorporating mHealth into clinical practice is the channel of data communication between patient and provider. This may differ depending upon whether the data are physician facing (eg, for CIEDs) or patient facing (consumer digital health products, eg, the Apple Watch; Apple, Inc, Cupertino, CA).

##### CIEDs

Experience with CIEDs provides a framework. CIEDs generate voluminous quantities of eHealth data. In a single patient, this may be generated from distinct sources, that is, RM and in-person interrogations. Transmission from RM has been well worked out: data flow from the CIED to the remote transceiver and then to the manufacturer’s server for access by individual practices. Unfortunately, this is usually retrieved in an image format rendering the granular data uninterpretable by the practice’s EHR. When shared with the patient, the image file is posted on the EHR’s patient portal. These files are difficult for physicians to interpret and practically uninterpretable by the lay public. To engage patients and caregivers, the data will need to be provided in a format that enables the lay public to get a high-level summary of key features (such as battery status and remote monitor function status) with explanations and the ability to drill down to the more granular details for those individuals who wish to do so.

##### Consumer Digital Health Product Data

Consumers are rapidly adopting products to monitor their health status for early detection of abnormalities and for managing chronic diseases. These tools empower and engage patients in managing their health, but the basic task of sharing the data with their health care provider presents challenges. From a technical standpoint, many EHR portals do not permit patients to send attachments. Therefore, the patient and provider are left using email, which is not considered secure or HIPPA or GDPR (General Data Protection Regulation) compliant. Even if the EHR portal accepts attachments, incorporating the digital health data into the EHR remains ad hoc and inconsistent. The logistical and practical concerns frighten many care providers into discouraging their patients from using these devices. Concerns among providers include the fear of being inundated with unnecessary transmissions to review, as well as the concern that patients may send inappropriate data, for example, BP or glucose monitoring data, to their electrophysiologist. Cloud-based storage may avoid some of these challenges.

#### Interoperability—Lack of Organized Infrastructure to Receive Incoming the Data

Assimilating the data obtained from digital health tools, whether implantable or wearable, is proving to be one of the greatest clinical challenges. Clinicians feel increasingly burdened as both the volume of data and the sources of data increase. Creating the nomenclature and data models that would enable the information to be incorporated in the EMR is less a technical challenge but more a political challenge. It requires a consensus from the clinical community regarding definitions of the terminology and agreement on what data are required. For example, for pacemakers, there must be agreement on the definition of battery longevity, pacing thresholds, mode switch, etc. For CIEDs, this work has been done (https://www.iso.org/standard/63904.html; Slotwiner 2019).^1,2^ The next step is for EHR vendors to support the agreed-upon nomenclature and the data standard in which it is communicated. With these two building blocks, digital health data can be assimilated into the clinical workflow, enabling health care providers to review, manage, and document clinical impressions and recommendations within the environment of their EHR. This work is ongoing in the domain of CIEDs but has not started for wearable devices. It requires a coalition of clinicians, engineers, regulatory agencies, as well as regulatory and financial incentives for vendors. A high efficient computerized system with huge storage is necessary infrastructure and may provide the platform for predictive analytics.

#### Interoperability—Lack of Organized Infrastructure to Transmit Data and Instructions

There is interest in mHealth to support patients with text messaging (Chow 2015)^3^ or mobile applications to remind patients of medication doses and times or medical appointments. To be effective, this requires synchronization with health care providers, ideally by integration with the EMR, allowing changes in medications and doses, as well as appointments, to flow between patients and clinicians in an accurate and bidirectional manner (Spaulding 2019).^4^ However, EMR systems software is lacking such functionality and interoperability at this point (Ratwani 2018).^5^

### 7.2. Cybersecurity Guidance for mHealth Devices

Interconnection of medical devices and clinical data promises facilitation of clinical care but also creates opportunities for intrusions by maleficent actors (ie, hackers) to disable systems or access private health information (Jalali 2019, Kruse 2017).^6,7^ The motivation is largely financial. Health care facilities and medical device companies present attractive targets because a number of attack strategies can yield large financial rewards:

1. Ransomware: a hospital’s systems can be locked out (eg, data may be encrypted) until the attacker is paid (Mansfield-Devine 2016, *Network Security* 2016).^8,9^
2. Theft and sale of patient data (ie, private health information).
3. Company attack: a hacker may identify flaws in a system or device, short the company’s stock, and then make the flaws public. Alternatively, a maleficent user may try to harvest insider information from a breached company’s network. Attackers may compromise a company but not take any of the above actions. Instead, they may sell their methods or credentials to another group who will use them (Perkalis 2014).^10^

Scenarios where a cyber attack results in the deaths of individuals or groups (eg, by corrupting the firmware of a pacemaker or insulin pump) can be easily imagined and have been demonstrated by researchers (Klonoff 2015),^11^ but to date, no such attack is known to have occurred in the real world. It is possible that that this is because attacks against organizations are more profitable than attacks against individuals.

It is essential, therefore, to establish best practice methods to maintain patient safety and privacy in this new ecosystem of remotely managed devices and mass data collection.

#### 7.2.1. Hacking Strategies and Methods in mHealth Technologies

Often times, attackers will not directly compromise the system that they are after; they will instead start by compromising a weaker link. For example, if the goal is to obtain private health information about a specific patient, they may attempt to get the patient (or a staff member) to install a malicious app, compromising the rest of the phone, including email and other credentials. From this point, the attacker is in a better position to attack the actual target. The process of chaining exploits to work through a system is called pivoting. Each pivot or hop enables new privileges that bring the hacker closer to desired goals.

The easiest thing to exploit is often a person with phishing campaigns. A compromised email account can be used to reset passwords for other services and to distribute more realistic phishing messages. More technical attack pathways are used to compromise the RM components of a health care system, for example, wireless links (Bluetooth, Wi-Fi, etc), internet, and local network communications or servers (databases, web frontends, file servers, etc).

#### 7.2.2. Recommendations to the Manufacturer

It is not possible to create systems that canot be hacked. However, systems/devices should be designed to fail gracefully in conjunction with a plan. This enables rapid correction in the event of intrusion.

Business decisions (eg, budget, timeline) should not override security, which should be the priority. Attempting to close or obscure devices/protocols is not a solution and the so called security through obscurity, as a defensive measure, has long been rejected as inadequate (Shanon 1949).^12^ A balance between usability and security has to be struck carefully. Securing devices against attackers while keeping them open to clinicians is a difficult task. In mHealth, this difficulty can be amplified by the dependence on the patient’s devices (eg, smartphone) and practices, which are outside the control of a health care IT system. An example of an engineering compromise in implantable cardiac devices is the requirement for important wireless communications to only work at very short ranges. These communications could be made more secure but less usable (eg, requiring wires) or less secure but more usable (eg, using Bluetooth).

#### 7.2.3. Recommendations to Clinicians and Administrators

The organization should be designed with security in layers (also called defense in depth), where each system is protected with >1 layer of security. Hence, a breach in 1 layer will not necessarily result in total compromise. For example, a database may (1) require a password, (2) only grant a minimum level of access to each user, and (3) only accept internal connections. Thus, if a user’s password is compromised (1 failed), an attacker still cannot use it remotely. If the server is accidentally opened to remote access (3 failed), the attacker can still only access that one user’s data. Other innovative solutions include delegating security to a personal base station to use a novel radio design that can act as a jammer-cum-receiver (Gollakotta 2015).^13^

When recommending devices for patients, it is important to consider the potential privacy/security weaknesses compared with alternatives, ensure the patient is informed about these trade-offs, and review how the manufacturer has responded to security incidents in the past (Saxon 2018).^14^ However, the lack of outcome data, combined with the lack of documented real-world instances of actual cybersecurity intrusions to these devices or to peripheral products that support device connectivity (programmer, home communicator, database, and communication protocols), poses a difficult risk-benefit assessment for clinicians and patients alike.

Regulatory frameworks around cybersecurity are changing rapidly (Voelker 2018).^15^ The FDA (as well as other regulatory agencies worldwide) now includes security as a part of device safety/efficacy checks, and we encourage readers to report security issues to manufacturers and the government (eg, through FDA Medwatch; Shuren 2018).^16^

#### 7.2.4. Recommendations to Patients

Clear advice to patients concerning cybersecurity should be followed by a formal patient informed consent.

### 7.3. Reimbursement

Reimbursement is a powerful driver of adoption of new clinical pathways and typically instituted once an intervention has been proven scientifically valid and cost-effective (Treskes 2016).^17^ This process has only just started in mHealth and may be more complex to measure given the wide scope of telemedicine.

#### Reduced Costs

This technology may promote an effective means for early diagnosis and treatment of arrhythmias and associated comorbidities, leading to benefits of screening, prevention, and early treatment, thereby reducing adverse effects related to delayed therapy and utilization of costly health care resources (eg, ER visits or hospitalizations). mHealth may help individuals adhere to health recommendations, empower active participation in lifestyle changes to modify cardiovascular risk profile, and promote adherence to medical therapy (Feldman 2018).^18^ Together, these may reduce the burden of chronic disease and associated long-term disability. However, assessment of these longer term cost advantages is challenging, and value will vary according to country and health care system.

#### Increased Costs

Conversely, there are costs associated with administering mHealth programs. The widespread availability of smartphones and other commercially available mobile devices will generate a significant amount of inconclusive or false-positive findings, which will in turn lead to additional testing for validation, thereby increasing utilization of health care resources. Widespread implementation of screening programs would require additional consideration of costs related to detection of arrhythmias in currently unscreened populations. Health care providers will also be required to spend time reviewing and interpreting potentially voluminous results (and associated phone calls) before making additional evaluation and management decisions. This requires financial compensation to maintain a viable practice.

#### RM of Implanted Devices

This provides valuable experience. Randomized clinical trials conducted over many years that demonstrated safe and effective replacement of traditional in-clinic evaluations and more effective discovery of asymptomatic clinical events (Varma 2010).^19^ Health-economic studies like EuroEco (ICD patients) showed that clinic time needed for checking web-based information, telephone contacts, and in-clinic discussion when required was balanced by fewer planned in-office visits with RM, resulting in a similar cost for hospitals versus purely in-office follow-up (Heidbuchel 2014).^20^ From a payer perspective, there was a trend for cost-saving given fewer and shorter hospitalizations, seen also in other trials (Crossley 2011, Guedon-Moreau 2014, Hindricks 2014, Mabo 2012).^21–24^ However, in systems with fee-for-service reimbursement, less in-office visits (and hospitalizations) will lead to less income for the providers (ie, physicians and hospitals) without adaption of the new RM paradigm. This illustrates the complexities in reimbursement.

Currently, RM reimbursement (eg, the United States, Germany, France, and the United Kingdom) is implemented in a discrete way following the protocols of randomized trials like TRUST (Lumos-T Safely Reduces Routine Office Device Follow-Up) or IN-TIME (Implant-Based Multiparameter Telemonitoring of Patients With Heart Failure) (Hindricks 2014, Varma 2010, Varma 2010),^19,25,26^ with billing after demonstration of a remote contact, with a maximum number per year. Given the technological trend toward more continuous transmissions and decision support server systems that alert health care providers of potentially relevant information, possibly a subscription-based system providing a lump sum per year per followed patient may be more effective. This should cover costs of hardware, software, and other services (like potential use of third-party data monitoring centers) and would result in a much better prospective budgeting for both health care insurers and providers. This scheme may be apt for mobile technology.

It is anticipated that mHealth technology may provide a more efficient and cost-effective approach to health care delivery that could improve clinical workflow and enhance clinical care when integrated into clinical practice (Jiang 2019).^27^ Linking this to improved outcome will be an important driver of reimbursement, for example, for a process leading to an arrhythmia management decision (but not when monitoring the large asymptomatic population without risk factors). Ongoing studies evaluating mobile technology, such as use of a smartphone ECG for AF screening in the AF SMART II study (Atrial Fibrillation Screen, Management and Guideline Recommended Therapy), include a cost-effectiveness analysis (Orchard 2018).^28^ Responsibilities for reimbursement may extend beyond traditional parties in health care and drive novel pathways. Mobile device companies are clearly interested in reimbursement issues, evidenced by contact between Apple health executives and insurance companies (Bruining 2014).^29^ Initiatives undertaken in the United States are described in Appendix.

### 7.4. Regulatory Landscape for mHealth Devices

The pace of changes and improvement of digital technology is furiously fast. With the release and spread of the 5G cellular technology, this growth will probably be strengthened, and new frontiers around data streaming and associated analytics will be crossed. Unfortunately, this growth has been slower in the field of digital technologies, particularly in the United States. The reasons are probably linked to the unique relationship between the government and its health care system. In the United States, mHealth technologies are primarily led by private organizations operating under constraints linked to financial incentives (Centers for Medicare and Medicaid Services [CMS] reimbursement guidelines), patient privacy (Health Insurance Portability and Accountability Act), and patient safety (FDA). These constraints have become obsolete with the development of the digital health technologies and novel mHealth devices, and a new regulatory paradigm is being formed.

The FDA released an entirely new section under the Medical Device category called Digital Health, which is managed by the Center for Devices and Radiological Health (Shuren 2018, Center for Devices 2019).^30,31^ This development was triggered and supported by the 21st Century Cures Act signed into law on December 13, 2016. It is designed to help accelerate medical product development and bring new innovations and advances to patients. The FDA Digital Health policy is currently defined under 3 main categories: General Wellness, Mobile Medical Apps, and CDS Systems. mHealth devices are present in these 3 categories, which are defined as follows:

A wellness device is developed “for maintaining or encouraging a healthy lifestyle and is unrelated to the diagnosis, cure, mitigation, prevention, or treatment of a disease or condition” (21st Century Cures Act, Section 3060 (a)(o)(1)(B)). The FDA regulated Mobile Medical Apps on the other hand, as software that is focusing on traditionally regulated health functionalities and is categorized as software as a medical device (SaMD). The SaMD must be developed under well-defined frameworks involving specific software development life cycles (IEC-62304), risk assessment, reliability demonstration, and safety that includes cybersecurity. The CDS systems may rely on mHeath devices or be included in mHeath devices. The definitions of a CDS are provided in the 21 CCA, Section 520 (o)(1)(E). Briefly, they involve the presentation of medical data, recommendations to physicians about the prevention, diagnosis, or treatment of a condition or disease. It is not the intent that the HCP primarily relies on this information to make a clinical diagnosis or treatment decisions. If wellness devices do not require FDA approval to be commercialized, both SaMD and CDS do.

The regulatory policies are changing and adapting over time to fit the technology development of mHeath devices. But today, the time required for approving new technologies is significantly longer than the pace of change of the mHealth technologies. Hence, streamlining the regulatory submission process is of great interest to many stakeholders. One of the recent initiatives in the United States designed to address this challenge is the FDA’s digital health Software Precertification (Pre-CERT) program (Lee 2018).^32,33^ The Pre-CERT is developed to shift the current paradigm of SaMD submission. The program is ambitious and proposes to expedite regulatory review for the companies that can demonstrate a series of components that includes process certification, postmarket review, and real-world evidence (among others). It is expected that a company gaining FDA Pre-CERT could ultimately eliminate or streamline their regulatory submission process depending on the risk associated with their SaMD technologies. Started in 2019, this initiative currently involves international companies that are pushing their wellness technologies into the clinical realm. This type of new regulatory framework will certainly help corporate America to accelerate the commercialization of their products, but the Pre-CERT might be much more difficult to reach by smaller companies that do not have the resources to demonstrate the level of trust and to implement the level of verification and transparency Pre-CERT requires.

## 

References: Section 71.ISO/IEEE 11073-10103:2014. Health informatics — Point-of-care medical device communication — Part 10103: Nomenclature — Implantable device, cardiac.Accessed January 26, 2021. https://www.iso.org/standard/63904.html2.SlotwinerDJAbrahamRLAl-KhatibSMAndersonHVBunchTJFerraraMGLippmanNSerwerGASteinerPRTchengJE
HRS white paper on interoperability of data from cardiac implantable electronic devices (CIEDs).
Heart Rhythm. 2019;16:e107–e127. doi: 10.1016/j.hrthm.2019.05.0023107780110.1016/j.hrthm.2019.05.0023.ChowCKRedfernJHillisGSThakkarJSantoKHackettMLJanSGravesNde KeizerLBarryT
Effect of lifestyle-focused text messaging on risk factor modification in patients with coronary heart disease: a randomized clinical trial.
JAMA. 2015;314:1255–1263. doi: 10.1001/jama.2015.109452639384810.1001/jama.2015.109454.SpauldingEMMarvelFALeeMAYangWEDemoRWangJXunHShahLWengDFashanuOE
Corrie health digital platform for self-management in secondary prevention after acute myocardial infarction.
Circ Cardiovasc Qual Outcomes. 2019;12:e005509
doi: 10.1161/CIRCOUTCOMES.119.0055093104306510.1161/CIRCOUTCOMES.119.005509PMC66971675.RatwaniRMSavageEWillAArnoldRKhairatSMillerKFairbanksRJHodgkinsMHettingerAZ
A usability and safety analysis of electronic health records: a multi-center study.
J Am Med Inform Assoc. 2018;25:1197–1201. doi: 10.1093/jamia/ocy0882998254910.1093/jamia/ocy088PMC76468756.JalaliMSRussellBRazakSGordonWJ
EARS to cyber incidents in health care.
J Am Med Inform Assoc. 2019;26:81–90. doi: 10.1093/jamia/ocy1483051770110.1093/jamia/ocy148PMC76471587.KruseCSFrederickBJacobsonTMonticoneDK
Cybersecurity in healthcare: a systematic review of modern threats and trends.
Technol Health Care. 2017;25:1–10. doi: 10.3233/THC-1612632768956210.3233/THC-1612638.Mansfield-DevineS
Ransomware: taking businesses hostage.
Network Security. 20168–17. doi: 10.1016/S1353-4858(16)30096-49.Hospitals become major target for ransomware.
Network Security. 20161–2. doi: 10.1016/S1353-4858(16)30031-910.PerakslisED
Cybersecurity in health care.
N Engl J Med. 2014;371:395–397. doi: 10.1056/NEJMp14043582507583110.1056/NEJMp140435811.KlonoffDC
Cybersecurity for connected diabetes devices.
J Diabetes Sci Technol. 2015;9:1143–1147. doi: 10.1177/19322968155833342588316210.1177/1932296815583334PMC466732512.ShanonCE
28‘ Communication theory of secrecy systems’.
Bell System Technical Journal. 1949;26:656–715. doi: 10.1002/j.1538-7305.1949.tb00928.x13.GollakotaSHassaniehHRansfordBKatabiDFuK
They can hear your heartbeats: non-invasive security for implantable medical devices.
ACM SIGCOMM Comp Comm Rev. 2011;4Accessed January 26, 2021. https://dl.acm.org/doi/10.1145/2043164.2018438. doi: 10.1145/2043164.201843814.SaxonLAVarmaNEpsteinLMGanzLIEpsteinAE
Factors influencing the decision to proceed to firmware upgrades to implanted pacemakers for cybersecurity risk mitigation.
Circulation. 2018;138:1274–1276. doi: 10.1161/CIRCULATIONAHA.118.0347812974818810.1161/CIRCULATIONAHA.118.03478115.VoelkerR
FDA joins new effort to strengthen medical device cybersecurity.
JAMA. 2018;320:1970.10.1001/jama.2018.178343045847616.ShurenJPatelBGottliebS
FDA regulation of mobile medical apps.
JAMA. 2018;320:337–338. doi: 10.1001/jama.2018.88322997133910.1001/jama.2018.883217.TreskesRWvan der VeldeETBarendseRBruiningN
Mobile health in cardiology: a review of currently available medical apps and equipment for remote monitoring.
Expert Rev Med Devices. 2016;13:823–830. doi: 10.1080/17434440.2016.12182772747758410.1080/17434440.2016.121827718.FeldmanDITheodore RobisonWPacorJMCaddellLCFeldmanEBDeitzRLFeldmanTMartinSSNasirKBlahaMJ
Harnessing mHealth technologies to increase physical activity and prevent cardiovascular disease.
Clin Cardiol. 2018;41:985–991. doi: 10.1002/clc.229682967187910.1002/clc.22968PMC648988619.VarmaNEpsteinAEIrimpenASchweikertRLoveC; TRUST Investigators. Efficacy and safety of automatic remote monitoring for implantable cardioverter-defibrillator follow-up: the Lumos-T Safely Reduces Routine Office Device Follow-Up (TRUST) trial.
Circulation. 2010;122:325–332. doi: 10.1161/CIRCULATIONAHA.110.9374092062511010.1161/CIRCULATIONAHA.110.93740920.HeidbuchelHHindricksGBroadhurstPVan ErvenLFernandez-LozanoIRivero-AyerzaMMalinowskiKMarekARomero GarridoRFLöscherS
EuroEco (European Health Economic Trial on Home Monitoring in ICD Patients): a provider perspective in five European countries on costs and net financial impact of follow-up with or without remote monitoring.
Eur Heart J. 2015;36:158–169. doi: 10.1093/eurheartj/ehu3392517976610.1093/eurheartj/ehu339PMC429746921.CrossleyGHBoyleAVitenseHChangYMeadRH; CONNECT Investigators. The CONNECT (Clinical Evaluation of Remote Notification to Reduce Time to Clinical Decision) trial: the value of wireless remote monitoring with automatic clinician alerts.
J Am Coll Cardiol. 2011;57:1181–1189. doi: 10.1016/j.jacc.2010.12.0122125595510.1016/j.jacc.2010.12.01222.Guédon-MoreauLLacroixDSadoulNClémentyJKouakamCHermidaJSAliotEKacetS; ECOST Trial Investigators. Costs of remote monitoring vs. ambulatory follow-ups of implanted cardioverter defibrillators in the randomized ECOST study.
Europace. 2014;16:1181–1188. doi: 10.1093/europace/euu0122461457210.1093/europace/euu012PMC411433023.HindricksGTaborskyMGliksonMHeinrichUSchumacherBKatzABrachmannJLewalterTGoetteABlockM; IN-TIME Study Group*. Implant-based multiparameter telemonitoring of patients with heart failure (IN-TIME): a randomised controlled trial.
Lancet. 2014;384:583–590. doi: 10.1016/S0140-6736(14)61176-42513197710.1016/S0140-6736(14)61176-424.MaboPVictorFBazinPAhresSBabutyDDa CostaABinetDDaubertJC; COMPAS Trial Investigators. A randomized trial of long-term remote monitoring of pacemaker recipients (the COMPAS trial).
Eur Heart J. 2012;33:1105–1111. doi: 10.1093/eurheartj/ehr4192212741810.1093/eurheartj/ehr419PMC334163025.HindricksGTaborskyMGliksonMHeinrichUSchumacherBKatzABrachmannJLewalterTGoetteABlockM; IN-TIME Study Group*. Implant-based multiparameter telemonitoring of patients with heart failure (IN-TIME): a randomised controlled trial.
Lancet. 2014;384:583–590. doi: 10.1016/S0140-6736(14)61176-42513197710.1016/S0140-6736(14)61176-426.VarmaNMichalskiJEpsteinAESchweikertR
Automatic remote monitoring of implantable cardioverter-defibrillator lead and generator performance: the Lumos-T Safely Reduces Routine Office Device Follow-Up (TRUST) trial.
Circ Arrhythm Electrophysiol. 2010;3:428–436. doi: 10.1161/CIRCEP.110.9519622071671710.1161/CIRCEP.110.95196227.JiangXMingWKYouJH
The cost-effectiveness of digital health interventions on the management of cardiovascular diseases: systematic review.
J Med Internet Res. 2019;21:e13166
doi: 10.2196/131663121013610.2196/13166PMC660125728.OrchardJJNeubeckLFreedmanBWebsterRPatelAGallagherRLiJHespeCMFergusonCZwarN
Atrial Fibrillation Screen, Management And Guideline Recommended Therapy (AF SMART II) in the rural primary care setting: an implementation study protocol.
BMJ Open. 2018;8:e023130
doi: 10.1136/bmjopen-2018-02313010.1136/bmjopen-2018-023130PMC62527583038544429.BruiningNCaianiEChronakiCGuzikPvan der VeldeE; Task Force of the e-Cardiology Working. Acquisition and analysis of cardiovascular signals on smartphones: potential, pitfalls and perspectives: by the Task Force of the e-Cardiology Working Group of European Society of Cardiology.
Eur J Prev Cardiol. 2014;21suppl 24–13. doi: 10.1177/204748731455260410.1177/20474873145526042535494830.ShurenJPatelBGottliebS
FDA regulation of mobile medical apps.
JAMA. 2018;320:337–338. doi: 10.1001/jama.2018.88322997133910.1001/jama.2018.883231.Center for Devices, Radiological Health. Digital Health.
5
12, 2019
U.S. Food and Drug Administration
[cited January 20, 2020]. Accessed January 26, 2021. https://www.fda.gov/medical-devices/digital-health-center-excellence32.U.S. Food and Drug Administration. PRE-CERT.
https://www.fda.gov/medical-devices/digital-health-software-precertification-pre-cert-program/precertification-pre-cert-pilot-program-milestones-and-next-steps33.LeeTTKesselheimAS
U.S. Food and Drug Administration precertification pilot program for digital health software: weighing the benefits and risks.
Ann Intern Med. 2018;168:730–732. doi: 10.7326/M17-27152963295310.7326/M17-2715

## 8. Predictive Analytics

AI is a broad term that describes any computational programs that normally require human intelligence such as image perception, pattern recognition, inference, or prediction (www.oed.com; Kagiyama 2019).^1,2^ Most commonly, AI is implemented using analytical methods of machine learning or deep learning. These methods are well suited for pattern classifications, such as images, including ECG.

The potential synergy between AI and mHealth has excited the health care community since this may enable solutions to improve patient outcomes and increase efficiency with reduced costs in health care (Davenport 2019, Marcolino 2018).^3,4^ Smartphone apps and wearable devices generate a huge amount of data that exceed the human capacity of integration and interpretation (Steinhubl 2015).^5^ Biometric datasets of astronomical proportions may be compiled. This knowledge may be directed to treat an individual or understand populations. For instance, 6 billion nights of surrogate sleep data reflecting global sleep deprivation may potentially inform public health initiatives (https://aasm.org/fitbit-scientists-reveal-results-analysis-6-billion-nights-sleep-data).^6^ mHealth with internet connection enables cloud-based predictive analytics from individual-level information (Bumgarner 2018, Nascimento 2018, Ribeiro 2019).^7–9^

Cardiology has been an early area of investigation in AI due to the abundance of data well suited for classification and prediction (Seetharam 2019).^10^ Neural networks have been tested, trained, and successfully validated to be at least as accurate, if not more, than physicians in diagnosis or classification of 12-lead ECGs and recognition of arrhythmias in rhythm strips and ambulatory ECG recordings (Hannun 2019, Ribeiro 2019, Smith 2019).^11–13^ They have also been shown to successfully estimate ejection fraction, identify left ventricular dysfunction, and even diagnose diseases such as hypertrophic cardiomyopathy from the echocardiogram (Zhang 2018).^14^ More recently, neural networks have also aided in gathering new dimensions of information, such as identifying left ventricular dysfunction (Attia 2019).^15^ These methods have the potential for a point-of-use diagnosis of a wearable sensor or consumer device and without delays of requiring clinical conformation, although rigorous safety assessments of unsupervised use will be necessary. More recently, AI methods have also been applied to prediction, not just classification, for example, using 12-lead ECG to predict the risk of AF from a sinus rhythm ECG (Attia 2019).^16^

Already, AI has been embedded in mHealth applications, such as smartwatch and smartphone-connect ECG for semiautomated diagnosis of arrhythmias (Bumgarner 2018, Halcox 2017).^7,17^ These diagnoses are intended to serve as prediagnostics rather than supplanting a physician interpretation. Application of AI techniques to point-of-care ultrasound in the development of machine-learning systems may aid in the optimization of acquisition and interpretation of a high volume of images, reduce variability, and improve diagnostic accuracy (Chamsi-Pasha 2017).^18^ AI-based prediction models have been developed for HF and AF, although sometimes the accuracy of the AI-derived models seems to be rather limited or not superior than those derived from conventional methods (Awan 2019, Clifton 2015, Frizzel 2017, Goto 2019, Safavi 2019, Tripoliti 2019).^19–24^ mHealth-specific investigations are few. Results from the LINK-HF study were encouraging. A cloud-based analytics platform used a general machine-learning method of similarity-based modeling, which models the behavior of complex systems (eg, aircraft engines) to create a predictive algorithm for HF decompensation, using data streamed from a chest patch sensor.

Several limitations should be considered and roadblocks removed before AI-based mHealth strategies become routinely incorporated in clinical practice (Kagiyama 2019, Powell 2019, Ribeiro 2019, Steinhubl 2015).^2,25–27^ Studies on AI are still scarce and based on observational studies and secondary datasets. Validation in other clinical settings and a deeper evaluation of their meaning in every day practice are generally lacking. Thus, high-quality evidence that supports the adoption of many new technologies is not available. Most algorithms work with the black-box principle, without allowing the user to know the reasons why a diagnosis or recommendation was generated, which can be a problem, especially if the algorithms were designed for a different environment than the one that the current patient is inserted (Weng 2017, Ribeiro 2019).^12,28^ Issues regarding cost-effectiveness, implementation, ethics, privacy, and safety are still unsolved.

## 

References: Section 81.“Artificial Intelligence.”
Oxford English Dictionary
Accessed January 26, 2021. https://www.oed.com/2.KagiyamaNShresthaSFarjoPDSenguptaPP
Artificial intelligence: practical primer for clinical research in cardiovascular disease.
J Am Heart Assoc. 2019;8:e012788
doi: 10.1161/JAHA.119.0127883145099110.1161/JAHA.119.012788PMC67558463.DavenportTKalakotaR
The potential for artificial intelligence in healthcare.
Future Healthc J. 2019;6:94–98. doi: 10.7861/futurehosp.6-2-9410.7861/futurehosp.6-2-94PMC6616181313635134.MarcolinoMSOliveiraJAQD’AgostinoMRibeiroALAlkmimMBMNovillo-OrtizD
The impact of mHealth interventions: systematic review of systematic reviews.
JMIR Mhealth Uhealth. 2018;6:e23
doi: 10.2196/mhealth.88732934346310.2196/mhealth.8873PMC57926975.SteinhublSRMuseEDTopolEJ
The emerging field of mobile health.
Sci Transl Med. 2015;7:283rv3
doi: 10.1126/scitranslmed.aaa348710.1126/scitranslmed.aaa3487PMC4748838258778946.PogueD
Yahoo! Finance. Exclusive: what Fitbit’s 6 billion nights of sleep data reveals about us.
Accessed January 31, 2020. https://finance.yahoo.com/news/exclusive-fitbits-6-billion-nights-sleep-data-reveals-us-110058417.html7.BumgarnerJMLambertCTHusseinAACantillonDJBaranowskiBWolskiKLindsayBDWazniOMTarakjiKG
Smartwatch algorithm for automated detection of atrial fibrillation.
J Am Coll Cardiol. 2018;71:2381–2388. doi: 10.1016/j.jacc.2018.03.0032953506510.1016/j.jacc.2018.03.0038.NascimentoBRBeatonAZNunesMCPTompsettAROliveiraKKBDiamantinoACBarbosaMMLourençoTVTeixeiraIMRuizGZL; PROVAR+ (Programa de Rastreamento da Valvopatia Reumática and Other Cardiovascular Diseases) Investigators. Integration of echocardiographic screening by non-physicians with remote reading in primary care.
Heart. 2019;105:283–290. doi: 10.1136/heartjnl-2018-3135933018120210.1136/heartjnl-2018-3135939.RibeiroALPPaixãoGMMGomesPRRibeiroMHRibeiroAHCanazartJAOliveiraDMFerreiraMPLimaEMMoraesJL
Tele-electrocardiography and bigdata: the CODE (Clinical Outcomes in Digital Electrocardiography) study.
J Electrocardiol. 2019;57S:S75–S78. doi: 10.1016/j.jelectrocard.2019.09.0083152657310.1016/j.jelectrocard.2019.09.00810.SeetharamKKagiyamaNSenguptaPP
Application of mobile health, telemedicine and artificial intelligence to echocardiography.
Echo Res Pract. 2019;6:R41–R52. doi: 10.1530/ERP-18-00813084475610.1530/ERP-18-0081PMC643297711.HannunAYRajpurkarPHaghpanahiMTisonGHBournCTurakhiaMPNgAY
Cardiologist-level arrhythmia detection and classification in ambulatory electrocardiograms using a deep neural network.
Nat Med. 2019;25:65–69. doi: 10.1038/s41591-018-0268-33061732010.1038/s41591-018-0268-3PMC678483912.RibeiroAHRibeiroMHPaixãoGMMOliveiraDMGomesPRCanazartJAFerreiraMPSAnderssonCRMacfarlanePWMeiraWJr
Automatic diagnosis of the 12-lead ECG using a deep neural network.
Nat Commun. 2020;11:1760
doi: 10.1038/s41467-020-15432-43227351410.1038/s41467-020-15432-4PMC714582413.SmithSWWalshBGrauerKWangKRapinJLiJFennellWTabouletP
A deep neural network learning algorithm outperforms a conventional algorithm for emergency department electrocardiogram interpretation.
J Electrocardiol. 2019;52:88–95. doi: 10.1016/j.jelectrocard.2018.11.0133047664810.1016/j.jelectrocard.2018.11.01314.ZhangJGajjalaSAgrawalPTisonGHHallockLABeussink-NelsonLLassenMHFanEArasMAJordanC
Fully automated echocardiogram interpretation in clinical practice.
Circulation. 2018;138:1623–1635. doi: 10.1161/CIRCULATIONAHA.118.0343383035445910.1161/CIRCULATIONAHA.118.034338PMC620038615.AttiaZIKapaSLopez-JimenezFMcKiePMLadewigDJSatamGPellikkaPAEnriquez-SaranoMNoseworthyPAMungerTM
Screening for cardiac contractile dysfunction using an artificial intelligence-enabled electrocardiogram.
Nat Med. 2019;25:70–74. doi: 10.1038/s41591-018-0240-23061731810.1038/s41591-018-0240-216.AttiaZINoseworthyPALopez-JimenezFAsirvathamSJDeshmukhAJGershBJCarterREYaoXRabinsteinAAEricksonBJ
An artificial intelligence-enabled ECG algorithm for the identification of patients with atrial fibrillation during sinus rhythm: a retrospective analysis of outcome prediction.
Lancet. 2019;394:861–867. doi: 10.1016/S0140-6736(19)31721-03137839210.1016/S0140-6736(19)31721-017.HalcoxJPJWarehamKCardewAGilmoreMBarryJPPhillipsCGravenorMB
Assessment of remote heart rhythm sampling using the AliveCor heart monitor to screen for atrial fibrillation: the REHEARSE-AF Study.
Circulation. 2017;136:1784–1794. doi: 10.1161/CIRCULATIONAHA.117.0305832885172910.1161/CIRCULATIONAHA.117.03058318.Chamsi-PashaMASenguptaPPZoghbiWA
Handheld echocardiography: current state and future perspectives.
Circulation. 2017;136:2178–2188. doi: 10.1161/CIRCULATIONAHA.117.0266222918049510.1161/CIRCULATIONAHA.117.02662219.AwanSEBennamounMSohelFSanfilippoFMDwivediG
Machine learning-based prediction of heart failure readmission or death: implications of choosing the right model and the right metrics.
ESC Heart Fail. 2019;6:428–435. doi: 10.1002/ehf2.124193081029110.1002/ehf2.12419PMC643744320.CliftonDANiehausKECharltonPColopyGW
Health informatics via machine learning for the clinical management of patients.
Yearb Med Inform. 2015;10:38–43. doi: 10.15265/IY-2015-0142629384910.15265/IY-2015-014PMC458706521.FrizzellJDLiangLSchultePJYancyCWHeidenreichPAHernandezAFBhattDLFonarowGCLaskeyWK
Prediction of 30-day all-cause readmissions in patients hospitalized for heart failure: comparison of machine learning and other statistical approaches.
JAMA Cardiol. 2017;2:204–209. doi: 10.1001/jamacardio.2016.39562778404710.1001/jamacardio.2016.395622.GotoSGotoSPieperKSBassandJPCammAJFitzmauriceDAGoldhaberSZHaasSParkhomenkoAOtoA; GARFIELD-AF Investigators. New artificial intelligence prediction model using serial prothrombin time international normalized ratio measurements in atrial fibrillation patients on vitamin K antagonists: GARFIELD-AF.
Eur Heart J Cardiovasc Pharmacother. 2020;6:301–309. doi: 10.1093/ehjcvp/pvz0763182148210.1093/ehjcvp/pvz076PMC755681123.SafaviKCKhaniyevTCopenhaverMSeelenMZenteno LangleACZangerJDailyBLeviRDunnP
Development and validation of a machine learning model to aid discharge processes for inpatient surgical care.
JAMA Netw Open. 2019;2:e1917221
doi: 10.1001/jamanetworkopen.2019.172213182550310.1001/jamanetworkopen.2019.17221PMC699119524.TripolitiEEKaranasiouGSKalatzisFGBechlioulisAGoletsisYNakaKFotiadisDI
HEARTEN KMS - a knowledge management system targeting the management of patients with heart failure.
J Biomed Inform. 2019;94:103203
doi: 10.1016/j.jbi.2019.1032033107145510.1016/j.jbi.2019.10320325.PowellJ
Trust me, I’m a chatbot: how artificial intelligence in health care fails the turing test.
J Med Internet Res. 2019;21:e16222
doi: 10.2196/162223166108310.2196/16222PMC691423626.RibeiroALOliveiraGMM
Toward a patient-centered, data-driven cardiology.
Arq Bras Cardiol. 2019;112:371–373. doi: 10.5935/abc.201900693099471410.5935/abc.20190069PMC645942827.SteinhublSRMuseEDTopolEJ
The emerging field of mobile health.
Sci Transl Med. 2015;7:283rv3
doi: 10.1126/scitranslmed.aaa348710.1126/scitranslmed.aaa3487PMC47488382587789428.WengSFRepsJKaiJGaribaldiJMQureshiN
Can machine-learning improve cardiovascular risk prediction using routine clinical data?
PLoS One. 2017;12:e0174944
doi: 10.1371/journal.pone.01749442837609310.1371/journal.pone.0174944PMC5380334

## 9. Future Directions

**Figure 7.**
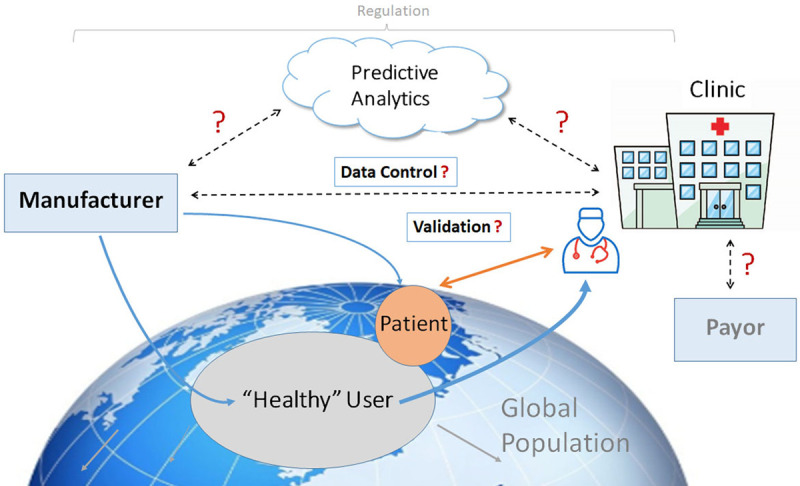
**Connectivity and questions.** Multiple levels of cooperation among a variety of stakeholders are needed to capitalize fully on the vast potential of mobile health (mHealth), but many questions remain unanswered. Healthy consumers (increasing) predominate among mHealth users. Only a minority of patients are prescribed these digital tools. Potential health benefits of mHealth may be realized when manufacturer participates with clinic for validation in defined disease states. Parties responsible for data control, and thereby predictive analytics, need to be defined. Ultimately, the payor and physician need to be convinced of benefits before digital tools are firmly embedded in clinical practice.

mHealth is disruptive at multiple levels of health care but requires significant investment in validation, demonstration of clinical utility, and value. Stakeholders, each with independent concerns and constraints (Table 5), lack consensus or coordination with design, use cases, and implementation (Figure 7). Thus, formal recommendations for integration of mHealth into clinical practice cannot be made at this time. This is exemplified by the US Preventative Services Task Forces statement that “evidence is insufficient to initiate therapy for AF detected by mHealth,” despite the fact that AF has been an early use case with strong patient and clinician interest (Curry 2018).^1^ Thus, mHealth devices are currently nonprescription devices marketed directly to consumers to track data without enabling interventions.

**Table 5.**
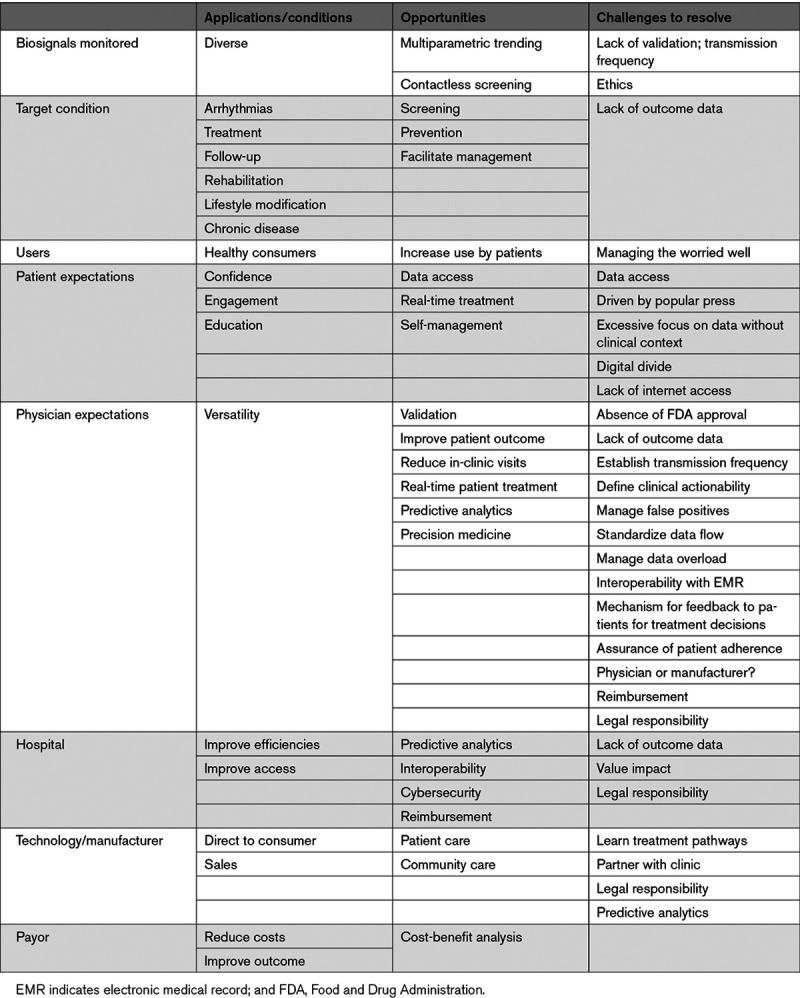
Conditions, Stakeholders, and Expectations

Some of the steps needed to standardize mHealth applications are outlined below.

### 1. Validation

#### Promote Standards and Create Tools for the Comparative Assessment of Functionality, Relative to a Medical Use Device

Results from different devices applied to the same condition may not match: for example, the diagnosis of AF by ECG- or photoplethysmography-based systems is made differently. This has significant implications for medical decisions.

### 2. Identify Clinical Care Pathways

#### Screening

- Assess value according to the population addressed.
- Establish a uniform set of criteria for clinical actionability (Slotwiner 2019).^2^

Screening should be medically directed and not driven by commercial interests. Caution should be exercised in extrapolating management strategies learned from cohorts with clinically diagnosed AF (usually from health care system data, trials, or inpatient registries) to AF detected with mHealth technologies (healthy consumers). Data from low-risk populations carry a relatively high risk of false positives, which may generate additional tests with resultant clinical risk to patient (even inducing anxiety rather than reassurance), risk from overtreatment, and costs to the payor. There is a risk that unless directed to a higher risk population, screening for AF using mHealth technologies may fail and follow the trajectory of many medical screening programs throughout history.

##### Key Knowledge Gap

Identify characteristics (duration, episode number/density) and risk factors that justify anticoagulation for mHealth-detected AF.

#### Disease Management

- Identify conditions and schedules for home-based therapeutic strategies that may reduce dependency on clinic evaluations (as shown for CIEDs).
- Identify signals that predict decompensation and design preemptive interventions.
- Assess efficacy of therapies.

#### Outcomes

Evidence for benefit of mHealth directed

- arrhythmia treatment
- management of modulating factors (eg, comorbidities and lifestyle modifications).

### 3. Implementation

#### Cost Effectiveness

For example, impact of improved clinical workflow and enhance clinical care, according to condition (Jiang 2019).^3^
Impact on health care system and reimbursement.
Impact on costs to patient or consumer.

#### Public Health and Professional Society Initiatives

Education, awareness
Bring together stakeholders
Guidelines

### 4. Patient Self-Management

Patients control the intensity of monitoring and act on patient-facing data. Frequency of data acquisition is sporadic determined by, for example, convenience, or following symptoms, or recreational. This strategy is likely insensitive for events and rarely delivers rapid clinical actionability for life-threatening conditions. What is required is

- education on which data are clinically actionable in individual’s clinical context and
- tailor monitoring schedule accordingly
- proof of safety

One recent example illustrates an on-demand use. The Fibricheck app was utilized by patients to monitor rate and rhythm for a week before teleconsultations during the COVID-19 pandemic to enable remote assessment of the disease state and support treatment decisions. This was regulated by a time-limited prescription to use the app for a predefined period, avoiding unnecessary data load and additional follow-up patient contacts (Pluymaekers 2020).^4^

- Patients’ legal right to their medical data to include data collected from nonmedical (ie, consumer) products.

### 5. Manufacturer

mHealth introduces the manufacturer as a party with significant responsibilities. mHealth tools largely have been developed as consumer-facing technologies accessible to a broader market through retail channels rather than through established medical supply channels. This may make business sense for the technology supplier, given the high community penetration of wearable, smart-technology devices (1 in 10 Americans [30 million total]). However, a direct-to-consumer health care delivery bypasses both the clinician, health care system, and insurer, without addressing the needs of health professionals, who remain responsible for clinical decision-making on acquired data. Any advance toward medical application (beyond toys for the worried well/wealthy well) will require manufacturers to

- facilitate accessibility and affordability
- engage with clinicians to engineer devices according to clinical needs and partner in validation. This is vital, since physician carries ultimate responsibility for medical decisions and is best positioned to guide development and application
- define role as data controllers (eg, GDPR in Europe).

### 6. Assign Responsibilities

- Identify parties (manufacturer, hospital, or third party) responsible for cybersecurity, data protection, and liability for misdiagnosis or missed diagnosis
- Define standard of care for clinic response time according to condition

This assumes greater significance as clinical decisions become enabled in real-time using cloud-processing resources linked to enhanced data transmission rates (5G) and InternetofThings and scalability increases.

- Ethical and societal issues with multiple screening (Yan 2019, Turakhia 2020).^5,6^

### 7. Health Care Delivery

Interconnectedness between individual applications and with existing health care architectures may reshape the current environment.

- Exception-based ambulatory care, that is, see patients as they need to be seen.
- Centralized (cloud)-based processing to forward only clinically relevant data to physician/clinic.
- Identify at-risk patients early (even before symptoms develop) and permit preemptive care (Boehmer 2017, Rosier 2016).^7,8^
- Pooled population screening, altering the paradigm of individual screening (Yan 2019, Turakhia 2020).^5,6^
- Extend the role of wearables from ambulatory to in-hospital care, for example, replace traditional wired monitoring of single parameters for individual analysis to wireless monitoring of multiple parameters.

For example, a waterproof ring technology (BodiMetrics) was used for multiparametric monitoring (HR, sleep, oxygen desaturation index, steps, and calories burned) in intensive care unit management for COVID-19 patients. The ring links to a smartphone or centralized hub in hospitals and permits data sharing and cooperative treatment (https://bodimetrics.com/product/circul-sleep-and-fitness-ring/).^9^

- Extend function from monitoring only to intervention.
  - Enable remote programming of therapeutic implantable devices

For example, CIEDs, emerging wearable cardioverter-defibrillators, are incorporating smartphone Bluetooth Low Energy–based connectivity for the transmission, display, and interpretation of transmitted data by patients and their clinicians. This may permit reprogramming of parameters like diagnostic data, detection zones, clearing counters; AV delays/post-ventricular atrial refractory period adjustment, upper rate and lower rate adjustments, reprogram amplitude adjustments; magnetic resonance imaging mode, and enable emergency therapies or disable inappropriate therapies due to lead fracture/incessant supraventricular tachycardia/double counting.

- Enable interventional procedures, for example, telerobotic ablation models, which could improve access to patients living in remote areas with highly skilled EPs operating remotely (Choi 2018, Haidegger 2011, Shinoda 2020).^10–12^

- Enable precision medicine by incorporation of the wider range of mobile signals seamlessly into genetic and clinical profile, with environmental and lifestyle data (big data; https://ghr.nlm.nih.gov/primer/precisionmedicine/initiative).^13^

#### Concluding Remarks

mHealth application is at different stages of evolution around the world. Few of the technologies described are universally approved and affordable in all countries. As a result, this document reflects largely US perspectives. The experience described may serve to guide other members of the international professional bodies endorsing this consensus statement. The World Health Organization envisioned that increasing the capacity to implement and scale up cost-effective innovative digital health could play a major role toward achieving universal health coverage and ensuring access to quality health services, at the same time recognizing barriers similar to those discussed here. Some of these can be resolved rapidly, as seen in response to the recent SARS-CoV-2 global pandemic, which forced a need for contactless monitoring and thereby adoption of digital tools (U.S. Department of Health & Human Services 2020, U.S. Food & Drug Administration 2020, Varma 2020).^14–16^ Regulatory bodies were responsive, approving technologies, relaxing rules confining the use of telehealth services within borders and to certain patient populations, and creating a reimbursement structure, illustrating that appropriate solutions can be created when necessary.

Demonstration of the clinical utility of mHealth has the potential to revolutionize how populations interact with health services, worldwide.

## 

References: Section 91.CurrySJKristAHOwensDKBarryMJCaugheyABDavidsonKWDoubeniCAEplingJWJrKemperARKubikM
US Preventive Services Task Force, screening for atrial fibrillation with electrocardiography: US Preventive Services Task Force recommendation statement.
JAMA. 2018;320:478–484.3008801610.1001/jama.2018.103212.SlotwinerDJAbrahamRLAl-KhatibSMAndersonHVBunchTJFerraraMGLippmanNSerwerGASteinerPRTchengJE
HRS white paper on interoperability of data from cardiac implantable electronic devices (CIEDs).
Heart Rhythm. 2019;16:e107–e127. doi: 10.1016/j.hrthm.2019.05.0023107780110.1016/j.hrthm.2019.05.0023.JiangXMingWKYouJH
The cost-effectiveness of digital health interventions on the management of cardiovascular diseases: systematic review.
J Med Internet Res. 2019;21:e13166
doi: 10.2196/131663121013610.2196/13166PMC66012574.PluymaekersNAHAHermansANLvan der VeldenRMJden UijlDWVorstermansBBuskesSHendriksJMVernooyKCrijnsHJGMLinzD
On-demand app-based rate and rhythm monitoring to manage atrial fibrillation through teleconsultations during COVID-19.
Int J Cardiol Heart Vasc. 2020;28:100533
doi: 10.1016/j.ijcha.2020.1005333239141210.1016/j.ijcha.2020.100533PMC72056265.YanBPLaiWHSChanCKYAuACKFreedmanBPohYCPohMZ
High-throughput, contact-free detection of atrial fibrillation from video with deep learning.
JAMA Cardiol. 2020;5:105–107. doi: 10.1001/jamacardio.2019.40043177446110.1001/jamacardio.2019.4004PMC69021236.TurakhiaMP
Diagnosing with a camera from a distance-proceed cautiously and responsibly.
JAMA Cardiol. 2020;5:107
doi: 10.1001/jamacardio.2019.457210.1001/jamacardio.2019.4572317744487.BoehmerJPHariharanRDevecchiFGSmithALMolonGCapucciAAnQAverinaVStolenCMThakurPH
A multisensor algorithm predicts heart failure events in patients with implanted devices: results from the MultiSENSE Study.
JACC Heart Fail. 2017;5:216–225. doi: 10.1016/j.jchf.2016.12.0112825412810.1016/j.jchf.2016.12.0118.RosierAMaboPTemalLVan HillePDameronODelégerLGrouinCZweigenbaumPJacquesJChazardE
Personalized and automated remote monitoring of atrial fibrillation.
Europace. 2016;18:347–352. doi: 10.1093/europace/euv2342648767010.1093/europace/euv2349.BodiMetrics. BodiMetrics^TM^ CIRCUL Sleep & Fitness Ring.
Accessed January 26, 2021. https://bodimetrics.com/product/circul-sleep-and-fitness-ring/10.ChoiPJOskouianRJTubbsRS
Telesurgery: past, present, and future.
Cureus. 2018;10:e2716
doi: 10.7759/cureus.27163007928210.7759/cureus.2716PMC606781211.HaideggerTSándorJBenyóZ
Surgery in space: the future of robotic telesurgery.
Surg Endosc. 2011;25:681–690. doi: 10.1007/s00464-010-1243-32065232010.1007/s00464-010-1243-312.ShinodaYSatoAAdachTNishiINogamiAAonumaKIedaM
Early clinical experience of radiofrequency catheter ablation using an audiovisual telesupport system.
Heart Rhythm. 2020;175pt B870–875. doi: 10.1016/j.hrthm.2020.01.0183235445210.1016/j.hrthm.2020.01.01813.What is the Precision Medicine Initiative?Accessed January 26, 2021. https://medlineplus.gov/genetics/understanding/precisionmedicine/initiative/14.U.S. Department of Health & Human Services. Notification of enforcement discretion for telehealth remote communications during the COVID-19 nationwide public health emergency.
2020
Accessed April 19, 2020. https://www.hhs.gov/hipaa/for-professionals/special-topics/emergency-preparedness/notification-enforcement-discretiontelehealth/index.html?fbclid=IwAR1K7DQLYr6noNgWA6bMqK74orWPv_C_aghKz19au-BNoT0MdQyg-3E8DWI#15.U.S. Food & Drug Administration. Enforcement policy for non-invasive remote monitoring devices used to support patient monitoring during the coronavirus disease-2019 (COVID-19) public health emergency.
2020
Accessed April 27, 2020. https://www.fda.gov/regulatory-information/search-fda-guidance-documents/enforcement-policy-non-invasive-remote-monitoring-devices-used-support-patient-monitoring-during16.VarmaNMarroucheNFAguinagaLAlbertCMArbeloEChoiJIChungMKConteGDagherLEpsteinLM
HRS/EHRA/APHRS/LAHRS/ACC/AHA worldwide practice update for telehealth and arrhythmia monitoring during and after a pandemic.
J Am Coll Cardiol. 2020;76:1363–1374. doi: 10.1016/j.jacc.2020.06.0193253493610.1016/j.jacc.2020.06.019PMC7289088

## Sources of Funding

None.

## Disclosures

For Disclosures, see the Data Supplement.

## Supplementary Material



## Appendix

In the United States, reimbursement for medical services is guided primarily by the Centers for Medicare and Medicaid Services (CMS). The American Medical Association Current Procedural Terminology (CPT) Committee develops descriptive codes for each medical service and assigns a CPT code. Each CPT code is then referred to the association’s Relative Value Update Committee to develop a recommended relative value unit, which determines reimbursement. CMS usually accepts the recommendations from the American Medical Association. Presently, the CPT Committee is developing codes to represent the clinical work involved in managing mobile health data. These codes will then be evaluated and assigned relative value unit values. If accepted by CMS, these will be included in the Medicare Fee Schedule and go into clinical use. This process typically takes 2 years. Once the codes and services are approved by CMS and published in the Fee Schedule, other insurers typically accept them as well (at the time of writing, CPT 99091 and CPT 99457 had received approval). In 2015, the CMS in the United States initiated a new chronic care management code that reimburses primary care practices for non–face-to-face care for chronic care management payment. In November 2018, CMS finalized plans to reimburse health care providers for certain remote patient monitoring and telehealth services. These changes focused on 3 new CPT codes that separate remote patient management services from telehealth services (ref 8 web-based Department of Health).1 The new CPT codes include No. 99453, 99454, and 99457. The first 2 codes describe RM of physiological parameters but do not specifically include ECG monitoring. The third code provides management services, 20 minutes or more of clinical staff/physician/other qualified health care professional time in a calendar month requiring interactive communication with the patient/caregiver during the month; however, it is not clear that this code could be utilized for ECG monitoring services through mobile devices. The preexisting CPT code 93040 (used for reporting on a rhythm ECG, 1–3 leads, without interpretation and report) would not be appropriate for patientinitiated mobile device events as this would require an order that is triggered by an event followed by a separate signed and retrievable report. CMS has also proposed establishing a new virtual service HCPCS (Healthcare Common Procedure Coding System) code, GRAS1, for “Remote Evaluation of Pre-Recorded Patient Information,” which would reimburse for a provider’s asynchronous review of “recorded video and/or images captured by a patient in order to evaluate the patient’s condition” and determine whether or not an office visit is necessary (ref 9 web-based Telemedicine and Health).2 This code could be billed separately if there was not an E/M visit within the previous 7 days. CMS finalized separate payment for CPT code 99091 (collection and interpretation of physiological data; eg, ECG, BP, and glucose monitoring) digitally stored or transmitted by the patient or caregiver to the physician or other qualified health care professional, qualified by education, training, licensure/regulation, requiring a minimum of 30 minutes of time (ref 8 web based).1 However, there must be a clinically relevant reason for the physician to need to review the data each month.
