# Supplementary material for: 2021 ISHNE/HRS/EHRA/APHRS Expert Collaborative Statement on mHealth in Arrhythmia Management: Digital Medical Tools for Heart Rhythm Professionals: From the International Society for Holter and Noninvasive Electrocardiology/Heart Rhythm Society/European Heart Rhythm Association/Asia-Pacific Heart Rhythm Society
Source: Circ Arrhythm Electrophysiol. 2021 Feb 12;14(2):e009204. doi: 10.1161/CIRCEP.120.009204 (PMC7892205; doi:10.1161/CIRCEP.120.009204)
Supplement: Supplementary file 1 [file hae-14-e009204-s001.pdf]

WILEY

19th October 2020

To whom it may concern,

Re: Letter of understanding for "The Article" entitled: '2020 ISHNE/ HRS/ EHRA/ APHRS collaborative statement on mHealth in Arrhythmia Management: Digital Medical Tools for Heart Rhythm Professionals'.

Terms of agreement

The Article is to be co-published in *Annals of Noninvasive Electrocardiology* (published by Wiley Periodicals Inc. on behalf of the International Society for Holter and Noninvasive Electrocardiology)*, Cardiovascular Digital Health Journal* (published by Elsevier Inc. on behalf of the Heart Rhythm Society), *Circulation: Arrhythmia and Electrophysiology* (published by Wolters Kluwer Health, Inc. on behalf of the American Heart Association, Inc.), *European Heart Journal - Digital Health* (published by Oxford University Press on behalf of the European Society of Cardiology) and *Journal of Arrhythmia* (published by John Wiley and Sons Australia, Ltd on behalf of the Japanese Heart Rhythm Society and the Asia Pacific Heart Rhythm Society).

The purpose of this letter of understanding is to confirm agreement to the copyright terms and rights for the joint publication of the Article.

By signing this letter, the International Society for Holter and Noninvasive Electrocardiology warrants that it has all the necessary rights under this Agreement. The International Society for Holter and Noninvasive Electrocardiology will grant to the Heart Rhythm Society, the American Heart Association, the European Society of Cardiology, the Japanese Heart Rhythm Society and the Asia Pacific Heart Rhythm Society the right to co-publish the Article on a CC-BY license. Each party agrees to publish this paper under an Open Access CC-BY license and may waive any open access fees at their discretion. Each party warrants that any material originating with each party will not infringe upon or violate any copyright, trademark, obligation of confidentiality or any other right on the privacy of others, or contain any libellous matter or material or matter or instructions that may cause harm or injury.

By signing this letter, the parties agree that each party will have the right to:

- publish the final version of the Article in their journals: *Annals of Noninvasive Electrocardiology*, *Cardiovascular Digital Health Journal, Circulation: Arrhythmia and Electrophysiology*, *European Heart Journal - Digital Health and Journal of Arrhythmia* and to distribute it and/or communicate it to the public, either within the journals, or on its own, or with other related material throughout the world, in printed, electronic or any other format or medium whether now known or hereafter devised;
- *Annals of Noninvasive Electrocardiology, Cardiovascular Digital Health Journal, Circulation: Arrhythmia and Electrophysiology, European Heart Journal - Digital Health and Journal of Arrhythmia* will have the right to publish the Article under a CC-BY license, which allows free use of the Article, subject to correct attribution;
- to deposit copies of the Article in online archives maintained by Wiley Periodicals Inc., Elsevier Inc., Wolters Kluwer Health, Inc., Oxford University Press and John Wiley & Sons Australia, Ltd.
- make translations and reprints of the Article and to distribute them to the public;
- to authorize or grant licenses to third parties to do any of the above.

WILEY

In order to promote consistency between the publications, *Annals of Noninvasive Electrocardiology* will edit, typeset and proofread the Article with *Annals of Noninvasive Electrocardiology*'s style, as well as provide other parties with final word and PDF files at no cost to other parties.

The Article will be published in *Annals of Noninvasive Electrocardiology*, *Cardiovascular Digital Health Journal*, *Circulation: Arrhythmia and Electrophysiology*, *European Heart Journal - Digital Health* and *Journal of Arrhythmia* on such a date and time as further agreed in writing between all parties.

In addition to the terms and conditions detailed above, the copyright line in the co-publishing journals noted above shall read:

This article has been co-published with the permission of *Annals of Noninvasive Electrocardiology, Cardiovascular Digital Health Journal, Circulation: Arrhythmia and Electrophysiology, European Heart Journal - Digital Health and Journal of Arrhythmia*. All rights reserved. © 2020 The Authors. *Annals of Noninvasive Electrocardiology* published by Wiley Periodicals Inc. on behalf of the International Society for Holter and Noninvasive Electrocardiology / *Cardiovascular Digital Health Journal* published by Elsevier Inc. on behalf of the Heart Rhythm Society / *Circulation: Arrhythmia and Electrophysiology* published by Wolters Kluwer Health, Inc. on behalf of the American Heart Association, Inc. / *European Heart Journal – Digital Health* published by Oxford University Press on behalf of the European Society of Cardiology / *Journal of Arrhythmia* published by Wiley and Sons Australia Ltd on behalf of the Japanese Heart Rhythm Society and the Asia Pacific Heart Rhythm Society. This article is published under the Creative Commons CC-BY license, which permits use, distribution and reproduction in any medium, provided the original work is properly cited. The articles are identical except for minor stylistic and spelling differences in keeping with each journal's style. Either citation can be used when citing this article.

SIGNED on behalf of Wiley Periodicals Inc.

Shawn Morton
Shawn Morton (Oct 19, 2020 08:19 EDT)
....

Shawn Morton
Senior Editorial Director, Health Sciences

SIGNED on behalf of John Wiley & Sons Australia, Ltd

Chris Gray (Oct [illegible] 2020 16:46 GMT+11)
....

Chris Gray
VP, Knowledge and Learning, Asia Pacific

WILEY

SIGNED on behalf of Elsevier Inc.

Jane Grochowski
Jane Grochowski (Nov 3, 2020 07:26 MST)

Jane Grochowski
Senior Publisher
Health & Medical Sciences, STM Journals

SIGNED on behalf of Oxford University Press

G. Cannon
G. Cannon (Nov 3, 2020 09:08 GMT)

Gemma Cannon
Senior Publisher for the ESC journals

SIGNED on behalf of American Heart Association, Inc.

Denise Kuo
Director, Circulation Journals and JAHA
